# Effectiveness of a community-centered Newcastle disease vaccine delivery model under paid and free vaccination frameworks in southeastern Kenya

**DOI:** 10.1371/journal.pone.0308088

**Published:** 2024-08-01

**Authors:** Kennedy O. Ogolla, Douglas N. Anyona, Judith K. Chemuliti, Winnie W. Kimani, Francisca M. King’oo, Kennedy M. Waweru, Dalmas O. Omia, Isaac K. Nyamongo, Salome A. Bukachi

**Affiliations:** 1 Biotechnology Research Institute, Kenya Agricultural and Livestock Research Organization, Kikuyu, Kenya; 2 Institute of Anthropology, Gender and African Studies, University of Nairobi, Nairobi, Kenya; 3 School of Business and Economics, The Cooperative University of Kenya, Nairobi, Kenya; 4 Academics, Cooperative Development, Research & Innovation, The Cooperative University of Kenya, Nairobi, Kenya; UGHE: University of Global Health Equity, RWANDA

## Abstract

In the absence of effective drugs, vaccines constitute the cornerstone for the prevention of Newcastle disease (ND). Different strategies have been implemented to increase vaccination, but uptake remains low, underscoring the need for novel vaccine delivery methods. We designed and assessed the effectiveness of a community-centered ND vaccine delivery model in southeastern Kenya. Under the model, we sensitized smallholder chicken farmers (SCFs) through structured training on chicken husbandry, biosecurity, ND, and its vaccination, among other aspects. We subsequently engaged trained community vaccinators (CVs) to deliver vaccines and/or provide vaccination services to SCFs at a cost on one hand and, at no cost on the other, in selected sites to address challenges of inadequate service providers, vaccine unavailability, and inaccessibility. We tested this model under paid and free vaccination frameworks over one year and assessed the model’s effect on vaccine uptake, ND-related deaths, and vaccine accessibility, among other aspects. Overall, we vaccinated more chickens at free sites compared to paid sites. However, we vaccinated a significantly higher mean number of chickens per household at paid (49.4±38.5) compared to free (28.4±25.9) sites (t = 8.4, *p*<0.0001). We recorded a significant increase in the proportion of SCFs who vaccinated their chickens from 31.3% to 68.4% (χ^2^_(1, N = 399)_ = 58.3, p<0.0001) in paid and from 19.9% to 74.9% (χ^2^_(1, N = 403)_ = 115.7, p<0.0001) in free sites pre- and post-intervention, respectively. The mean number of ND-related deaths reported per household decreased from 18.1±31.6 pre-intervention to 7.5±22.3 post-intervention (t = 5.4, p = 0.000), with higher reductions recorded in paid sites (20.9±37.7 to 4.5±11.2) compared to free sites (15.0±22.6 to 10.7±29.7) pre- and post-intervention, respectively. Farmers with access to vaccines increased significantly from 61.1% to 85.4% (χ^2^_(1, N = 399)_ = 31.7, p<0.0001) in paid and 43.6% to 74.9% (χ^2^_(1, N = 403)_ = 38.4, p = 0.0001) in free sites pre- and post-intervention, respectively. We established that type of intervention framework, gender of household head, if the household head attended training on chicken production in the last 12 months, access to information on ND vaccination, and the number of chickens lost to the previous ND outbreak were significant predictors of ND vaccine uptake. Our findings indicate the model has a broader reach and benefits for SCFs. However, policies should be enacted to regulate the integration of CVs into the formal animal health sector.

## Introduction

The Newcastle disease (ND) causes huge economic losses to chicken farmers in sub-Saharan Africa (SSA) [1], and elsewhere around the world [2]. The disease is responsible for high morbidity and mortality in naïve flocks, disproportionately affecting smallholder chicken farmers (SCFs) in rural and marginal urban areas (RMUAs) [3]. It kills more village chickens than any other disease. The ND outbreaks, often heightened by poor biosecurity practices, can wipe out chickens in an entire region [4]. This not only deprives farmers of significant income but also has severe repercussions on households’ nutrition [5]. In the absence of effective drugs against ND, implementing routine vaccination and a strict biosecurity program constitute the cornerstones of ND prevention and mitigation [6,7]. While these practices are easy to execute on large commercial farms, their implementation in village flocks is often complicated by different flock sizes, age variations, and high mobility inherent in the free-range production system commonly practiced in RMUAs [8]. This problem is further compounded by widespread unregulated marketing of live birds and improper disposal of dead chickens [8].

While vaccines offer effective means of preventing ND and enhancing chicken productivity and profitability [7,9–11], structured training of SCFs on the appropriate biosecurity measures and carrying out regular ND vaccination are equally critical. Newcastle disease vaccines are largely available in agro-veterinary outlets (agro-shops) located in major urban and some marginal-urban centers in Kenya, yet the expansion of their reach, availability, and uptake among chicken farmers in RMUAs has not been realized. One of the contributors to low vaccination rates in rural areas has been the lack of an effective vaccine delivery system, rendering vaccines largely unavailable and inaccessible to many SCFs [12–14]. The ND vaccine delivery system is further hampered by factors such as the cost of access, limited availability of vaccine vending outlets in rural areas as well as an acute shortage of animal health service providers. The cost of ND vaccines contributes to the low uptake given that most SCFs in RMUAs have low purchasing power [15]. Thus, the SCFs resort to a few effective and/or ineffective alternatives at their disposal, such as the use of herbal remedies [14].

Despite efforts to expand vaccination coverage through the development of thermotolerant ND vaccines with longer shelf life [16], the vaccines remain unavailable and out of reach for many SCFs. Furthermore, the scarcity of agro-shops in RMUAs, compounded by challenges such as lack of electricity connection, frequent power outages, and the high operational costs associated with backup generators contribute to the high cost, unprofitability and unavailability of these vaccines. The power outages compromise vaccine potency and result in vaccine failure, which discourages farmers from subsequently vaccinating their chickens [17]. Animal health service providers, whenever available, can potentially bridge the gap between vaccine stockists and farmers by delivering vaccines and providing vaccination services to farmers. However, in many RMUAs of developing countries, the shortage and even lack of service providers persists [18–20], necessitating the need for innovative livestock vaccine delivery strategies. One promising approach involves use of trained community-based animal health workers (CBAHW) [21].

The community-based approaches are usually devised to support and complement the normally resource-strained, overstretched, and poorly functioning veterinary services in RMUAs of developing countries. In implementing community-based programs, countries have often adopted different methods tailored to tackle specific challenges experienced by farmers [21]. These programs involve short training of community-selected representatives, equipping them with technical skills to address crucial health or husbandry problems, and payment for their services is done directly by the farmers or indirectly by the government or other entities [4,21]. One such group of community-based animal health service providers are the community vaccinators (CVs), who deliver poultry vaccines and/or offer vaccination services to chicken farmers, especially in RMUAs [4]. In our recent study in southeastern Kenya, we established that the major contributors to low vaccine uptake were poor knowledge of ND and vaccines among farmers, long distances to vaccine vendors, vaccine unavailability and inaccessibility, farmer perceptions of vaccine efficacy and cost [14]. We, therefore, designed and tested the present model to address these aspects, the results of which are presented in this paper.

### Study conceptualization

Vaccine adoption is primarily the output of vaccine availability, accessibility, and demand [15]. However, challenges such as unavailability, inaccessibility, low coverage and high cost of ND vaccines, in addition to poor knowledge and perceptions among farmers around chicken vaccination persist in RMUAs. We, therefore, conceptualized a model to address these challenges centered on CVs. Under the model, we created a local pool of chicken vaccinators in the community to bridge the vaccine access gap in the last-mile delivery of ND vaccines, and equipped them with knowledge and skills to operate independently to continue providing vaccination services to SCFs beyond the project lifetime. We further instituted measures to ensure that the vaccines were available and accessible to CVs which were fundamental to the implementation of the model. In the conceptualization of the model, we incorporated the determinants of vaccine adoption described by Donadeu et al. [15], viz., 1) availability, 2) accessibility, and 3) demand. For purposes of this study, a) ‘vaccine availability’ was defined as the existence of safe and effective ND vaccines in adequate quantities and purchasable in the market; b) ‘vaccine accessibility’ entailed SCFs being able to obtain vaccines at affordable prices either directly from local agro-shops or indirectly through trained CVs or other animal health service providers, and c) ‘vaccine demand’ meant creating awareness of the existence of ND vaccines among SCFs to trigger their willingness to pay and use vaccines because they value the benefits of vaccinating chickens against ND [22]. We hypothesized that i) training chicken farmers on husbandry practices, biosecurity, and ND vaccination, coupled with mobilizing them to vaccinate chickens, will enhance the farmers’ knowledge, create awareness and stimulate demand for vaccines and vaccination services, and ii) the establishment of vaccine access points (VAP) in target communities close to the farmers and CVs will improve accessibility and availability, reduce transaction costs, and consequently increase vaccine uptake among SCFs.

## Materials and methods

### Study location and study sites

**Fig 1:**
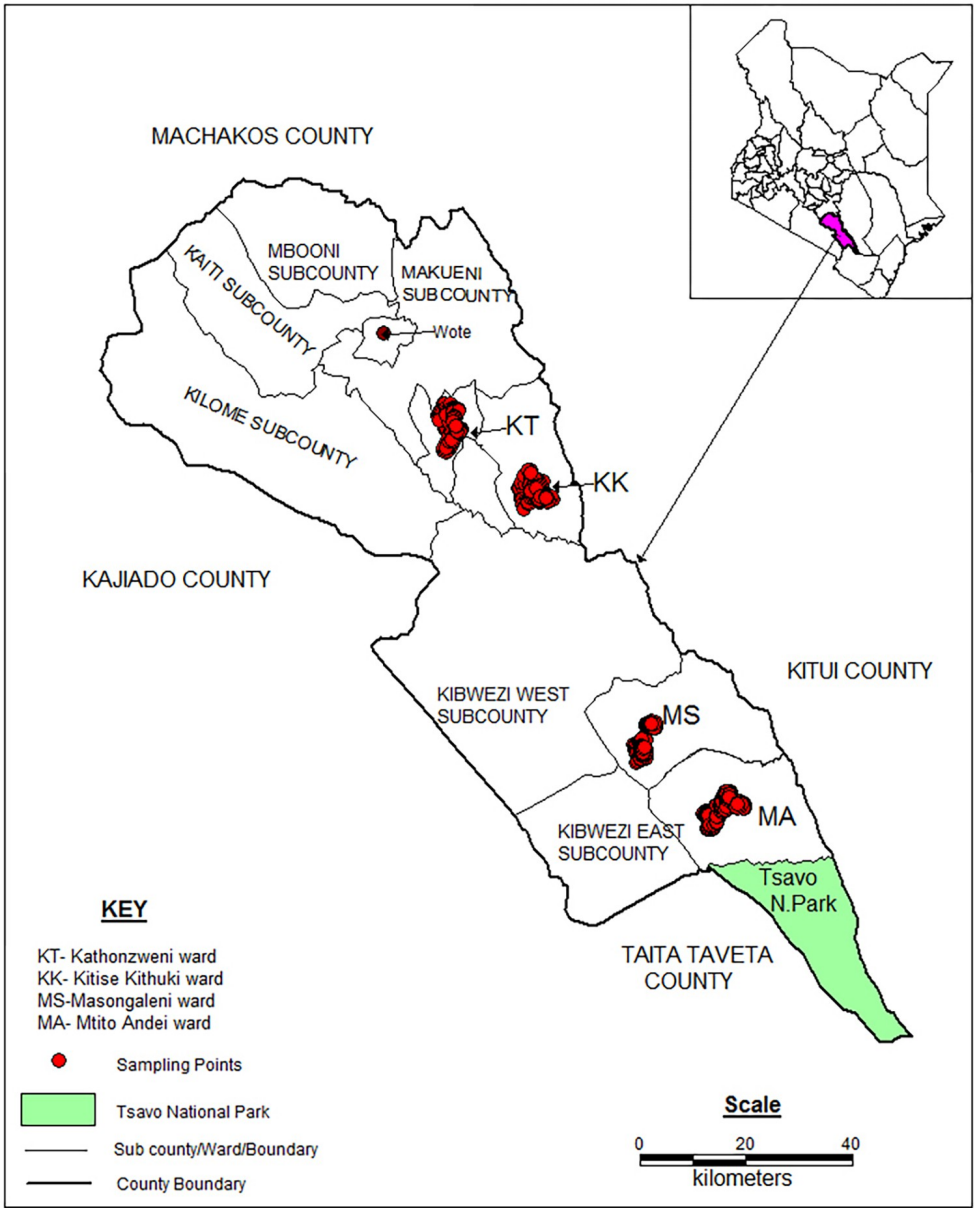
The study location and study sites.

We conducted the study in Makueni county, southeastern Kenya. Chicken production stands out as an important economic activity that supports many livelihoods in the region. However, smallholder farmers in Makueni endure difficulties with chicken diseases, particularly ND. The county is representative of chicken-rearing regions in Kenya and other Low and Middle-Income Countries. We carried out the study in four purposively selected administrative wards (Masongaleni, Mtito Andei, Kitise, and Kathonzweni) drawn from two sub-counties (Makueni and Kibwezi East) located in the drier lowlands of the county, where chicken production is the main livelihood activity (**Fig 1**).

To prevent overlaps or spillovers between the paid and free vaccination sites, we purposively selected the sub-counties, ensuring a substantial geographical buffer (provided by Kibwezi West sub-county) between Makueni and Kibwezi East sub-counties.

### Study population

The study population consisted of smallholder chicken-rearing households drawn from Kathonzweni and Kitise wards in Makueni sub-county, and Mtito Andei and Masongaleni wards in Kibwezi East sub-county. The selection of Kathonzweni and Kitise wards was based on their relatively close proximity to county headquarters (23–55 km) and were thus considered marginal urban areas. On the other hand, our choice of Masongaleni and Mtito Andei wards was driven by their remote location in far-flung rural areas, situated about 127–134 km from the county headquarters, and were categorized as rural areas. We used proximity to county headquarters as a proxy for accessing essential veterinary services and inputs, including service providers and vaccines. We enrolled farmers into the study on January 5, 2021, and followed them up until the completion of the study on December 20^th^, 2022, when the study was terminated.

### Description of the community-centered Newcastle disease vaccine delivery model

There are well-established and effective disease prevention and control vaccination schedules and biosecurity measures for commercial chicken production systems [7]. Attempts to replicate these strategies for extensive smallholder chicken production systems are not feasible and prove too costly for smallholder farmers with small flocks [8]. We assessed the effect of a community-centered Newcastle disease vaccine delivery model within both the paid and free vaccination frameworks on variables such as vaccine uptake, ND-related deaths, and vaccine accessibility, among others, in southeastern Kenya. We designed the model to mirror the recommended ND vaccination schedule for multi-aged free-ranging chickens, where birds are immunized every 12 weeks to boost their immunity and provide protection against the disease [23,24]. The model was operationalized by establishing vaccine access points (VAPs) in the community where farmers and CVs get their vaccines during a vaccination campaign. A VAP is a modest facility equipped with a refrigerator and a backup generator for storing vaccines operated by a technical staff for a month every three months. The facility also serves as a farmer advisory center, where farmers receive information and advice on improved chicken husbandry practices. The CVs are recruited from among community members, trained, and deployed as service providers. Awareness and demand for vaccines and vaccination services are created through farmer training and advertisement. The SCFs are sensitized and mobilized to access ND vaccines and/or vaccination services during the vaccination period. We describe the model in this paper, discuss its implementation and effectiveness, and share experiences learned to inform future implementation and/or improvement of the model for wide-scale adoption. The model was tested under both the paid and free vaccination frameworks, encompassing the following facets:

### Knowledge delivery

To influence change in attitudes and practices of SCFs towards the adoption of vaccines and appropriate biosecurity measures, we infused two knowledge delivery methods within the model: 1) classroom-based training coupled with a practical session, and 2) classroom-based training combined with a practical session and on-farm demonstration at selected model farms to facilitate peer-to-peer learning. We implemented the former in Kathonzweni (Makueni sub-county) and Mtito Andei (Kibwezi East sub-county) wards, and the latter in Kitise (Makueni sub-county) and Masongaleni (Kibwezi East sub-county) wards. We randomly selected SCFs from the four wards and trained them on chicken husbandry, biosecurity, and diseases, with emphasis on ND and its vaccination. We also trained the farmers on the importance of collectives (group formation and cooperatives), entrepreneurship, bookkeeping, nutrition, gender, and social norms. The training modules had practical sessions and demonstrations on vaccine handling, vaccine reconstitution and administration. In each sub-county, we randomly selected one administrative ward where farmers were also taken through on-farm demonstrations in model farms for peer learning alongside classroom-based training. The training manual for farmers is provided as supporting information in the **S1 Appendix**.

### Selection, training, and engagement of community vaccinators (CVs)

During the farmers’ training sessions, we adopted a participatory approach to select CVs. Farmers from the same village were asked to discuss and propose individuals from their village who could be trained as CVs using a criterion that they had generated. Earlier studies have established that community members are best suited to select individuals to serve them as vaccinators [4,21]. We trained between 19 and 24 chicken farmers as CVs in each ward using a community vaccinator training manual that we developed (**S2 Appendix**). The training covered basic information on the control and prevention of important chicken diseases in the region, with an emphasis on ND and its vaccination, ND vaccine reconstitution, vaccine administration, record-keeping, monetization of the vaccination service, effective mobilization for a successful vaccination campaign, and the importance of targeting both women and men farmers, among other aspects. The two-day training was divided into classroom-based settings and an interactive practical session combined with role plays. Trainees were taken through practical sessions using the ND vaccination kit and were assessed on their suitability to serve as CVs. Under this model, we expected the CVs to vaccinate and/or deliver ND vaccines to SCFs in their respective villages and vaccinating their chickens.

### Setting up of vaccine access points (VAP)

We set up four VAPs (one per site) and equipped them with a refrigerator and a backup generator to serve during power outages. We then recruited and trained one local vaccine attendant to take charge of each VAP (a detailed training guide is provided in the **S3 Appendix)**. We deliberately recruited attendants with animal health training backgrounds who, for that reason, had a basic grasp of chicken production, diseases, vaccine storage, and vaccination due to the sensitive nature of safeguarding vaccine potency. We tasked the attendants with issuing and/or selling ND vaccines to farmers or CVs and addressing their concerns, which they also captured for follow-ups by the project veterinarians. We collated and addressed the questions in our subsequent meetings with the farmers.

### The model under paid vaccination framework (PVF)

We implemented the PVF in Kathonzweni and Kitise wards within Makueni sub-county. Knowledge delivery with on-farm demonstration was implemented in Kitise ward, while knowledge delivery without on-farm demonstration was implemented in Kathonzweni ward. Under this framework, we transported ND vaccines alongside other supplies such as diluents, syringes, needles, and eye droppers to VAPs for sale to farmers and CVs. We provided the vaccines to farmers at a slightly reduced price of KSH 200 (1.56 USD) per 100-dose vial, slightly lower than the prevailing market price range of KSH 250–300 (1.95–2.36 USD) at the time. The SCFs had the option to either purchase the vaccines directly at the VAPs or to procure vaccination services from any of the self-employed, project-trained CVs in their respective villages. The CVs under this model ran their operations as independent businesses (without any additional facilitation from the project), offered services at a cost of KSH 5–10 (0.039–0.078 USD) per chicken vaccinated, and obtained their pay from the profit margins realized. However, we gave the CVs the first 5 vials at no cost as start-up support, after which they bought subsequent vials at the set price.

We carried out three vaccination rounds every 3 months as recommended for eye drop ND vaccination (25), each round running for 30 days. We conducted the first vaccination campaign between July 26, 2021, and August 31, 2021; the second campaign from November 8, 2021, to December 17, 2021; and the third from March 7, 2022, to April 3, 2022. The vaccine attendants captured data on the number of vaccine vials sold each day, the name of the farmer or CV who purchased the vaccine, the number of chickens kept by the farmer, the number of chickens to be vaccinated, and the name of the farmer’s village. We also tasked the CVs providing vaccination services with filling out forms to capture details on the number of chickens vaccinated, the route of vaccine administration, and whether or not they vaccinated all chickens in the household, among other details. The CVs returned the filled-out forms at the end of each day (the data collection form used by CVs is provided as supporting information **S4 Appendix**).

### The model under free vaccination framework (FVF)

We implemented the FVF in Masongaleni and Mtito Andei wards located in Kibwezi East sub-county. Knowledge delivery with on-farm demonstration was implemented in Masongaleni ward, while knowledge delivery without on-farm demonstration was implemented in Mtito Andei ward. Under this model, we transported and stored the vaccines and other supplies at VAPs in the two wards. Our project-trained research assistants, working alongside vaccine attendants, issued vaccines to the CVs and recorded the day’s vaccination data brought back by the CVs. Prior to each vaccination campaign, we identified and grouped all the villages in the study sites into clusters and assigned 2–4 CVs to work in each cluster, which happened to be either their respective villages or neighboring villages. We gave each CV a dust coat, a thermo-flask for vaccine storage and transportation, and a counterbook to capture vaccination data. Every morning during the vaccination campaign, we issued the CVs with ND vaccines, an eye dropper, 5 ml syringes, a 21G hypodermic needle and a diluent for reconstituting vaccines. We also gave the CVs a permanent marker at the start of each round for marking chickens that remained in a household in case the vaccine vials carried by the CV for the day got depleted before all the chickens were vaccinated.

Under this framework, we conducted vaccination at no cost, with the project facilitating the CVs. The *modus operandi* involved the CVs picking up the vaccines early in the morning (from 6.00 a.m. to 6.30 a.m.) at VAPs, walking into homesteads already pre-informed by village elders, and vaccinating chickens until the vials allocated for the day were depleted (mostly around 10–11 a.m.). Being mostly new in the trade, we tasked the CVs with vaccinating at least 100 chickens per day, depending on the number of households mobilized by the respective village elders. In each household covered, the CVs recorded the name and contact information of the household head, the number of chickens kept, the number of chickens vaccinated, sick chickens seen if any, and the reason for not vaccinating all or some chickens (if any), among other aspects (see supporting information in the **S4 Appendix**). We used I-2 NDV thermotolerant and LaSota NDV thermolabile strains of ND vaccines, both recommended for application via ocular, nasal, and oral routes by the manufacturers. However, we used ocular or nasal routes in our campaigns to ensure all chickens in the household were vaccinated. The implemented model is summarized in **Table 1.**

**Table 1:** Community centered vaccination model under paid and free frameworks.

Community based ND vaccination model	Description of vaccine delivery model	Knowledge delivery	Study sites
Paid Vaccination Framework (PVF)	○ ND vaccines were delivered at a cost to CVs and SCFs ○ Farmers had access to vaccines either directly or through project-trained CVs	Peer education	Kathonzweni ward
		Peer education + farm demonstrations	Kitise ward
Free Vaccination Framework (FVF)	○ ND vaccines were delivered at no cost (free of charge) to farmers ○ Door-to-door ND vaccination carried out by project-trained CVs	Peer education	Mtito Andei ward
		Peer education + farm demonstrations	Masongaleni ward

### Creation of demand through mobilization and advertisement

We created demand and awareness for ND vaccines among SCFs by employing various mobilization and advertisement strategies tailored to each site. In paid vaccination sites, we used a public address system mounted on a vehicle to inform farmers of vaccine availability, point of sale, cost per vial, duration of the vaccination campaign, and importance of ND vaccination. We strategically scheduled three advertisements during each vaccination campaign within specific intervals: before the start, in the middle, and towards the end of the 1-month vaccination period. We also mounted large banners and posters at entry points to VAPs and at strategic locations within the wards such as market centers and water collection points frequented by farmers. In free vaccination sites, we mobilized chicken farmers using local administrators (village elders, assistant chiefs, and chiefs). We further informed the households we had earlier trained about the availability of vaccines via phone calls. We also mobilized through announcements in local schools, churches, farmers’ groups, and agricultural cooperatives.

### Supervision and monitoring of CVs performance

We supervised vaccine storage, issuance, sale, and administration during the vaccination campaign. We followed the CVs on separate days and observed how they conducted the vaccination exercise in different households. Subsequently, we evaluated their performance using a structured guideline (**S5 Appendix)**. We instructed the CVs to vaccinate healthy chickens of at least 14 days of age or older and to avoid vaccinating chickens in homesteads with suspected ND infections or sick chickens. As a biosafety precaution, the CVs washed and disinfected their hands and feet between farms to prevent potential transmission of infectious pathogens from one home to another. Additionally, the CVs were instructed to advise farmers on general chicken husbandry practices.

### Appraisals of CVs and farmers’ satisfaction assessment

Borrowing from published tools for evaluating the performance of para-veterinary professionals, such as the OIE PVS Tool and participatory tools used elsewhere [25,26], we designed two appraisal tools, one used to assess the performance of CVs (**S6 Appendix**) and the other to evaluate the satisfaction of SCFs with the services received from CVs (**S7 Appendix**).

### Pre-intervention and post-intervention surveys to assess the model effect

In recruiting the intervention group, we initially assessed the eligibility of 841 SCFs to participate in the intervention and excluded 365 from the study for various reasons detailed in the CONSORT figure provided as **S8 Appendix**. We assessed the effect of the model on eligible participants by comparing vaccine uptake, ND-related deaths, and ND knowledge, among other variables, pre- and post-intervention. We randomly administered a structured questionnaire to 476 respondents at baseline (January-February 2020), and successfully re-administered the questionnaire to 404 of these respondents in the post-intervention survey (November-December 2022) to generate data on various aspects of the vaccine delivery model. We administered the questionnaires via face-to-face interviews using trained research assistants recruited from the study location and who were proficient in the native language. The questionnaire captured data on vaccination history, chicken breeds kept, ND-associated losses, ND knowledge, and access to ND vaccines, among other aspects (**S9 Appendix**). The same respondents in the households were interviewed pre- and post-intervention using the same questionnaire. The questionnaire was digitized in the ODK Collect software and administered using tablets. We first pretested the questionnaires on a few SCFs in Wote town within Makueni county (**Fig 1**). The interviews were conducted in the native Kamba language, English or Swahili, depending on the farmer’s preference, and the responses were captured in English in the ODK Collect software.

### Data management and analysis

We imported the coded data into SPSS version 21 for analysis. We calculated overall attrition, and then conducted a proportions test for trial attrition differences across the two frameworks to rule out attrition bias. To further ascertain that attrition did not affect the study outcome, we compared selected demographic characteristics such as respondent sex, number of participants from each sub-county and ward pre- and post-intervention to detect any significant differences if any. Descriptive statistics, including means ± standard deviations (SD) for continuous variables, and frequencies with their percentages for categorical variables were used to summarize the data. We carried out proportion and association tests of categorical variables using Chi-square (χ^2^) to show the effect of the vaccination model pre- and post-intervention between and within paid and free vaccination frameworks. Statistical tests of significance comparing ND-related deaths pre- and post-intervention were carried out using Student’s t-tests. To correct for any differential non-participation in the intervention frameworks tested, we applied the inverse probability of treatment weighting approach to our binary logistic regression. We generated predicted probabilities and weights from our selected model of treatment with the type of vaccination framework received as a dependable variable and characteristics such as gender, ward, access to vaccine supplier, and access to training seminars among others being independent variables. We calculated the weight of each participant as the inverse of the predicted probability of receiving the intervention the participant received based on our observed covariates. We then fitted a Generalized Linear Model (GLM) with binomial probability distribution integrating binary logistic link function to establish any association between ward (Masongaleni, Mtito Andei, Kitise, Kathonzweni), or gender of HH (men, women) or access to vaccine supplier (yes, no) or ability to afford vaccine (yes, no), or access to cold chain (yes, no), or access to training seminars (yes, no), or perception that vaccine can offer chickens protection (yes, no), or had access to ND vaccination information (yes, no), or can vaccinate (yes, no) or keeps small ruminant (yes, no), or keeps large ruminants (yes, no), or access to training in the last 12 months (yes, no), or number of chickens lost to ND in the previous year as independent variables and vaccination of chickens by households (yes, no) as dependent covariates. We excluded data that did not fit the model and ran a controlled GLM. We compared chicken vaccination numbers in the three vaccination campaigns to show the impact of the intervention models on vaccine uptake.

### Ethical approval

Ethical approval (SU-IERC0523/13) was obtained from the Strathmore University Institutional Ethics Review Committee while the study license (NACOSTI/P/19/1207) was issued by the National Commission for Science, Technology, and Innovation.

### Informed consent

We obtained written informed consent from each respondent before initiating the study. The consenting process involved providing the respondents with adequate information about the study, outlining the potential benefits and consequences of participation, addressing questions from the respondents, assuring the respondents of their right to discontinue the interview at any point, allowing ample time for the respondents to make a decision, and finally obtaining their voluntary agreement by way of a signature or thumbprint on the informed consent form (in duplicate). One copy of the consent form was left with the participant and the other was retained for documentation.

## Results

### Demographic characteristics of SCFs and CVs

We trained 372 SCFs comprising women (74.6%) and men (25.4%). The average age of the trained farmers was 49.2±14.2 years, with women on average being younger (47.6±13.3 years) than men (54.7±16.0 years). Of these farmers, 3.0% were people living with a disability. We further trained 124 CVs selected from the farmers, mostly comprising women (80.6%). The mean age of the CVs was 42.8 years (range: 18–75 years). Of the CVs, 81.4% were from male-headed households and 18.6% from female-headed households. Most of the CVs (55.8%) had a primary level of education, 37.2% had a secondary level, with the remaining having college (2.3%), university (2.3%), and vocational (2.3%) levels. The main source of income for the CVs was farming (74%), followed by business (13%), informal employment (7%), remittances (3%), and formal employment (3%). Disaggregation of the trained SCFs and CVs by gender and ward under each vaccination framework is presented in **Table 2**.

**Table 2:** Trained SCFs and CVs by gender and ward under each framework.

Vaccination framework	Ward (sites)	Number of trained smallholder chicken farmers		
		Women	Men	Total
PVF	Kathonzweni	51 (78.5%)	14 (21.5%)	65 (100%)
	Kitise	79 (86.8%)	12 (13.2%)	91 (100%)
FVF	Masongaleni	81 (77.1%)	24 (22.9%)	105 (100%)
	Mtito Andei	79 (71.2%)	32 (28.8%)	111 (100%)
	**Total**	**290 (78.0%)**	**82 (22.0%)**	**372 (100%)**
		**Number of trained community vaccinators**		
PVF	Kathonzweni	15 (78.9%)	4 (21.1%)	19 (100%)
	Kitise	16 (80.0%)	4 (20.0%)	20 (100%)
FVF	Masongaleni	19 (79.2%)	5 (20.8%)	24 (100%)
	Mtito Andei	18 (75.0%)	6 (25.0%)	24 (100%)
	**Total**	**68 (78.2%)**	**19 (21.8%)**	**87 (100%)**

PVF–Paid Vaccination Framework, FVF-Free Vaccination Framework.

### Chicken vaccination numbers under paid and free vaccination frameworks

We vaccinated a higher number of chickens in the first round of vaccination (94,661) compared to the second (78,250) and third (84,649) rounds under the model, translating to an average of 30.2±27.9 chickens vaccinated per household in both paid and free vaccination sites. By comparison, we vaccinated more chickens and had wider vaccine coverage in the free vaccination sites, manifested by the high number of households reached. Specifically, we vaccinated significantly more chickens at no cost under the FVF (226,854) relative to the PVF (30,755) sites in the three rounds. In contrast, we reached relatively fewer households yet vaccinated a significantly higher mean number of chickens per household at paid (49.36±38.5) compared to free (28.38±25.9) vaccination sites (t = 8.3864, p < .0000). Kathonzweni ward recorded the highest mean number of chickens vaccinated per household (69.9±48.5) relative to Kitise ward (39.0±27.2 chickens), both under PVF, and still higher than the average numbers per household under FVF. We recorded a significant increase in the mean number of chickens vaccinated per household from 33.7±29.3 in the first round to 42.4±40.7 in the second and 49.4±38.5 in the third vaccination rounds (p<0.005) under the PVF. On the other hand, we vaccinated an average of 28.9, 25.7, and 28.4 chickens per household in the first, second, and third vaccination rounds, respectively, under the FVF. The highest increase in chicken holding per household was recorded in Kathonzweni (a PVF site), with the mean number of chickens vaccinated per household increasing from 33.6 to 49.7 and finally to 69.9 chickens in the first, second, and third vaccination campaigns, respectively. We recorded a decline in the number of households that vaccinated their chickens in both paid (by 18.3% and 11.1% between the first and second, and first and third rounds, respectively) and free vaccination sites, by 5.6% and 7.1% between the first and second, and first and third rounds, respectively. **Table 3** summarizes the vaccination numbers for the three vaccination campaigns.

**Table 3:** Number of chickens vaccinated under paid and free vaccination frameworks.

Vaccination numbers under PVF				
Vaccination	Ward	No. of chicken vaccinated	No. of households reached	Mean No. of chicken per household
Round 1	Kathonzweni	2,855	89	33.6 ±26.7
	Kitise	6,079	190	33.8 ±30.5
Round 2	Kathonzweni	5,863	133	43.1±40.3
	Kitise	3,217	95	41.2±41.6
Round 3	Kathonzweni	5,804	79	69.9±48.5
	Kitise	6,437	169	39.0±27.2
**Total (Average)**		30,255 (5,042)	755 (126)	41.6±36.7

**Vaccination numbers under PVF**

Round 1	Masongaleni	31,427	1,238	25.4±24.4
	Mtito Andei	47,806	1,509	31.7±51.7
Round 2	Masongaleni	35,728	1,540	23.2±25.3
	Mtito Andei	30,330	1,053	28.8±36.1
Round 3	Masongaleni	38,714	1,381	28.0±28.9
	Mtito Andei	33,694	1,170	28.8±22.0
	**Total (Average)**	**217,699**	**7,891**	**27.6±33.6**

### Assessing effectiveness of the model

**Fig 2:**
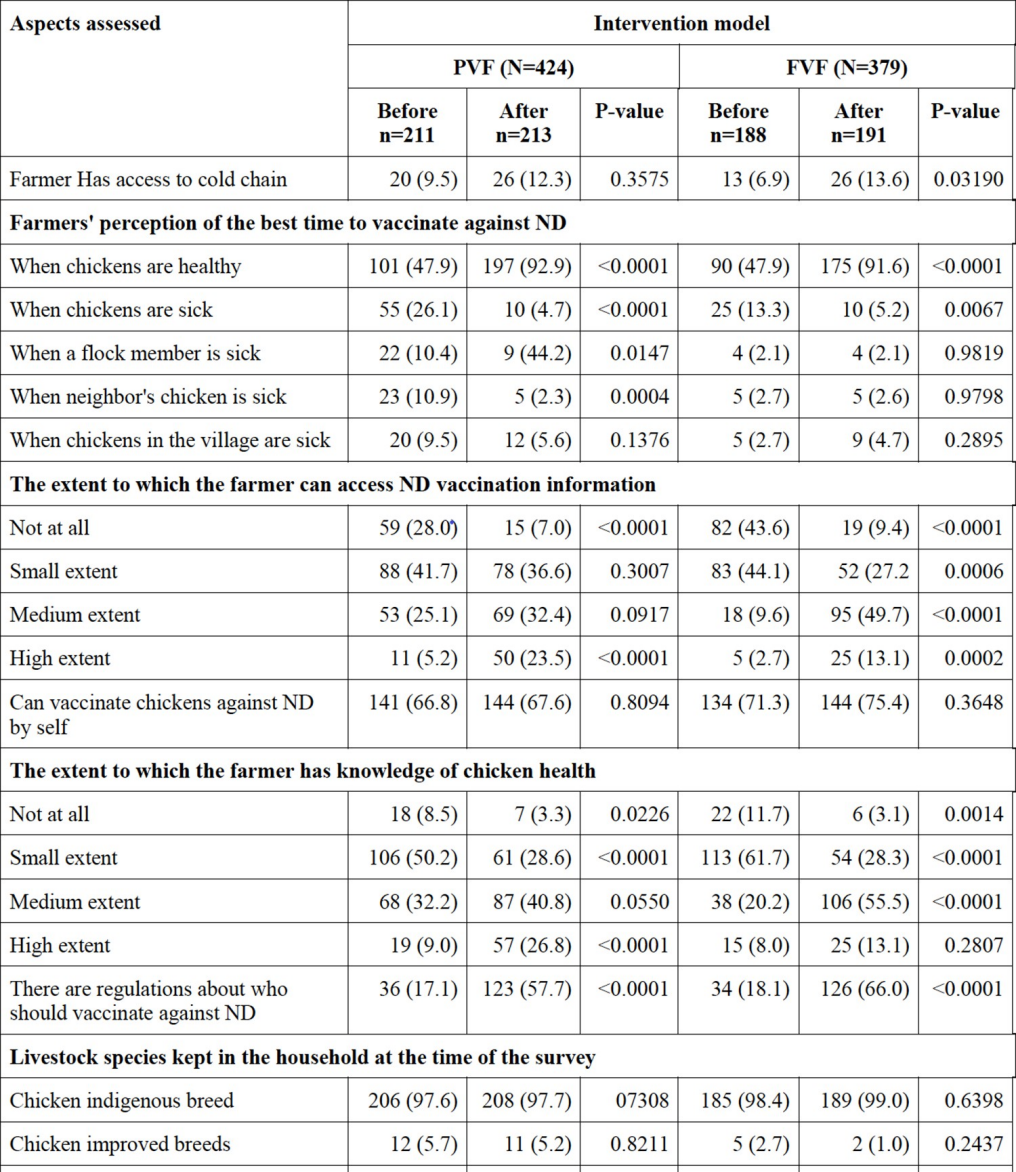
Summary results of the aspects assessed before and after the intervention.

We recorded an overall attrition rate of 15.1% from 476 to 404 interviewed farmers before and after the intervention, respectively, for reasons detailed in the **S8 Appendix**. Attrition rates under paid and free vaccination frameworks were 13.9% and 16.4%, respectively, translating to a differential attrition of 2.5%. We then applied the attrition threshold under cautious and optimistic assumptions. With an overall attrition of 15.1%, the maximum allowable differential attrition under the cautious threshold was 5.9%, while the maximum allowable differential attrition under the optimistic threshold was 10.7%. This means our differential attrition of 2.5% was low and within acceptable limits. We did not detect any significant difference in the demographic variables we tested such as sex of respondent, mean numbers of participants from each ward and sub-county pre- and post-intervention indicating the absence of attrition effect. Our GLM analysis with inverse probability weighting revealed that the type of intervention arm (χ^2^_(1, N = 379)_ = 22.3, p = 0.000), gender of household head (χ^2^_(1, N = 379)_ = 3.7, p = 0.042), household head attending a training on chicken production in the last 12 months (χ^2^_(1, N = 379)_ = 13.9, p = 0.000), access to information on ND vaccination (χ^2^_(1, N = 379)_ = 21.7, p = 0.000), keeping large ruminants (χ^2^_(1, N = 379)_ = 4.8, p = 0.029) and number of chickens lost to the previous ND outbreak (χ^2^_(1, N = 379)_ = 4.6, p = 0.032) were significant predictors of vaccine uptake among smallholder farmers in the study area. On the other hand, access to a vaccine supplier (χ^2^_(1, N = 379)_ = 2.5, p = 0.114), would like to have access to vaccine supplier (χ^2^_(1, N = 379)_ = 0.7, p = 0.402), ability to afford vaccine (χ^2^_(1, N = 379)_ = 4.6, p = 0.1), access to the cold chain (χ^2^_(1, N = 379)_ = 3.1, p = 0.079), think vaccine can prevent ND (χ^2^_(1, N = 379)_ = 1.2, p = 0.559), has knowledge on chicken health (χ^2^_(1, N = 379)_ = 7.1, p = 0.069), can vaccinate ND (χ^2^_(1, N = 379)_ = 4.0, p = 0.135), keeping small ruminants (χ^2^_(1, N = 379)_ = 2.3, p = 0.128), or keeping improved chicken breeds (χ^2^_(1, N = 379)_ = 1.0, p = 0.321) were not significantly associated with vaccine uptake. Specifically, we recorded a significant increase in the proportion of farmers who vaccinated their chickens from 31.3% to 68.4% (χ^2^_(1, N = 424)_ = 58.27, p<0.0001) in paid sites and from 19.9% to 74.9% (χ^2^_(1, N = 379)_ = 115.71, p<0.0001) in free sites pre- and post-intervention, respectively. The mean number of chickens lost to ND-related deaths reduced significantly from 18.1±31.6 pre-intervention to 7.5±22.3 post-intervention (t = 5.449, p = 0.000), with higher reductions recorded in paid sites (20.9±37.7 to 4.5±11.2) compared to free sites (15.0±22.6 to 10.7±29.7) pre- and post-intervention, respectively. Farmers with access to vaccines increased significantly from 61.1% to 85.4% (χ^2^_(1, N = 424)_ = 31.7, p<0.0001) in paid sites and from 43.6% to 74.9% (χ^2^_(1, N = 379)_ = 38.4, p = 0.0001) in free sites pre- and post-intervention, respectively. The SCFs who reported that vaccines were effective in preventing ND significantly increased from 75.4% pre- to 96.0% post-intervention in paid sites (χ^2^_(1, N = 424)_ = 42.6, p<0.0001), and 74.5% pre- to 94.2% post-intervention (χ^2^_(1, N = 379)_ = 28.2, p<0.0001) in free sites under the model. In contrast, the proportion of farmers who reported they didn’t know whether vaccines were effective in preventing ND significantly reduced from 20.6% pre- to 2.7% post-intervention. Similarly, the farmers who reported they would like to have access to ND vaccines significantly reduced from 35.1% pre- to 9.9% post-intervention in paid vaccination sites (χ^2^_(1, N = 424)_ = 38.5, p<0.0001), and from 53.4% pre- to 22.5% post-intervention in free vaccination sites (χ^2^_(1, N = 379)_ = 38.7, p<0.0001). The SCFs who could afford to purchase ND vaccines significantly increased from 60.7% pre- to 85.8% post-intervention in paid sites (χ^2^_(1, N = 424)_ = 34.3, p<0.0001), and 44.7% pre- to 69.1% post-intervention in free sites (χ^2^_(1, N = 379)_ = 23.1, p<0.0001). The SCFs with knowledge of where to purchase ND vaccines significantly increased from 70.6% pre- to 85.8% post-intervention in paid sites (χ^2^_(1, N = 424)_ = 14.4, p = 0.0001), and 50.0% pre- to 81.2% post-intervention in free sites (χ^2^_(1, N = 379)_ = 40.8, p<0.0001). Regarding knowledge of chicken health, most of the farmers reported they were knowledgeable about chicken diseases to a medium extent (47.9%) and a high extent (20.3%) post-intervention, compared to 26.6% and 8.4% for medium and high extent, respectively, recorded pre-intervention. Conversely, the proportion of farmers that had no knowledge (10.0%) or had little knowledge (54.9%) of chicken health pre-intervention was higher than the 3.2% (no knowledge at all) and 28.5% (little knowledge) recorded post-intervention. The SCFs with access to training seminars and information on chicken production significantly increased from 30.3% pre- to 67.5% post-intervention in paid sites (χ^2^_(1, N = 424)_ = 58.3, p<0.0001), and 16.0% pre- to 63.4% post-intervention in free sites (χ^2^_(1, N = 379)_ = 88.8, p<0.0001). Detailed results are provided in **Fig 2**.

**Fig 3:**
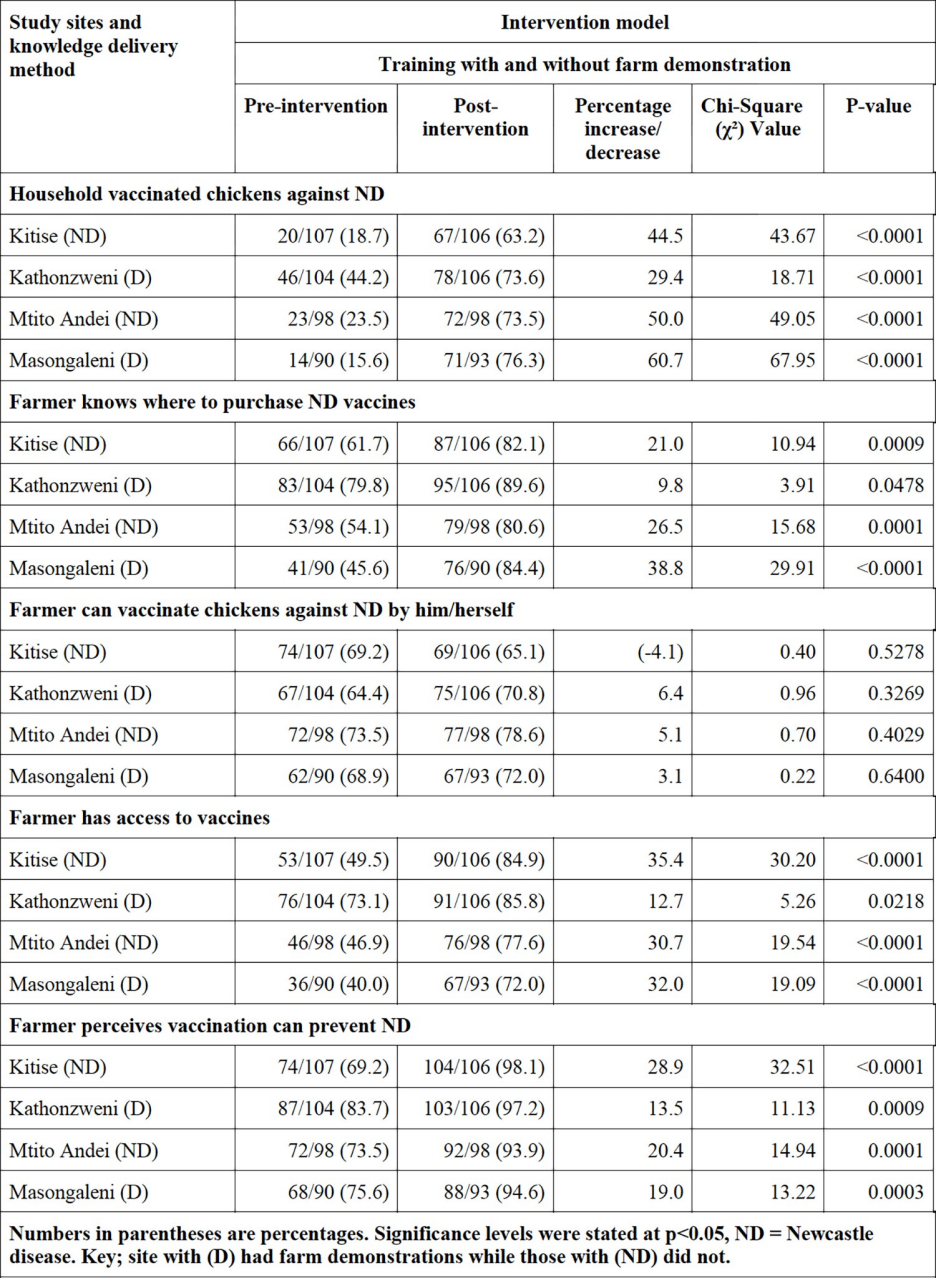
Summarized results of the knowledge delivery methods.

There were discernible variations in the effect of the model based on the knowledge delivery method used. We recorded higher vaccination numbers, increased numbers of farmers aware of where to purchase ND vaccines, and increased numbers of farmers with access to ND vaccines in sites where farmers’ training was complemented with on-farm demonstrations compared to sites with farmers’ training alone, as presented in **Fig 3**.

### Reasons for not vaccinating

The reasons for refusing to vaccinate all or some of the chickens by the SHCFs are presented in **Table 4**.

**Table 4:** Reasons given for declining vaccination service in free vaccination sites during the three vaccination rounds.

No.	Reason for not vaccinating	The proportion of chicken farmers		
		Round 1	Round 2	Round 3
1.	Chickens escaped	345 (70.0%)	230 (69.7%)	138 (61.9%)
2.	Sick chicken	65 (13.2%)	49 (14.8%)	35 (15.7%)
3.	Under medication	1 (0.2%)	0 (0.0%)	0 (0.0%)
4.	Not confined	23 (4.7%)	8 (2.4%)	19 (8.5%)
5.	Already vaccinated by farmer	7 (1.4%)	0 (0.0%)	3 (1.3%)
6.	The owner refused/ declined	4 (0.8%)	2 (0.6%)	0 (0.0%)
7.	Vaccine was depleted	47 (9.5%)	33 (10.0%)	21 (9.4%)
8.	Chicks less than 2 weeks	1 (0.2%)	8 (2.4%)	7 (3.1%)
	**Total**	**493 (100%)**	**330 (100%)**	**223 (100%)**

### Assessment of the community vaccinators

Of the 82 trained CVs, 61, 65, and 68 participated in the first, second, and third rounds of vaccination, respectively. Of the 39 CVs trained under PVF, 22, 22, and 26 participated in the first, second, and third vaccination campaigns, respectively. All 48 trained CVs under FVF participated in the first vaccination campaign. However, the number dropped to 44 in the second and third vaccination campaigns, translating to an attrition rate of 8.3% in the one-year vaccination period. Most of the CVs (85.0%) under FVF administered the ND vaccine via the ocular route compared to the nasal route (11%), with about 4.0% using both the ocular and nasal routes in the first vaccination round. In the subsequent rounds, 76.7% and 87.5% of the CVs used the ocular route during the second and third rounds of vaccination, respectively, compared to 21.5% and 10.6% who used the nasal route in the second and third vaccination rounds, respectively. Most CVs performed well on most of the aspects assessed, as detailed in **Table 5**.

**Table 5:** Community vaccinators’ performance appraisal.

Aspects assessed	Total (n = 38)
Transported vaccines properly (not exposed to sunlight & heat)	38 (100%)
Picked vaccines in good time (6.30–7.30 am)	38 (100%)
Finished the job early before the ice melts	38 (100%)
Met target of 100 chickens per day	38 (100%)
Administered vaccines correctly in the eye or nostril	36 (94.7%)
Reconstituted vaccines correctly	36 (94.7%)
Handled farmers with respect when they visit their homesteads	36 (94.7%)
Took accurate and legible records	35 (92.1%)
Did not expose vaccines to sunlight during reconstitution and administration	34 (89.5%)
Washed hands between household	17 (44.7%)
Provided poultry husbandry information and advice to farmers	5 (13.2%)

However, only a small proportion of the CVs offered peer education and advice to farmers on chicken husbandry (13.2%), and 92.1% took accurate and legible records or washed hands between households (44.7%).

## Discussion

We implemented a community-centered ND vaccine delivery model anchored around CVs and assessed its effectiveness on vaccine uptake and ND-related deaths, among other aspects. We designed the model to address challenges around vaccine availability, accessibility, and demand against the many challenges faced in RMUAs. Our findings suggest that the model can have a broader reach and provide greater benefit to SCFs if properly implemented. We anchored the model around CVs who were selected from the community by their peers, trained by the project, and either operated independently (in marginal urban areas) or were facilitated by the project (in rural areas) to address the problem of inadequate and/or lack of animal service providers in RMUAs and bridge the gap in last-mile delivery of chicken vaccines. The critical role played by community-based animal health workers in complementing the overstretched and sometimes inadequate or entirely lacking veterinary personnel in RMUAs of developing countries is well documented [15,27,28].

While the model shows potential for stimulating the uptake of chicken vaccines, the involvement of CVs may present several challenges in some regions. First, there is a lack of legal framework and policy in some countries, like Kenya, to support the operations of CVs [21,29]. Secondly, the modalities used to engage CVs and negative perceptions on the quality of services they offer can deter their acceptability leading to a reluctance by farmers to procure their services. For instance, a study conducted in Ghana reported that farmers preferred government-employed animal health service providers to CBAHWs because of claims of poor performance attributed to limited knowledge [30]. In our study, only a few CVs offered husbandry information to farmers and adhered to biosecurity protocols, such as washing hands between farms, which may potentially contribute to disease transmission during vaccination campaigns.

In the present study, we recorded a significant increase in the number of chickens vaccinated, a wider vaccine coverage manifested by the number of households reached, and a higher proportion of farmers vaccinating their chickens before and after intervention in free sites compared to paid sites. Other than a few households that declined our vaccination services, we covered over 90% of households keeping chickens at the free vaccination sites. In contrast, we reached relatively fewer households but vaccinated a higher mean number of chickens per household at the paid sites compared to the free sites. This may be attributed to the fact that households with more chickens are likely to vaccinate their chickens due to the high stakes involved should a disease outbreak occur [31]. A higher number of chickens in a household could also suggest a greater knowledge of chicken health, potentially increasing the likelihood of vaccinating if vaccines are available.

Sustainability poses a significant challenge for community-based animal health delivery models [29], as their success is hinged on their ability to guarantee that CVs will continue to make a profit from their work or have a way of remunerating the CVs for the services rendered [4]. In the present study, we offered vaccines at a slightly reduced price under PVF to help the CVs realize reasonable profit margins and fully facilitated the CVs in the free vaccination sites. Although models such as ours that provide incentives to reduce cost and maximize profits can be effective in stimulating vaccine uptake, they can be cost-prohibitive and not sustainable in the long run [15], especially when the models are heavily subsidized, like the no-cost vaccines under our FVF. Apart from the involvement of CVs, we attribute the high vaccination numbers to effective mobilization and the advertisements carried out during the triannual vaccination campaigns, as well as the sensitization and training offered to the farmers at the beginning of the intervention.

We noted deficiencies in the handling, reconstitution, and administration of vaccines by some CVs during the campaigns. Consequently, we aver that the effectiveness of this model requires close supervision of CVs as they work to mitigate potential lapses in their operations. This can be achieved using several means such as collecting the views of farmers through customer satisfaction feedback surveys, monitoring the CVs during their operations, and making the requisite adjustments in the course of the vaccination campaign. An intricate part of our model was the engagement of CVs to provide peer-to-peer learning to farmers apart from offering vaccination services. The CVs could not effectively perform this role as we envisaged in the model. We observed that this role requires a high level of expertise in chicken husbandry and diseases that are beyond the scope of the training the CVs received, given that the farmers had a wide range of questions on livestock production that could only be handled by experts in the field.

From our experience, we are convinced that the success of this model is predicated on several factors being met. First, is the establishment of an efficient vaccine delivery system that guarantees vaccine availability and accessibility in VAPs. The delivery system should be able to link the manufacturers and distributors of vaccines to stockists, service providers, and farmers [15]. At the core of a functional vaccine delivery system are cold chain facilities that safeguard vaccine potency. Setting up and maintaining these facilities in RMUAs in the face of frequent power outages can be expensive [32], especially since most RMUAs where ND is endemic are not connected to the national electricity grid. As a result, the sustainability of this model in rural areas requires a high initial capital injection from the government and/or development partners. However, aiding the agro-shops in RMUAs to acquire solar-powered refrigerators can reduce the associated costs, hence making vaccines available and accessible, and thereby enhancing uptake.

Second, vaccine cost is another important contributing factor to low vaccine uptake in rural areas [33]. The high cost of vaccines and high dose formats discourage farmers, particularly the majority who rear few chickens (typically less than 30 per household) from vaccinating [14]. The 100-dose vial is the most common ND dose format in Kenya. When additional expenses related to the acquisition of vaccines, such as transportation fees to and from agro-shops, are taken into account, the cost of vaccination can escalate to levels beyond the reach of many SCFs. Even when the cost of vaccines appears fair, the high-dose formats often discourage farmers with few chickens from purchasing vaccines [34]. It has been shown that presenting ND vaccines in low-dose formats can increase vaccine uptake among SCFs in rural areas [35]. Alternatively, organizing farmers in formal groups or cooperatives can also help them pool their resources, purchase high-dose format vaccines, and vaccinate their chickens by taking advantage of economies of scale [36,37].

Another key determinant of vaccine uptake is the demand for vaccines [13,15]. This can be achieved through sensitization, mobilization, and advertisement designed to change the farmers’ practices, perceptions, and attitudes toward ND vaccine use. Designing vaccination programs that address the specific needs of the community has been shown to have a greater impact on increasing vaccine uptake [15]. We recorded increased vaccine uptake in the present study by infusing training, mobilization, and advertisement into our model. This further underscores the vital role that knowledge plays in driving behavioral change in a community. Indeed, it has been established that training farmers on good chicken husbandry also improves their biosecurity compliance [38].

We recorded fewer ND-related deaths after we intervened in the present study. It has previously been demonstrated that better survival of chickens leads to increased chicken numbers and better returns, further stimulating increased demand for vaccines [11]. High demand guarantees continuous stocking of vaccines by agro-shops and uptake by SCFs, thus making the model sustainable. We have shown that this model is likely to increase vaccine adoption, as demonstrated by the steady and significant increase in the number of vaccinated chickens and reductions in chicken deaths recorded under the free and paid frameworks. Therefore, fitting this model to meet region-specific needs can guarantee bulk purchases of vaccines at relatively lower prices during the triannual vaccination campaigns. The model also makes sensitization, mobilization, and advertising for vaccination easy and inexpensive. Furthermore, the model can aid in quantifying the annual demand for vaccines in a region or country which can serve as a guide for manufacturers in determining their optimal production quantities. A closely related approach has been successfully used to increase the uptake of rabies vaccines in Africa [39,40].

During the implementation of the study at the free vaccination sites, we established other subtle but significant determinants of vaccine uptake besides cost. These included prevailing cultural and societal prejudices around vaccines and community suspicions surrounding ‘free’ services. Therefore, for this model to succeed under FVF, a robust mechanism is required for countering existing prejudices about the use of vaccines and misinformation arising during the vaccination campaign, as these can derail the campaign. We observed a few cases of vaccine hesitancy stemming from misinformation about the vaccines, which influenced some households to decline our vaccination services. Some households attributed post-vaccination chicken deaths to the vaccines, resulting in the rejection of vaccines by farmers in subsequent vaccination rounds. Thus, as part of the model, we recommend carrying out ND surveillance and training chicken farmers before carrying out vaccination campaigns to avert similar occurrences.

## Conclusion

We have shown that a community-centered vaccination model where farmers are sensitized and mobilized to access and pay for vaccines and/or vaccination services can have a broader reach and benefit for SCFs. While the paid and free frameworks are both effective in increasing uptake, the paid option appears to be cost-effective and sustainable. For practical implementation, we recommend employing the free vaccination framework in remote rural areas with poor to almost non-existent veterinary infrastructure, and the paid vaccination framework can be applied in marginal urban and urban regions where veterinary systems such as agro-veterinary outlets are already established. We recommend engaging a few veterinarians and para-veterinarians to work alongside CVs to provide technical backstopping during vaccination campaigns. We further recommend close supervision of CVs, especially in the first few days of the campaign, to ensure shortfalls related to improper vaccine handling are eliminated immediately to safeguard vaccine potency. This should be followed by refresher training of CVs before each of the subsequent vaccination campaigns to enhance their performance.

## Supporting information

S1 Appendix A Training manual for smallholder chicken farmers. (PDF)

S2 Appendix Newcastle disease community vaccinators training manual. (PDF)

S3 Appendix A training guide for vaccine attendants. (PDF)

S4 Appendix Data collection forms for community vaccinators. (PDF)

S5 Appendix An evaluation form for community vaccinators. (PDF)

S6 Appendix Assessment form for performance and profiles of community vaccinators. (PDF)

S7 Appendix Appraisal and satisfaction assessment form for farmers. (PDF)

S8 Appendix A complete CONSORT figure showing drop out over time under paid and free vaccination frameworks. (PDF)

S9 Appendix Questionnaire used in assessment of intervention effect. (PDF)
