# Supplementary material for: Effectiveness of a community-centered Newcastle disease vaccine delivery model under paid and free vaccination frameworks in southeastern Kenya
Source: PLoS One. 2024 Aug 1;19(8):e0308088. doi: 10.1371/journal.pone.0308088 (PMC11293705; doi:10.1371/journal.pone.0308088)
Supplement: S1 Appendix — (PDF) [file pone.0308088.s001.pdf]

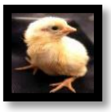

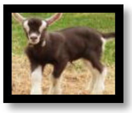

Chicken and Goat Husbandry Manual

The University of Nairobi

Grant No. 109063-001

The Cooperative University of Kenya

Grant No. 109063-002

Kenya Agricultural & Livestock Research Organization

Grant No. 109063-003

TRAINING MANUAL FOR SMALLHOLDER CHICKEN AND GOAT FARMERS IN MAKUENI COUNTY

One step at a time

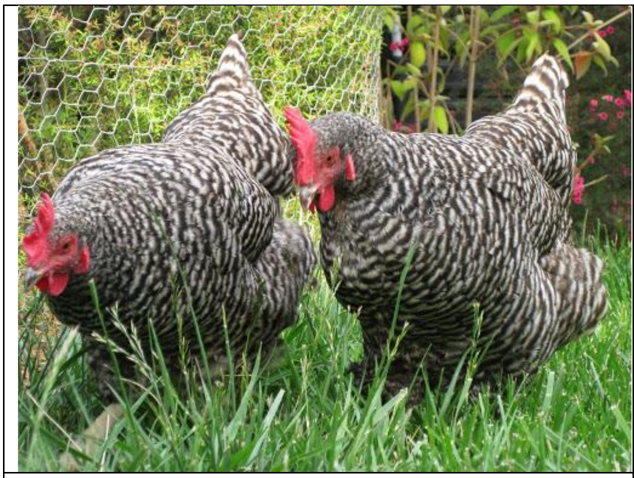

From two chickens

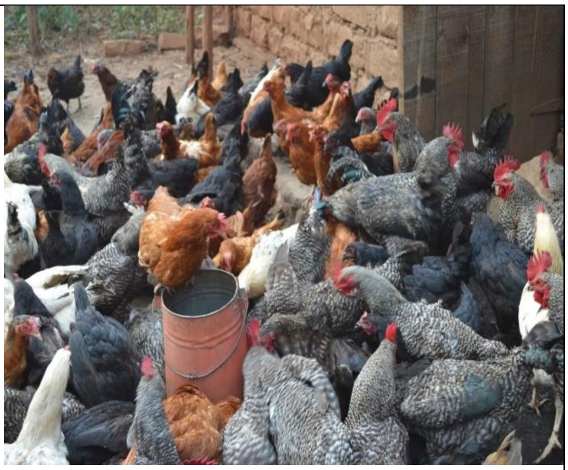

To many

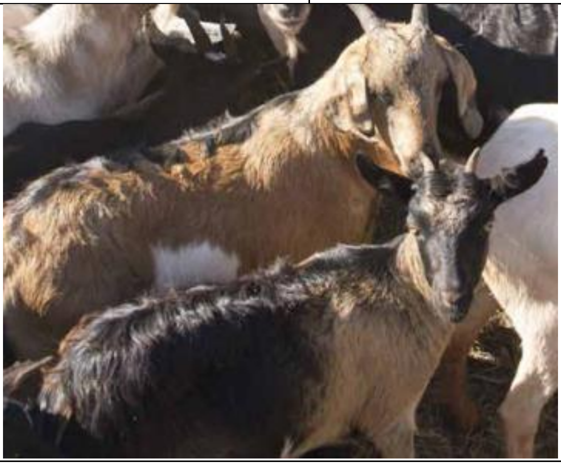

To finally acquiring goats

PURPOSE

To furnish smallholder chicken and goat farmers in Makueni County with knowledge and practical skills to enable them improve their chicken and goat production through proper control of Newcastle disease and contagious caprine pleuropneumonia (CCPP). We hope this will have the ripple effect of empowering women farmers through improved nutrition (chicken meat and eggs) at the household level and availing disposable income to women (managers of chickens).

OBJECTIVE(S)

To train small-scale chicken and goat farmers on best practices of raising chickens and goats for their eggs, meat and as an income generating activity. The farmers will be taken through general husbandry practices, housing design, Newcastle disease and other common chicken diseases, CCPP and other important goat diseases, disease control and prevention among other topics to equip them with basic knowledge needed to manage profitable chicken and goat production enterprises under prevailing conditions in Makueni County

ABOUT THE MANUAL

This manual is for use in training smallholder chicken and goat farmers. It is intended to furnish the farmers with technical knowledge needed to start and run a profitable chicken and goat enterprises in Makueni County. Because there are different levels of farmers in the county (some at advanced commercial stage while others still at subsistence level), the current manual is designed to cater for all the cadres but with a focus on smallholder chicken producers who are the majority in the county. The farmers will be trained and guided by experts on important aspects of chicken production using verbal lectures, visual demonstrations, illustrations, hands-on practical work and will be given a farmers' information handbook that contains diagrams, photographs and illustrations for future reference. This will help them in designing chicken housing and making quick diagnosis of common poultry diseases in the area and thereby initiating appropriate action.

CONTENTS

PURPOSE 2

OBJECTIVE(S) 2

ABOUT THE MANUAL 2

MODULE 1: Introduction and chicken breeds 6

1.1 Introduction 7

2.1 Session Learning outcomes 6

2.2 Time required 6

2.3 Materials/Planning needed 6

2.4 Training method 6

1.3 Chicken breeds 8

Evaluation Activity: 9

MODULE 2: Production systems and husbandry practices 10

2.5 Introduction 10

2.5.1 Session learning outcomes **10**

2.5.2 Time required **10**

2.5.3 Materials/Planning needed **10**

2.5.4 Training method(s) **10**

2.6 Chicken Production Systems in Kenya 11

2.6.1 Free range production system (for indigenous chickens) **11**

2.6.2 Semi-intensive chicken production system (indigenous and improved kienyeji) **12**

2.6.3 Intensive production system for broilers and layers **13**

2.3 **Error! Bookmark not defined.**

Improving survival rates of chicks (Brooding) 14

2.3.1 Natural brooding **14**

2.3.2 Artificial brooding **14**

2.5 How to collect, store and select eggs for hatching **15**

2.6 Hatching many chicks at once **15**

2.7 How to breed and manage cocks **16**

2.8 **Error! Bookmark not defined.**

Housing/structure for the chickens 17

2.8.1. Introduction **17**

2.8.2. Session learning outcomes **17**

2.8.3 Time required **17**

2.8.4 Materials/Planning needed **17**

2.8.5 Training method(s) **17**

2.8 Why construct a chicken house? 18
2.9 Requirements to observe when constructing a chicken house 18
2.10 Equipment used in chicken farming 19
2.10.1 Feeders 19
2.10.2 Drinkers 21
2.10.3 Laying and broody nests 21
2.11 Feed and water medication 22
2.12 Nutrition and feeding chickens 23
2.13 General disease control and prevention methods 24
MODULE 3: Chicken in health and disease 25
3.1 Introduction 25
3.1.1 Session Learning outcomes 25
3.1.2 Time required 25
3.1.3 Materials/Planning needed 25
1.1 How healthy chickens appear 26
1.3 Common sources of infections in chickens 27
1.4 Newcastle disease 28
1.5 How Newcastle disease is spread (transmitted) 30
2.3 Treatment of Newcastle disease 31
2.4 Prevention of Newcastle disease 31
Activity 2: 31
MODULE 4: Vaccines and control of Newcastle disease 32
4.1 Introduction 32
4.1.1 Session learning outcomes 32
4.1.2 Time required 32
4.1.3 Materials/Planning needed 32
4.2 What are vaccines? 33
4.3 Importance of vaccinating chicken 33
4.4 How to handle and store vaccines 34
4.5 Transporting vaccines 34
4.6 Giving Newcastle vaccine as eye drop or nasal drop 35
4.6.1 Directions for carrying out eye/nasal drop vaccination 35
4.7 Giving Newcastle vaccine in drinking water 37
4.7.1 Direction for drinking water administration 37
4.7.2 Newcastle vaccines in the market 38
4.8 Common causes of Newcastle vaccine failure 39

4.9 Monitoring efficiency of Newcastle vaccination program 39
4.10 Record keeping 40
9.0 Other important chicken diseases 41
MODULE 5: Goat husbandry practices 48
5.1 Introduction 48
5.1.1 Session learning outcomes 48
5.1.2 Time required 48
5.1.3 Materials/Planning needed 48
5.1.4 Training method(s) 48
5.2 Why keep goats: 49
5.3 Goat breeds 49
5.4 How to monitor performance of indigenous goats 53
5.5 How to handle goats 54
5.6 Feeding and stress 55
5.6.1 Causes of stress that should be taken into consideration 55
5.7 Housing structures 56
5.8 How to identify a healthy and sick goat 57
5.9 Contagious caprine pleuropneumonia (CCPP) 58
5.10 Treatment: contact a veterinarian to administer: 58
5.11 Prevention 58
5.12 11.1 How to keep your flock healthy 59
5.13 Other Important diseases of indigenous goats 60
5.14 11.4 Record keeping 62
Appendix 1: Ration formulation from locally available materials 63
Appendix 2: Vaccine regimes for hybrid chickens but also applicable to kienyeji chickens 64

MODULE 1: Introduction and chicken breeds

This module serves as an introduction to the different chicken breeds kept in Kenya. It further provides reasons why farmers keep chickens.

1.1 Session Learning outcomes

By the end of this session, the farmer will be able to:

I. Explain the reasons why people keep chicken
II. Distinguish the different breeds of chicken kept in Kenya
III. Differentiate between the local and the improved/hybrid chickens

1.2 Time required

This session should take one hour.

1.3 Materials/Planning needed

Slides, Charts, photographs

1.4 Training method

Lecture method, group discussions, demonstrations

Outline of the training interventions

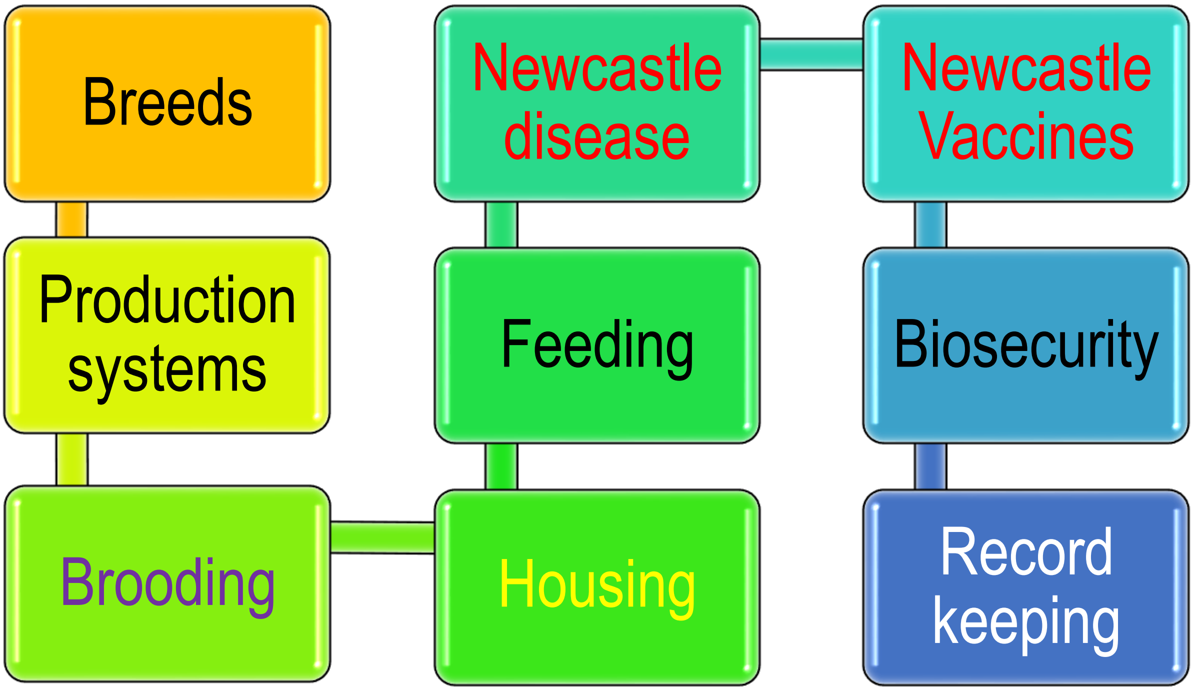

1.5 Introduction

- Chicken production has a potential to provide nutrition and generate income at the household level
- However, disease especially Newcastle disease hinder optimum production of chicken

1.6 Why keep chickens?

Chickens are kept for various reasons including:

1. Source of meat and eggs
2. As a quick source of income (money)
3. Create employment
4. Chicken droppings are good organic fertilizers
5. Feathers: are good for stuffing pillows, mattresses etc.
6. Economical to keep as it requires low initial capital investment, small land and low labour input
7. Used in traditional ceremonies, traditional human medicine, religious and sacrificial ceremonies, or as gifts
8. Their product is acceptable by most of the community

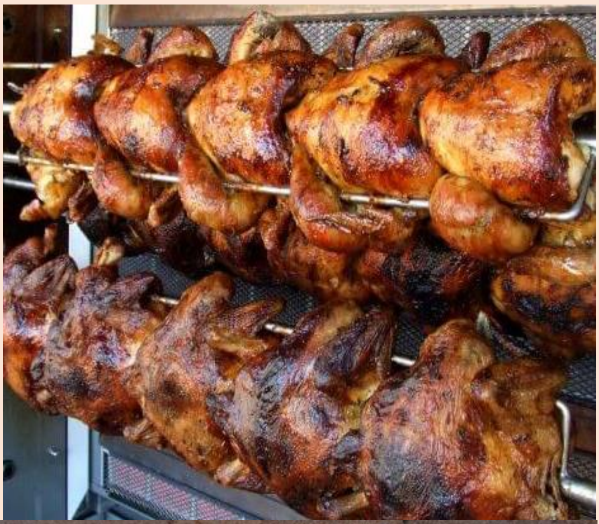

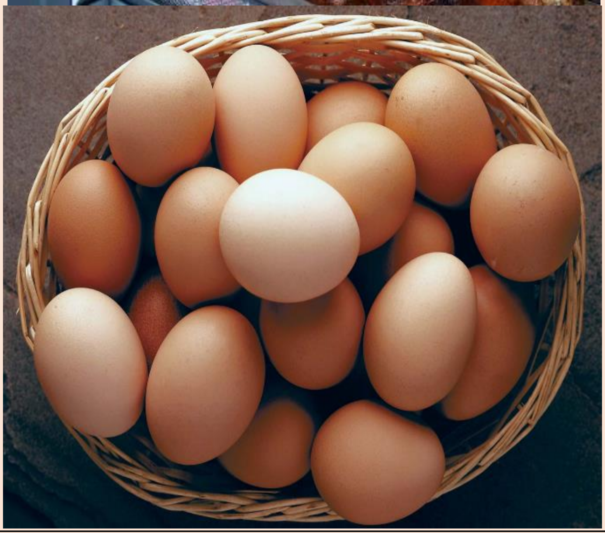

N/B: People in urban areas often consume more eggs and chicken meat than those living in rural areas, although rural people need these foods most. As such, it is so important to improve chicken production in remote areas of the country to provide easy access to chicken eggs and meat.

1.7 Chicken breeds

❖ What are the different types of chicken that you know or those found in your locality?

The following chicken breeds are currently kept in Kenya:

1. Local/indigenous chicken
2. Improved local/indigenous chicken
3. Hybrid chicken

a) Local indigenous chicken

This is the most common type of chicken kept by many smallholder farmers. They are well adapted to local conditions, look for their own food, and are more resistance to diseases. They vary a lot in size, colours and appearance. They grow more slowly, lay less eggs (about 60 eggs in a year) and do not add a lot of weight compared to commercial breeds even when they fed on commercial feeds.

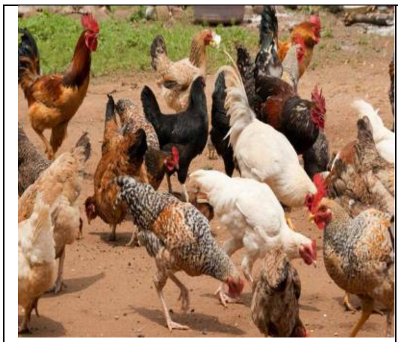	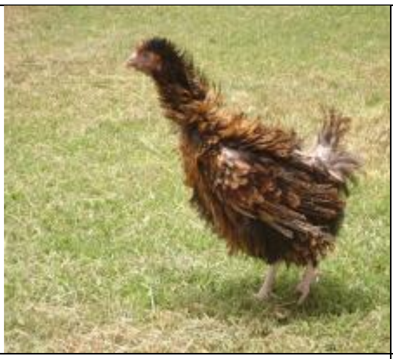	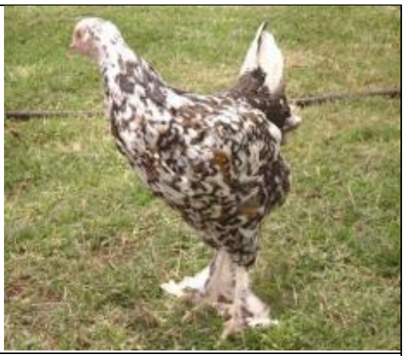
**Normal feathered kienyeji**	**Frizzled feathered chicken**	**Feathered shank hen**

b) Improved local/indigenous chicken

These are chicken that have been selected from among local chicken based on their ability to grow faster or lay more eggs than others. The chickens are then reared in a way that makes these characteristics even better. The improved local chicken varies in size, colour and appearance. They can only be distinguished from the unimproved local indigenous chicken from their growth characteristics and ability to lay eggs. Examples are KALRO Kienyeji, Rainbow Rooster and Kuroiler

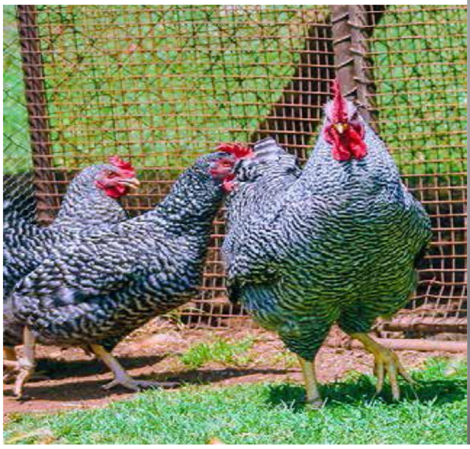
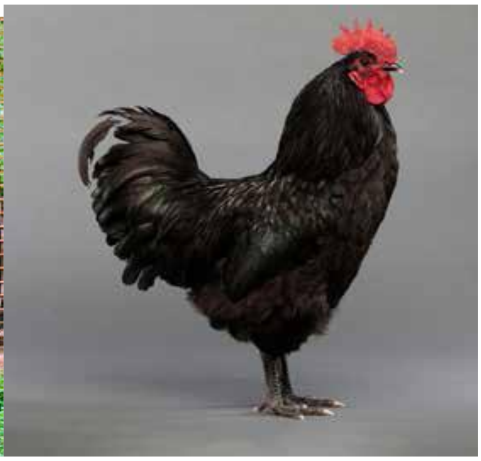

c) Hybrid/commercial chicken

These are chicken that were introduced in Kenya from European countries and are not well adapted to local conditions. They are kept in chicken houses and mostly feed on commercial feeds. They are two types – broilers and layers which are kept for meat and eggs respectively. They require more care than local chicken and are susceptible to diseases

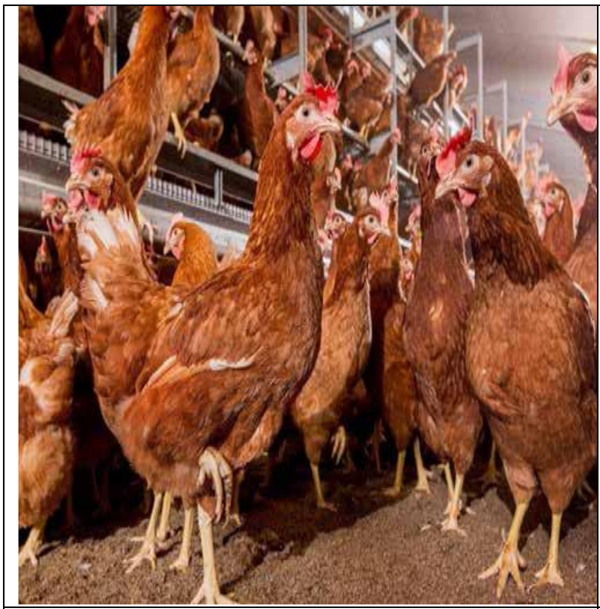	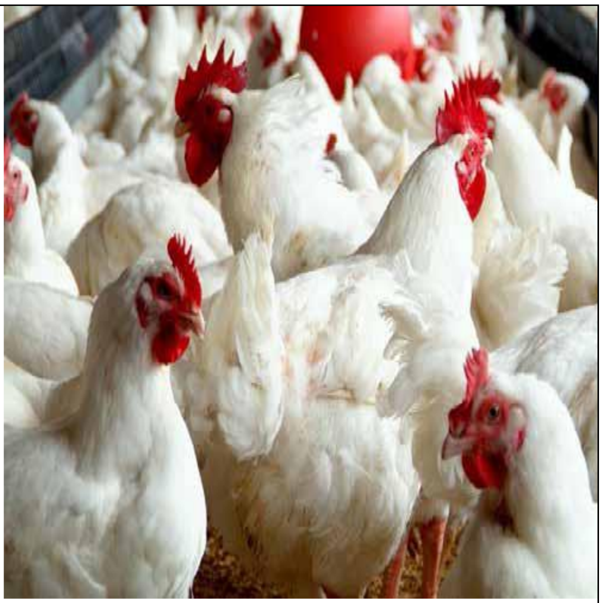
**Hybrid layers for eggs**	**Hybrid broilers for meat**

Evaluation Activity:

1) What kinds of chickens are kept in Makueni county?
2) What are some of the benefits of keeping chickens in Makueni County?

MODULE 2: Production systems and husbandry practices

2.1 Introduction

This session begins with an introduction to the three chicken production systems, namely free-range, semi-intensive and intensive production systems. The session delves into the local and artificial brooding systems used in improving survival rates of chicks. For sustainability, attention is also given to the process of collecting, storing and selecting eggs for hatching purposes apart from shedding light on the management of breeding of cocks.

2.1.1 Session learning outcomes

By the end of this session, chicken farmer will be able to:

I. Classify the different chicken production systems in Kenya.
II. Explain how the different production systems of chicken are operated in Kenya.
III. Distinguish between the different types of brooding in chicken.
IV. Explain how to collect, store and select eggs for hatching.
V.

2.1.2 Time required

This session should take one hour.

2.1.3 Materials/Planning needed

Slides, Charts, photographs

2.1.4 Training method(s)

Lecture method, group discussions, demonstrations

2.2 Chicken Production Systems in Kenya

Chickens are reared under three (3) production systems in the country: free-range, semi-intensive and intensive production systems.

2.1.1 Free range production system (for indigenous chickens)

- This is carried out mainly in rural areas mostly for household consumption and occasionally income generation
- Farmers keep small flocks ranging from (1-50), commonly 5-15 indigenous chickens
- The chickens are housed only in the evening either in the main house (enclosed baskets) with the household members, kitchen or small-structured chicken houses
- Are allowed to scavenge outside for food during the day (legumes, grains, worms, insects among others)

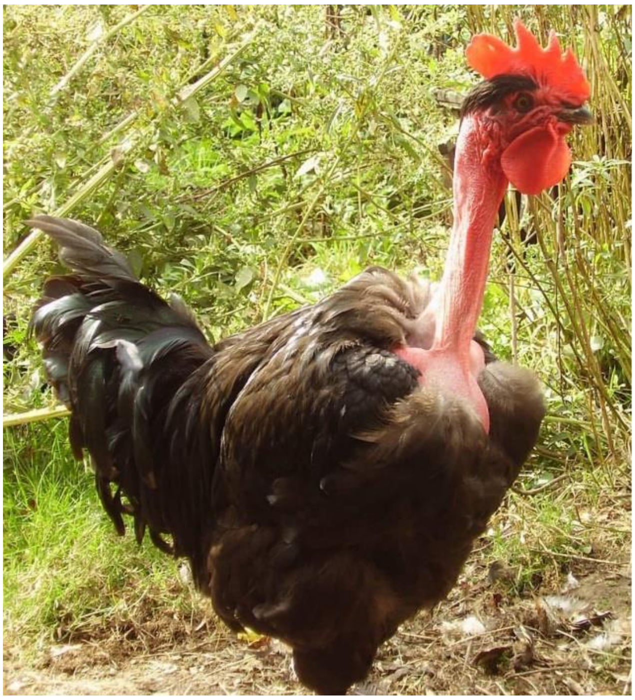

- Feeding is occasionally supplemented with locally available materials such as remnants of ugali, maize grain etc
- Little input is invested towards health services and hens incubate the eggs until they hatch
- High death rates arising from diseases such as **Newcastle** and predators
- Long broody periods
- Chickens are less productive (about 60 eggs/hen/year)
- Mainly use family labour and are cared for mostly by women and children
- Hens starts laying eggs at 24-30 weeks (6-7.5 months) of age
- Most chickens produce 2-4 clutches of eggs a year (10-12 eggs per clutch)

2.1.2 Semi-intensive chicken production system (indigenous and improved kienyeji)

- In this system, free ranging is supplemented with additional feeding, vaccinations, good hygiene and sanitation
- Require relatively high capital investment compared to extensive production system
- Flock sizes range mostly from 50 to more than 200
- Chickens have a small house where feeders, drinkers and laying nests are put, and where the chickens are housed at night
- There is a fenced area outside the chicken house where the chickens exercise, feed, drink, protected from predators and closely observed during the day
- The chicken house should be cleaned regularly and kept dry at all times
- Better health programs involving routine vaccination and strict biosecurity are usually put in place where profit is the main goal

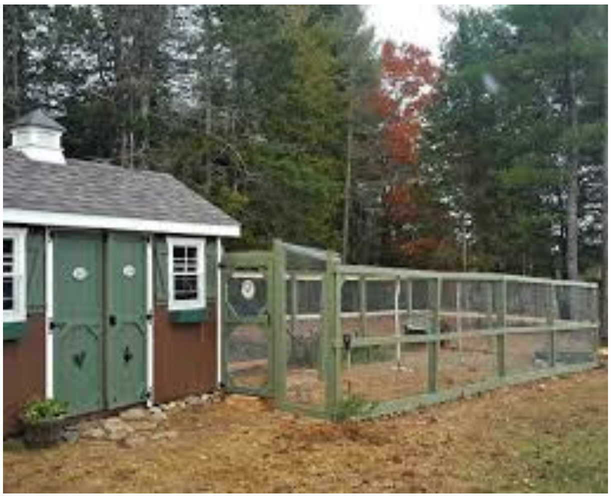

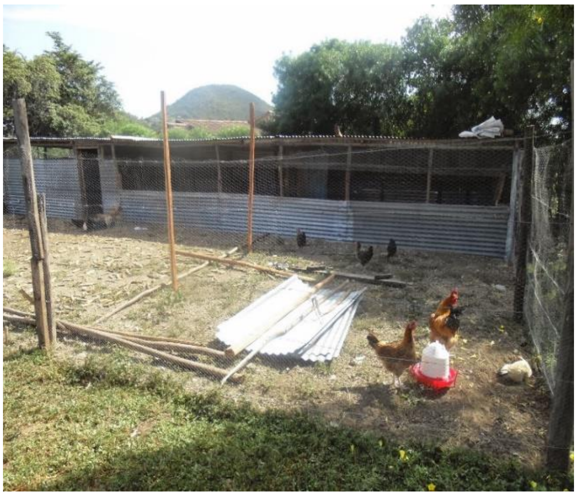

2.1.3 Intensive production system for broilers and layers

- This production system requires high initial capital investment (in breeds, feed, housing, health etc.) than the other two systems
- Flock sizes range from more than 200 to over 1000 chickens
- Main ones are deep litter (sawdust on the floor) and cage system.
- Used for large scale rearing of broilers and layers

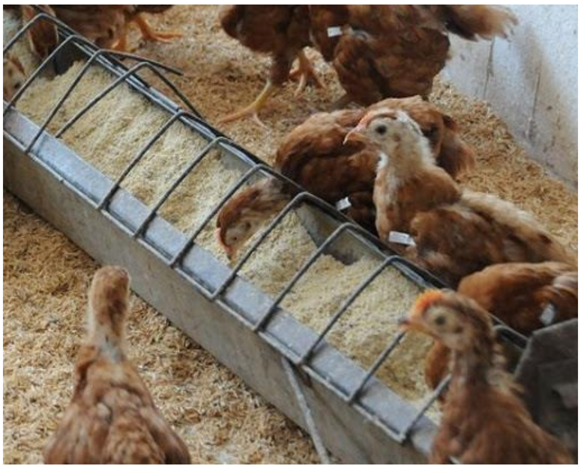

Deep litter for broilers and layers

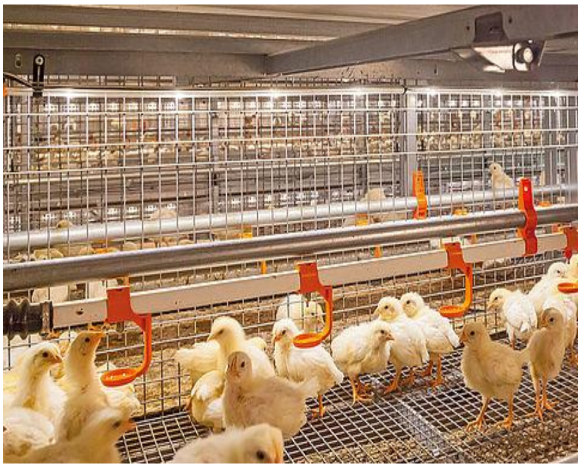

Cage system used mainly by layers

2.2 Improving survival rates of chicks (Brooding)

- Brooding is rearing of chicks until they are able to survive on their own
- Chicks should be protected from excess heat and cold, starvation, dehydration, predators and rodents
- Two types of brooding exist:

2.3.1 Natural brooding

- A broody hen rears the hatched chicks (providing heat and help them to get food)
- Put food and clean water near the chicks
- Protect the chicks from predators and rodents using improvised devices
- To reduce deaths, house the hen and chicks in the hut at night, put them in enclosures during the day with quality protein feed

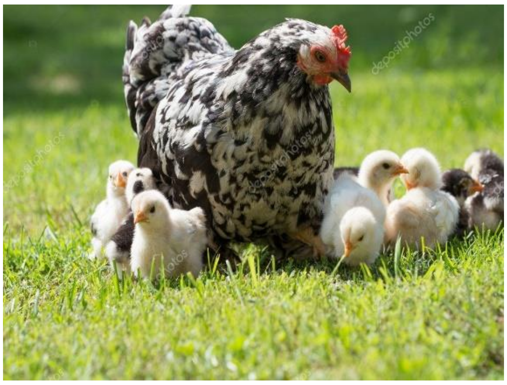

2.3.2 Artificial brooding

- Can be used to rear day-old chicks
- The following can be used to provide heat:

    1. Charcoal jiko: one is adequate for 500 chicks
    2. Kerosene lamp: one is adequate for 50 chicks
    3. Hay box or Baskets
    4. Electricity: one infra-red lamp for 250 chicks or 60-100 watt

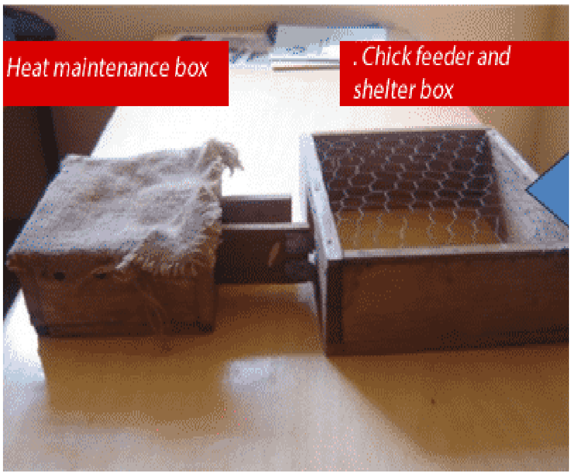

2.4 Monitoring temperature in the chicken house

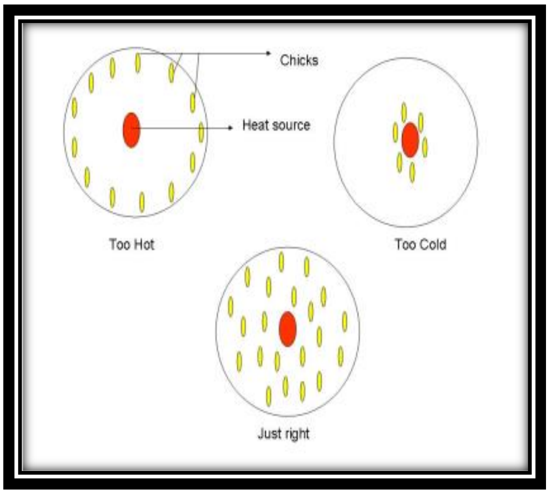

- Temperature above the floor should be kept at 32°C for the first week, and then lowered by 4°C for each week up to the 4th week
- Observe the reaction of chicks to the heat
- At correct brooding temperatures, chicks are evenly spread within the brooder ring
- At low temperature they crowd around the heat source
- At high temperature the chicks will move away from the heat source

2.3 How to collect, store and select eggs for hatching

- Remove the eggs once laid and stored them in a cool place
- When collecting eggs, do not frighten the chicken as a chicken being disturbed constantly may abandon the nest and not brood the remaining eggs
- Abandoned eggs can be given to other chickens to brood
- Provide good nutrition and clean water to increase the number of eggs laid per clutch (15-20)
- Eggs should be stored with the broad end facing upwards (contain air sac for breathing)
- Eggs older than 14 days (7 days for incubator machine) should not be used for hatching.
- Use a pencil to mark the eggs for ease of identification
- It is possible to check for freshness by placing the eggs in a container of water and seeing how they float. Fresher eggs float well, old eggs less well, while rotten eggs will not float at all

2.4 Hatching many chicks at once

Either broody hen or broody duck can be used to achieve this:

- Broody hen can be used to sit on up to 15 eggs continuously for at least 2 times by removing hatched chicks every time they hatch and replacing them with new eggs
- Ducks can sit on 30-35 eggs and can be used for up to 6 consecutive times
- Turkey can raise up to 50 chicks
- Hatching many chicks around the same time makes it easy to feed, vaccinate and sell
- When hens that start laying within the same week get broody, the first hen to become broody can be delayed by being given one (1) egg to sit on. This is repeated for second, third, fourth so that all hens are set on one day
- When ready to set, get rid of all the dummy eggs

2.5 How to breed and manage cocks

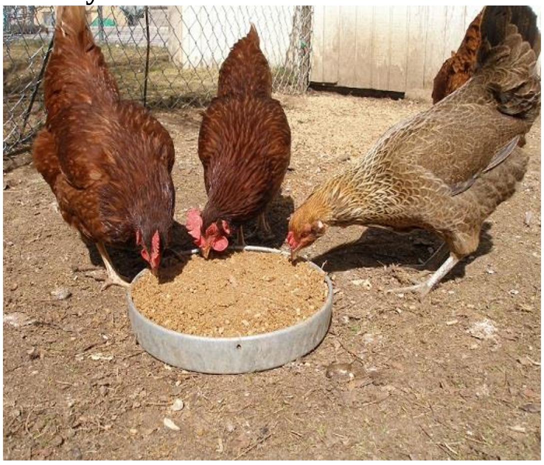

- One cock should be used to serve 10 hens
- Cock replaced after 2 years or less to avoid inbreeding
- Improved indigenous chickens can be used to improve indigenous chickens (kienyeji)
- Layers should be kept for about 2 years
- Hens can lay eggs for around 4-5 years before running out (chick-menopause state)
- Hybrid layers genetically bred for eggs usually run out of eggs earlier/faster (2 years)

2.6 Housing/structure for the chickens

2.6.1 Introduction

This session demonstrates the best practice in building a chicken house and the necessary equipment that must be available in the house for enhanced productivity of chickens. For the health of the chicken, the session explains the dos and don'ts in handling feed and water medication apart from discussing the nutrition guidelines of chicken. Finally, the farmers are provided with general disease control and prevention strategies.

2.6.2 Session learning outcomes

By the end of this session, the learner will be able to:

I. Explain why the chicken house is important to the farmer
II. List some of the requirements one needs to observe when constructing a chicken house
III. Identify the precautions farmers need to adhere to when administering feed through feed and water
IV. Highlight some of the general disease control and prevention methods in chicken farming.

2.6.3 Time required

This session should take 45 minutes.

2.6.4 Materials/Planning needed

Slides, Charts, photographs, objects (feeders, drinkers etc.)

2.6.5 Training method(s)

Lecture method, group discussions, demonstrations

2.7 Why construct a chicken house?

1. To protect chickens from extreme weather: rain, sun, cold and winds
2. To protect chickens from predators: dogs, cats, snakes, rats, other pests
3. To provide shelter for egg laying and broody hens
4. To minimise disease transmission and theft

2.8 Requirements to observe when constructing a chicken house

1. The roof should have a steep slope with overhang to allow rain water to run (rain proof)
2. The house should be at least 1.8-2.0m high to enable a person to stand upright
3. Should have proper lighting (natural or artificial light)

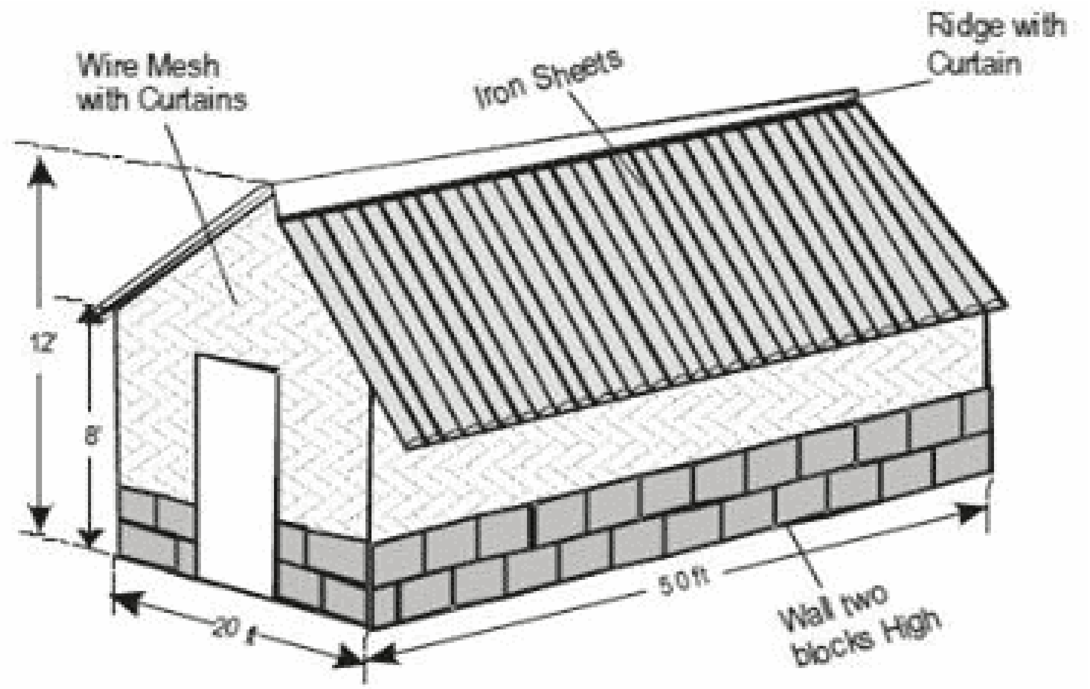

4. Should protect chickens from direct sunlight
5. In a rectangular house, the end walls should face East and West to ensure that only the end walls face the hot afternoon sun
6. Surfaces inside should me made from impervious material for ease of cleaning and disinfection
7. Wild bird proof
8. Have enough space to allow enough room for movement
9. Should have a good ventilation to allow fresh air circulation: get rid of excess ammonia and dust. Windows on either side. About 0.6m wire mesh at the back, 1m opening in front (2m in hot areas)
10. Prevent inflowing air from directly flowing on chickens
11. Should have solid door with a lock and any sharp object should be removed to avoid injury
12. The floor should be kept dry at all time. Stocking density (28-40 kg/m2)

2.9 Equipment used in chicken farming

The following equipment can be used in a chicken house to support productivity:

2.9.1 Feeders

- Are used to give food to the chickens
- Should be cleaned regularly to prevent spread of diseases
- Should be adequate for all chickens to feed at the same time
- One (1) metre trough or a 35 cm (diameter) tube feeder is big enough for 20 adult chickens to eat from
- Use feeders that do not waste food and limits contamination (cover with wire mesh)
- Do not be fill feeders to the top to avoid wastage (half-fills are better and then refilled periodically)
- Feeders can be made from locally available materials like wood, tin can, metal, plastic) in various shapes (long, round etc) or be bought
- Should be easy to clean, durable, strong, affordable and easy to fill
- Feeders should be put in easily accessible areas so that most of the droppings are concentrated in areas where they can be easily removed

Examples of commercial and homemade feeders

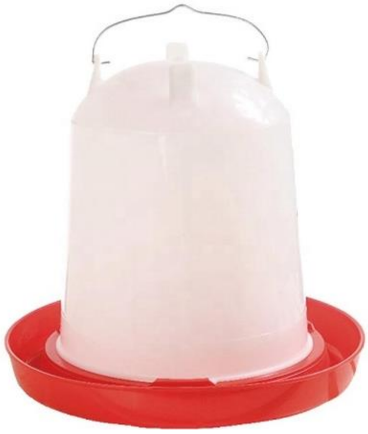
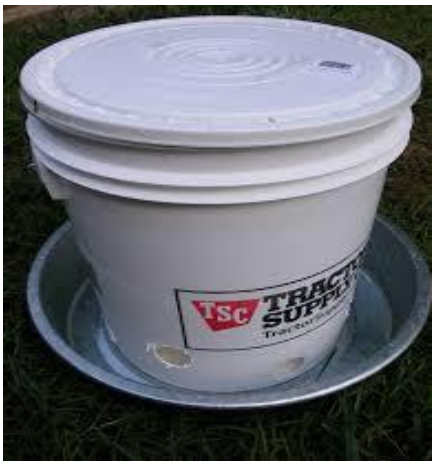

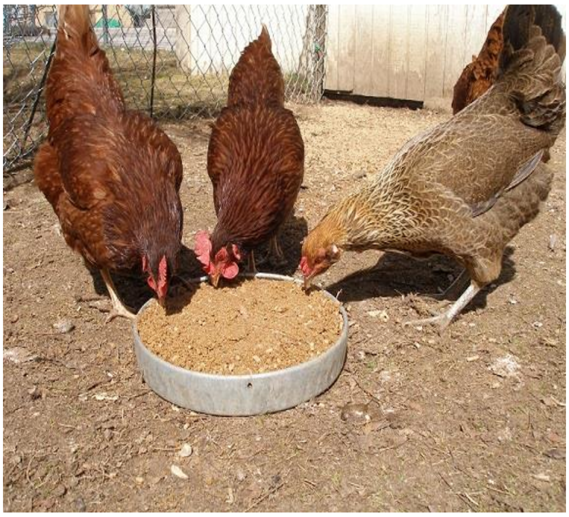
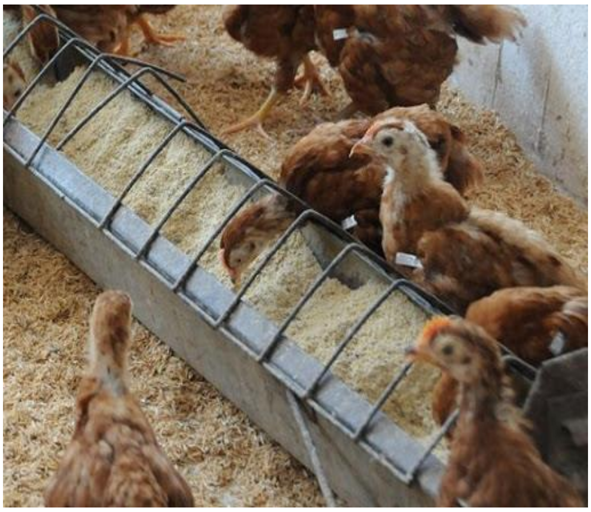

2.9.2 Drinkers

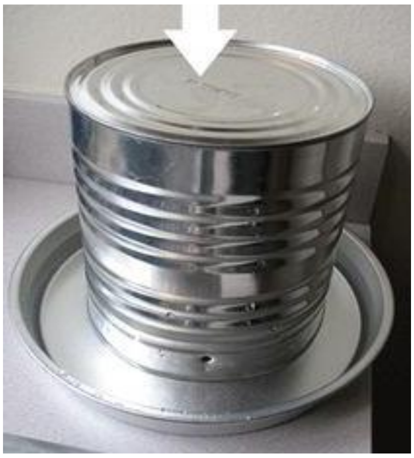

- Used to give drinking water to the chickens
- Should be cleaned regularly to prevent spread of diseases
- Drinkers should be cleaned and dried at least twice a day and sprayed with disinfectants once a week after thorough cleaning
- One metre trough or a 35 cm diameter tube drinker is big enough for 40 adult chickens to drink from
- Should be big enough for all chickens of the same age to drink from
- Can be made from locally available material at home or commercial ones bought (metals or plastics)
- Should be durable, strong, stable, easy to clean and fill
- Troughs, cups, hanging plastic containers or nipple drinkers can be used.

2.9.3 Laying and broody nests

- Safe and clean nests should be provided to laying hens to avoid laying in tall grass, ground or natural shelters
- Nest makes it easy to collect eggs and keeping them clean
- Very hot or too cold conditions can make plastic or tin nest sites too hot or too cold and kill the chicks
- Placed at least one (1) week before start of laying eggs
- Collect eggs twice a day at the same time every day in the morning and evening
- Removing eggs continuously is important if you don't want the hens to become broody
- Avoid building nests on the ground outside the chicken houses as eggs outside houses are more exposed to predators and thieves
- Nests should be placed inside the chicken house and preferably above the ground (1 for every 5 hens)
- Nests should be of the right size for the hen to feel comfortable
- Don't give food while the hen is in the nest
- The first eggs should not be collected or used for hatching
- A nest box measuring 30 x 30 x 30 cm is enough for 5 layers. Place in a wind free area
- It is advisable to place the laying nests in the dark part of the house to prevent exposure to the sun
- A calabash or nest basket may measure 40 x 20 x 25 cm (upper diameter x height x lower diameter). A clay pot is made more or less the same as calabash
- Any nest material should be removed and burnt after every brood to kill parasites and get rid of diseases especially those made of grass and wood shavings
- Examples of laying and brooding nest:

Examples of Laying and Brooding nests

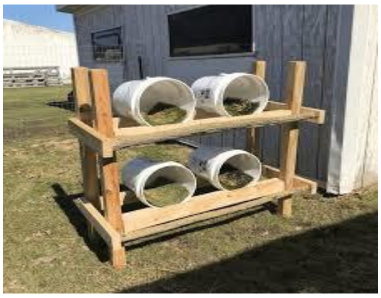
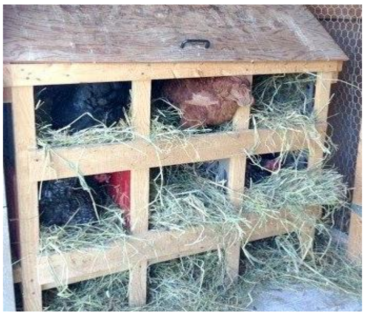
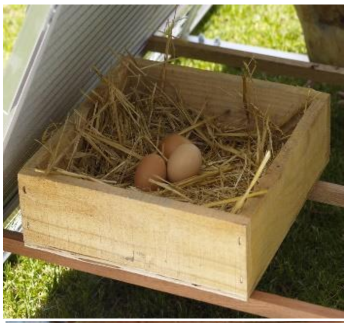
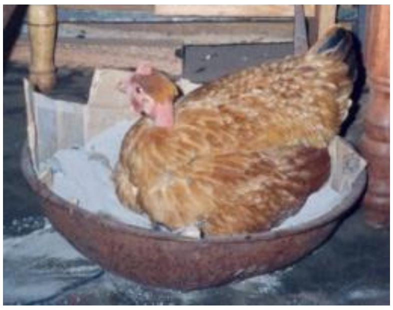
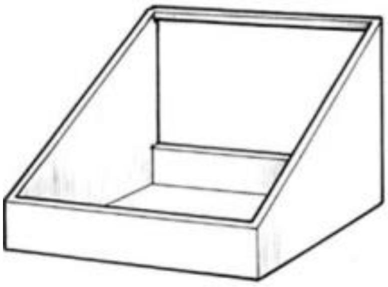
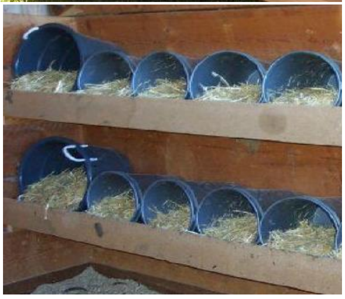

2.10 Feed and water medication

- Sick chickens are usually treated using drugs given in feed or drinking water
- This is given to all the chickens in the production unit (both sick and healthy)
- Mixing drugs with feed should be avoided as sick chickens always lose appetite and are too weak to compete for feed with healthy chickens
- Increase in total feed consumption due to other factors increases total intake of all feed ingredients, including drugs
- Water medication should be used since sick chickens would continue drinking water even when not eating
- Medicating all the chickens is useful in putting the disease in check until the chickens develop strong immunity to overcome infections
- Vaccines should only be given in non-treated (non-chlorinated or distilled) water, as chlorine or other sanitizing agents can destroy vaccines
- Avoid giving vaccine in water or feed for free-ranging chickens as it is not easy for chickens to consume the right dosage. Instead use eye/nostril drops
- Increased water intake during hot weather may lead to overconsumption of water medication, while cold weather may result in reduced water intake and thus under dosage of water medication
- If natural sources of water are available the intake by some chicken from the trough may be light
- Misuse of drugs mainly results from: miscalculation; or failure to consider feed and water intake, weather, failure to read instructions or follow directions and other variables.

2.11 Nutrition and feeding chickens

- Confined chickens need a properly constituted feed ration
- If feed is to be changed, it should be done gradually
- Give enough quantity of feeds to non-scavenging chickens in the same place every day
- Improved chicken breeds can only express their potential if they are provided with balanced ration
- Young chickens should be fed separately from the adults. They should have a special diet and it is best for them not to have to compete with the adults for food
- Laying hens should have easy access to calcium rich food that may be supplemented by calcium-rich sources such as crushed snail or egg shells (may eat eggs due to Ca deficiency)
- If you hatch too many chicks, they may die from starvation or malnutrition, or their resistance to diseases may be reduced
- Reduce flock sizes during dry periods
- A broody hen should be separated from the flock to prevent other hens from disturbing her. Keep the hen in a separate nest with free access to fresh water and feed within a short distance
- For scavenging chickens, supplementary feed and clean water should be given at least early in the morning and again in the evening when the chicken are returning to the house for the night
- Accidental omission of an ingredient, low-potency vitamin supplement, moldy or toxic contamination of an ingredient may result in a disease
- Feed consumption vary with weather (hot vs cold) changes; breed, type, strain, and age of bird; body weight; rate of lay; energy and fiber content of feed; and particle size of feed ingredients
- Store feed in a clean and dry area. For free-range, best scavenging time is early morning and late in the evening when insects are plenty and heat less
- Properly fed chickens have stronger immunity and are able to fight many diseases. Poor feeding results in reduced growth rate, reduced egg production and ease of getting infection

**Starter diet or (Chick Mash)**	**Growers' diet/mash**	**Layers diet/mash**
**High in protein (18%, 11.8 Mj ME)**; offered from day old up to 8 weeks; Each chick will consume about 2 kg during this period	**Lower in protein (14%, 11.6 Mj ME)**; offered from 9 weeks up to 18 weeks; Each grower will consume about 8 kg during this period	**Medium in protein (14%, 11.2 Mj ME)**; offered to hens from 19 to 75 weeks. Allow 120 g of feed per bird per day. Hens consume about 45 kg of feed annually
Chick mash from day 1 to day 56 (8th week), 60g of feed per day @50/= per kg of feed	Growers' mash from day 57 (9th week) to day 133 (19th week), fed 90g of feed per day @35/= per kg of feed	Provide both eggs and meat (more lucrative)

2.3 General disease control and prevention methods

In chicken production, emphasis should be on prevention of diseases rather than cure. To achieve this, the following management practices should be adopted by chicken farmers to prevent entry and/or spread of diseases in the farm:

1. During outbreak of Newcastle disease, vaccinate your **healthy** chickens against Newcastle disease
2. If litter is used, disinfect it before use and keep it dry throughout
3. Where possible, chicks should be separated from adult chickens because adults are usually carriers of diseases that can easily infect chickens
4. Separate sick chickens from healthy ones the moment you spot them and call a veterinarian or AHAs to help with disease identification and advice (ensure sick chickens are given food and clean drinking water), you can have a separate house or room for isolating sick chickens)
5. Do not sell or carry chickens to areas without the disease during outbreaks. It is better to slaughter sick chickens than wait for them to die
6. Immediately get rid of dead chickens by either burning or burying deeply
7. Never feed raw chickens that die from diseases to dogs as they can spread infections
8. Avoid introduction of new chickens from other places. Where new introduction cannot be avoided, separate (isolate/quarantine) the new chickens (purchased/gift) for at least 2-3 weeks
9. Limit contact between visitors and your chickens as they can transfer chicken diseases between farms
10. Avoid sharing your chicken production equipment such as drinkers and feeders with other farmers
11. Make sure the chickens have access to cool, clean drinking water at all times
12. Feed your chickens properly as this will build their immunity and help them fight common infections
13. Regularly clean chicken sleeping area in the house and equipment such as feeders and drinkers
14. Put measures to control rats and pests such as occasional use of dudu dust (sevin)
15. Spray the sleeping area for chickens and their equipment with antimicrobials/disinfectants such as iodine to get rid of infectious agents such as coccidia oocysts
16. Always keep the chicken sleeping area dry (air drying, sun drying)
17. Based on need, antimicrobials such as coccidiostats like amprolium can be given in food or water as preventive measures. **Consult a veterinarian before using any drug**
18. Maintain a routine vaccination program suitable for your level of production and potential risks
19. Sick chickens should be handled in ways that reduce spread of infection such as fed and their isolation areas cleaned last
20. Chicken production (even layers and broilers) improve if the chicken have access to outside exercise area, greens in the diet (deep yellow yolk), sunshine and areas where they can take dust baths
21. Avoid building brick houses as they are difficult to clean and locate chicken house at least 100m from other chicken houses
22. Allow adequate natural light (enough for a person to read a newspaper with) in the chicken house. Chicken require at least 8 continuous hours without artificial light at night

Evaluation Activity: In pairs the farmers can sketch/draw two sets of chicken houses: the one they have at home and the proposed ideal/modern chicken house

MODULE 3: Chicken in health and disease

3.1 Introduction

This session enlightens farmers on how they can detect sick chickens from healthy ones in order to initiate appropriate disease control measures. It goes further to highlight some of the common sources of infection in chicken. As one of the infections that poses danger to chicken, the session explains the cause, signs and symptoms, modes of spread, treatment and prevention of Newcastle disease. \

3.1.1 Session Learning outcomes

By the end of this session, the learner will be able to:

I. Identify the signs of healthy/unhealthy chicken.
II. Explain the common sources of infection in chicken
III. Describe the presentation of chicken which have been infected by the Newcastle disease virus.
IV. Discuss the modes of spread of Newcastle disease in chicken

3.1.2 Time required

This session should take one hour

3.1.3 Materials/Planning needed

Slides, charts, photographs

3.2 How healthy chickens appear

Newcastle disease vaccine should only be given to healthy chickens. Healthy chickens have the following characteristics:

- Externally they are clean, alert and on guard
- Have bright eyes
- Their comb and wattles are pink
- Have raised head and straight neck
- Are able to walk, stand, run and scratch
- Have strong legs and normal voice (crows)
- Have bright eyes and comb
- Continuously eat and drink
- Soft and compact droppings
- Breathe quietly
- Lay eggs normally
- Have smooth neat feathers
- Scales on the feet are smooth and yellow.

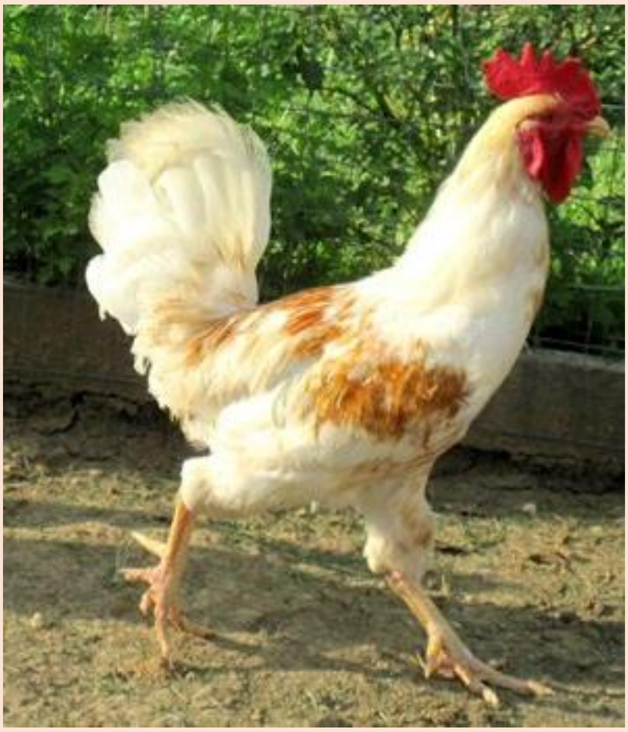

3.3 How unhealthy/sick chickens appear

1. Drop their head and close their eyes
2. Tired and lifeless
3. Dull eyes and comb
4. Sit or lie down
5. Eat and drink less
6. Lay less or stop laying eggs
7. Ruffled and loose feathers
8. Wet droppings with blood or worms
9. Diarrhoea, cough, sneeze and breathe noisily
10. Faeces smear around vent region
11. Unable to move and
12. Abnormal sounds
13. Legs bent and drooping wings

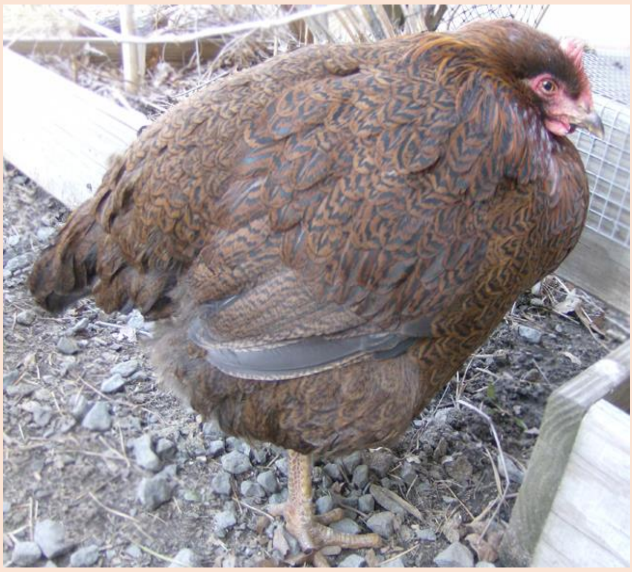

A sick chicken

3.4 Common sources of infections in chickens

Diseases can enter a farm from the following sources:

1. **Neighbours:** disease outbreaks in neighbouring homesteads can easily spread to your farm
2. **Human:** workers, customers and other visitors can bring infections to your farm
3. **Recovered carriers:** they appear healthy but they continue to shed germs causing diseases in faeces and discharges that can be a risk especially to chicks
4. **Housing chickens of different ages together:** due to carrier status, varying immunity among chickens of different ages
5. **Housing different chicken breeds together:** breeds that are resistant may transmit diseases
6. **New introduction:** new purchases, gifts etc. may bring diseases to the farm
7. **Egg-borne diseases:** these are passed from infected hen to new hatched chicks. Some diseases are carried inside the egg shell while others are just on the shell surface e.g. Salmonellosis
8. **Equipment:** sharing of contaminated equipment between farms and failure to clean utensils
9. **Others:** Rodents, household pets, wild birds, insects, feed

Activity: In groups of 5, the farmers can discuss based on experience, what causes infection in their chicken farms

3.5 Newcastle disease

- Newcastle disease kills more chickens than any other disease
- Farmers should be able to recognise this disease and know how to control and prevent it
- Newcastle disease occur mostly during dry seasons and affect chickens of all ages and sex
- The different strains of the virus causing Newcastle disease vary in strength
- The strong ones cause many deaths while those with low strength cause a few to no deaths.

For example, if a farmer has 10 chickens, and the Newcastle disease virus strikes then depending on the strength of the virus, it will cause deaths as follows:

Table 1: Strength of Newcastle disease virus and the number of deaths it causes

Description of Newcastle disease virus	Number of deaths it causes
A weak Newcastle disease virus	1 out of 10 chickens will die
A moderately strong Newcastle disease virus	5 out of 10 chickens will die
A very strong Newcastle disease virus	10 out of 10 chickens will die

i. Symptoms/Signs of a very strong Newcastle disease virus

- Many chickens die at once (50-100%), death within 3-5 days
- Infected chickens rarely show any clinical sign or symptom

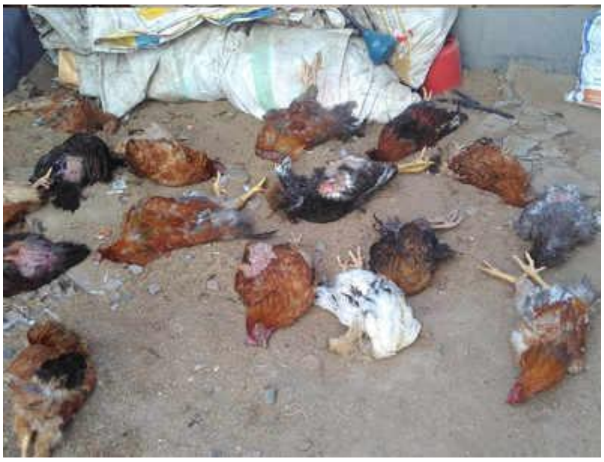

Many chickens die at once

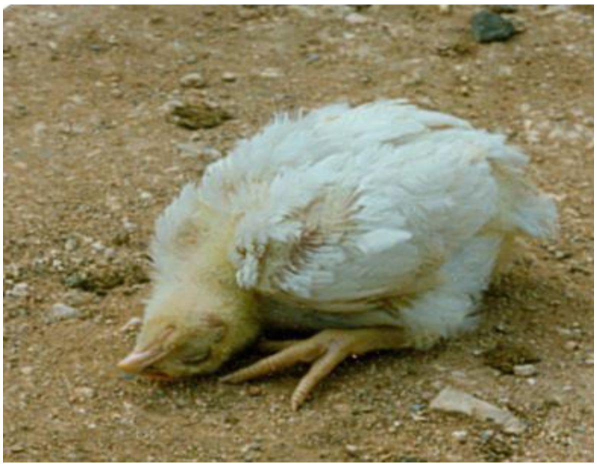

May present with twisted neck

ii. Clinical signs of a moderately strong Newcastle disease virus

Affected chickens may present with:

- Difficulty in breathing
- Discharges from the nose and mouth
- Weakness and dropping of wings and legs
- Twisted necks
- Drop in egg production 30-50% or more, return to normal in about 2-3 weeks
- Egg shell quality affected (thin, decolouration)
- Inactiveness, loss of appetite
- Swelling on face and neck
- Greenish and sometimes bloody diarrhoea
- Bleeding in the airways
- Bleeding throughout the intestine and bleeding in the proventriculus

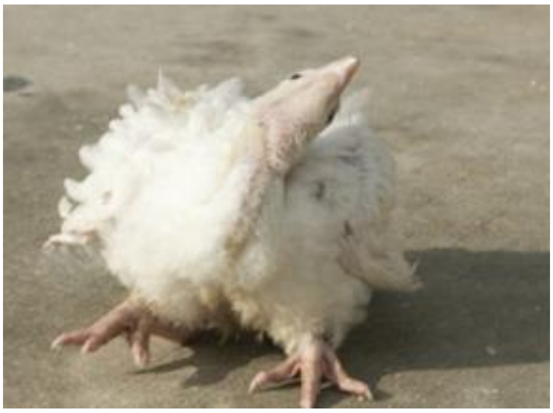

Twisted neck

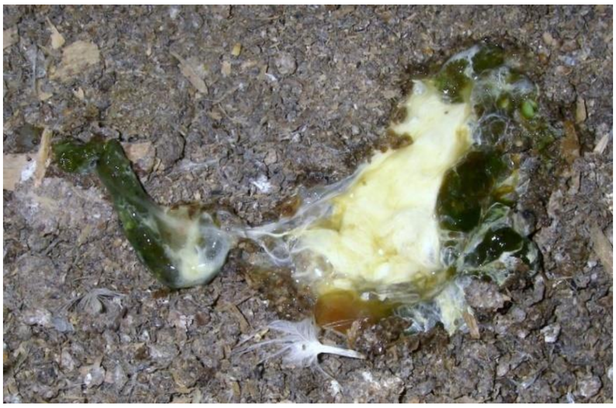

Greenish diarrhoea

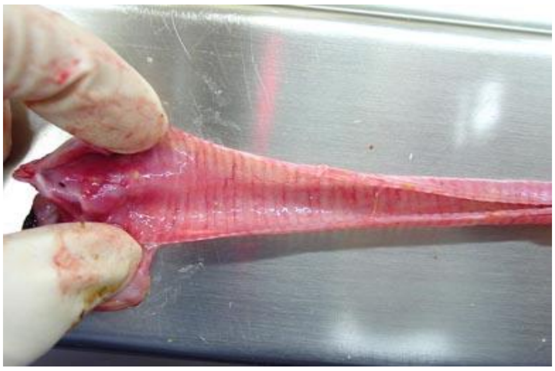

Bleeding in the airways

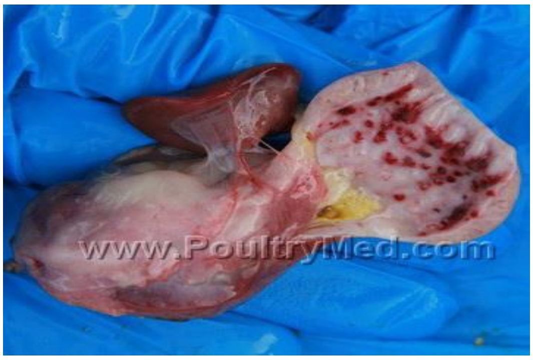

Bleeding in the proventriculus

3.6 How Newcastle disease is spread (transmitted)

- Newcastle disease is caused by a virus that is too small to see with naked eye
- When this virus enters the chicken's body, the chicken does not get sick immediately, instead the virus multiplies and increases inside the bird
- The chicken will become sick and show signs of disease after 3-5 days (but sometimes within 2-15 days)
- Newcastle disease is spread through:
  1. Breathing contaminated air
  2. Drinking contaminated water
  3. Eating contaminated feed
  4. Contact with sick chickens (new purchase, gifts)
  5. Contact with contaminated surfaces/products: meat, intestines, egg, feathers
  6. Contact with people (can be carried by shoes, clothes etc)
  7. Contact with parts of infected chickens (egg shell, feathers etc)
  8. Contaminated chicken house
  9. Contaminated cars, shoes, cages and baskets
  10. Carrier ducks, turkeys and some wild birds
  11. Carnivorous animals like dog and cats can help in the spread of the virus when they drag dead chickens
  12. Sharing of equipment of diseased chickens with healthy chickens

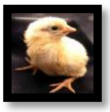

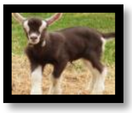

Figure illustrating different transmission pathways of Newcastle disease

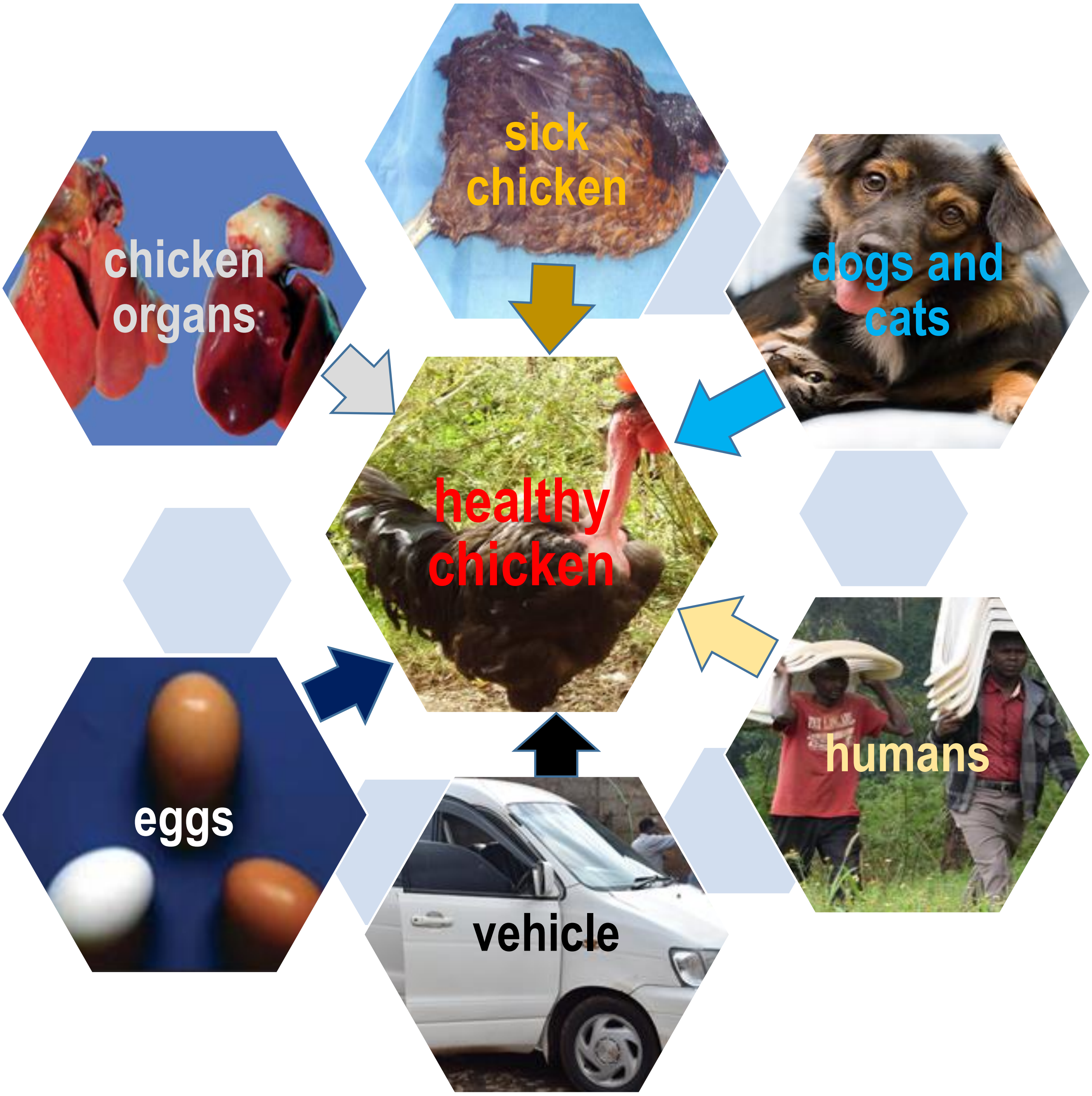

3.7 Treatment of Newcastle disease

Newcastle disease has **no** cure; chickens already showing symptoms/signs should not be **vaccinated.**

3.8 Prevention of Newcastle disease

The only effective means of preventing Newcastle disease is by protecting chickens from getting the disease through **vaccination.**

Activity 2: Find out if the farmers can explain what they do once their chicken have been infected by the Newcastle disease virus.

MODULE 4: Vaccines and control of Newcastle disease

4.1 Introduction

This session introduces farmers to the importance of using vaccine in the management of the Newcastle disease in the farms. In addition, it underscores the guidelines for safe handling, storage and transportation of vaccines. The farmers are taken through demonstrations on how to prepare and administer the Newcastle disease vaccine through the eye/nostril/water or food. Finally, the session highlights other diseases which affect chicken, their prevention and treatment.

4.1.1 Session learning outcomes

By the end of this session, the learner will be able to:

I. Explain the importance of vaccinating chicken in the farm

II. Outline some of the guidelines for handling and storing and transporting vaccines

III. Prepare the Newcastle disease vaccine for eye/nasal or water administration

IV. Determine the causes of Newcastle disease vaccine failure

V. Identify other infectious chicken diseases, their prevention and treatment.

4.1.2 Time required

This session should take one hour.

4.1.3 Materials/Planning needed

Slides, flip charts, photographs

4.2 What are vaccines?

- Vaccines are biological products that give protection to animals and humans against germs causing diseases
- They are used to **prevent**, and **not** to treat or cure diseases
- Vaccines are different from drugs such as antibiotics which are used to treat diseases
- A vaccine only protects against a specific disease it is given for e.g. Newcastle disease vaccine only protects against Newcastle disease
- For vaccines to work well, they must be stored, mixed, dosed, and applied correctly.

4.3 Importance of vaccinating chicken

1. To prevent chicken diseases such as Newcastle disease, fowl pox, fowl typhoid and other diseases
2. To prevent the spread of zoonotic diseases (such as salmonellosis) from chickens to humans
3. To prevent spread of diseases from the hen to chicks through the eggs.

NOTE: It takes about 7-14 days for chickens to develop protection against Newcastle disease after vaccination. In order to maximize protection against Newcastle disease, chickens should be vaccinated at least 4 times a year (after every 3 months) in places where the disease is prevalent.

4.4 How to handle and store vaccines

- Vaccines should be stored under refrigeration temperatures (4-8°C)
- **DO NOT** freeze or store vaccines in a freezer
- Vaccines should be protected from direct sunlight and heat
- I-2 Newcastle disease vaccine can be temporally stored at the base of a clay water pot kept in a clean dark place in rural areas
- Keep vaccines away from children (children should not play with vaccines).

4.5 Transporting vaccines

Chicken farmers going to buy vaccines from agro-veterinary outlets should transport them using:

1. Functional cool boxes with ice packs (for thermostable and non-thermostable vaccines)
2. A closed steel vacuum flask with ice in it (thermostable and non-thermostable) for short distances
3. Carrier containers carefully wrapped with damp cloth and protected from direct sunlight (thermostable vaccines).

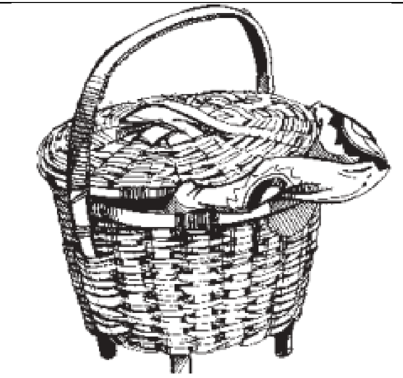

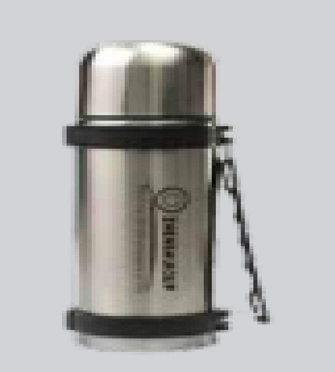

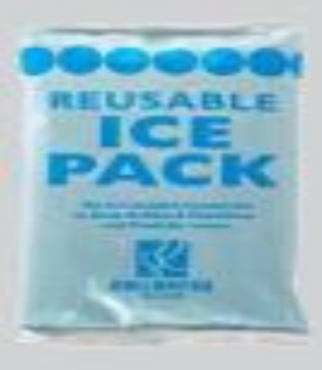

4.6 Giving Newcastle vaccine as eye drop or nasal drop

- To achieve a successful vaccination via eye/nasal drop, you require a hypodermic needle, syringe, or a dropper
- Eye drop vaccination produces stronger protection compared to other delivery routes. This is because of the lymphoid tissues located just behind the eyes where the vaccine goes to immediately after vaccination which produces stronger immunity

Advantages of eye/nasal drop: each chicken gets the vaccine, offers stronger protection, relatively cheaper as chickens are vaccinated three times per year.

4.6.1 Directions for carrying out eye/nasal drop vaccination

When carrying out eye/nasal drop vaccination the farmer should adhere to the following guidelines:

1. Ensure the vaccine to be given through eye/nasal drop is manufactured and clearly indicated for eye/nasal drop route
2. I-2 ND vaccine should be given by eye drop as this helps chickens develop stronger protection
3. Vaccines indicated for other routes must never be administered as eye/nasal drop to avoid complications
4. Open the vaccine vial and the diluent bottle (remove the aluminium seal and the rubber stopper)
5. When reconstituting the vaccine, the diluent temperature should be between (4-8°C)

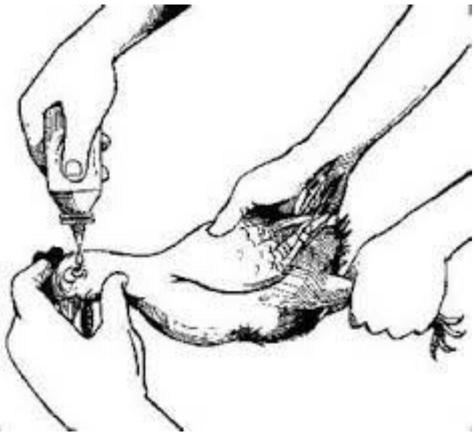

6. After firmly attaching the needle to the syringe, take the cap off the needle
7. Using the needle fixed to a syringe, withdraw some or all of diluent to be used to dilute the vaccine into the syringe through the needle
8. Inject into the vaccine vial in the middle of the rubber stopper and mix gently (if vacuum is still intact, it will pull some of the liquid into the vial without any pressure from the vaccinator)
9. Withdraw the mixed vaccine and transfer into the diluent vial and mix well (by moving from side to side). **Do not** shake violently to form bubbles
10. Examine each chicken to make sure it is healthy before you vaccinate it
11. Use a syringe or a dropper to apply one or two drop(s) in either eye or nostril as directed by the manufacturer

12. A vaccine will only be effective if the drop (0.03ml) is placed into the eye or nostril and absorbed. To achieve this, wait for about 10 seconds after administering the drop before releasing the chicken
13. If the drop is not completely absorbed, a new drop should be applied
14. To prevent the contents of the vial from getting warm against the hands of the vaccinator, divide the contents of the reconstituted vaccine into two or three empty vials and alternate their use while keeping the others in a cool box with ice packs
15. Vaccination should be done under a shade (of house or tree) away from direct sunlight

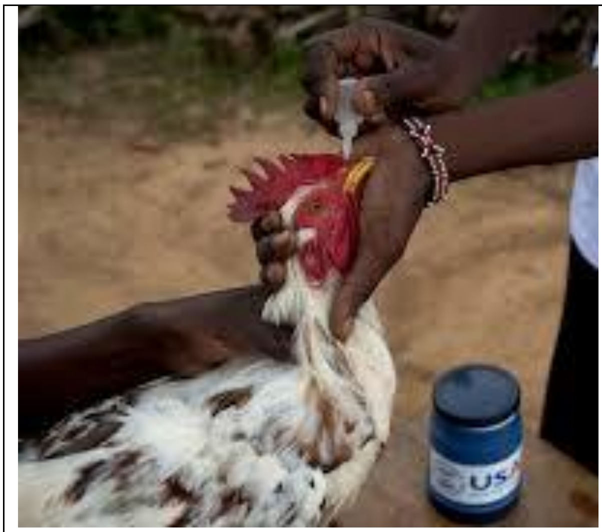	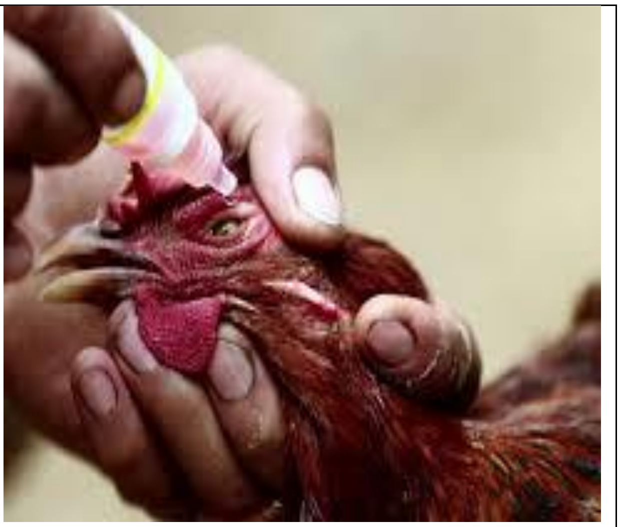
**How to place a nasal droplet**	**How to place an eye droplet**

Table 1: Comparison of different routes of giving thermostable i-2 Newcastle disease vaccine

Route of giving vaccine	No. of applications per year	Degree of protection
**Eye/nasal drop**	3	High (80%)
**Drinking water**	5	Moderate (60%)
**Food**	8-12	Low (50%)

4.7 Giving Newcastle vaccine in drinking water

Newcastle vaccine can also be given to the chickens in drinking water.

4.7.1 Direction for drinking water administration

When administering the Newcastle vaccine through drinking water, consider the following guidelines:

1. Do not open or mix the vaccine until ready to vaccinate
2. Remove all medication, sanitizers and disinfectants from the drinking water 48-72 hours prior to vaccination and up to 24 hours after vaccination (48 for antimicrobials)
3. Provide sufficient drinkers so that all the chickens can drink at one time. Clean and rinse all the waterers thoroughly before use
4. Withdraw water from the chickens for 1-2 hours prior to vaccination to stimulate thirst (30-60 minutes in hot climates; 60-90 minutes in cool climates)
5. Boil the water (NOT tap water) to be used in reconstitution and allow it to cool
6. Open the vaccine vial by removing the aluminum seal and rubber stopper
7. Using the water that will be used in reconstitution, fill vaccine vial two-thirds with clean, cool water and mix gently in order to reconstitute the vaccine
8. Rinse the vaccine vial three times to remove all the vaccine
9. Estimate the average daily water consumption then calculate 30% of the volume of water to prepare the vaccine
10. Addition of skim milk powder (at the rate of 500g/200L) to the water 20-30 minutes before adding the vaccine is recommended to stabilize the water
11. Mix with stock solution after adding the vaccine stabilizer following manufacturer's instructions
12. Distribute the vaccine solution to the waterers placed where there is no direct sunlight
13. Do not provide any other drinking water until all of the vaccine mixture has been consumed
14. Check to ensure all the birds are drinking water, if not, redistribute the drinkers as necessary
15. The chickens should drink all the vaccine solution within 1-2 hours (never in less than 1). When the vaccine is consumed too fast, other chickens will not have an opportunity to drink
16. Record all vaccine information as well as any challenges/problems which may occur with the chickens or vaccination process

4.7.2 Newcastle vaccines in the market

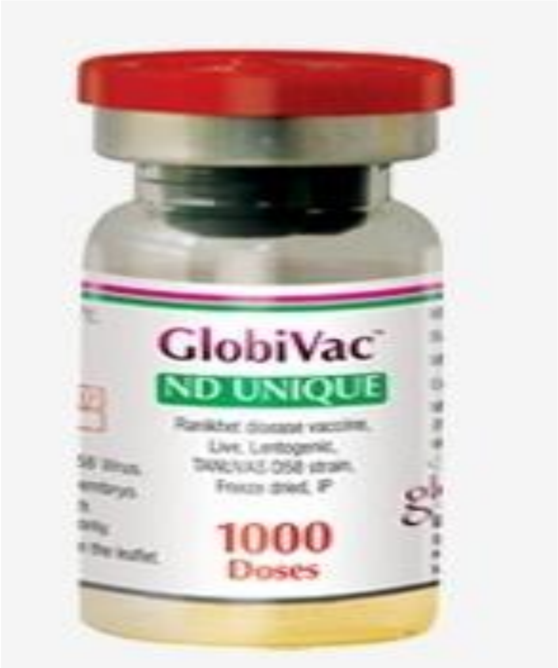

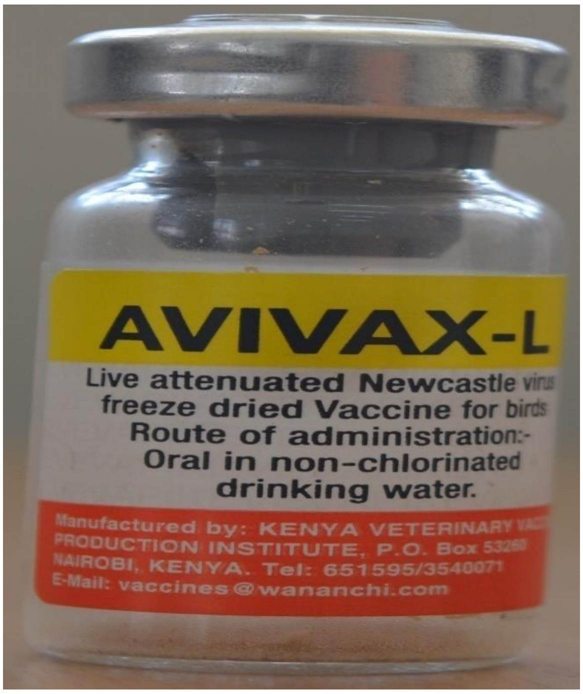

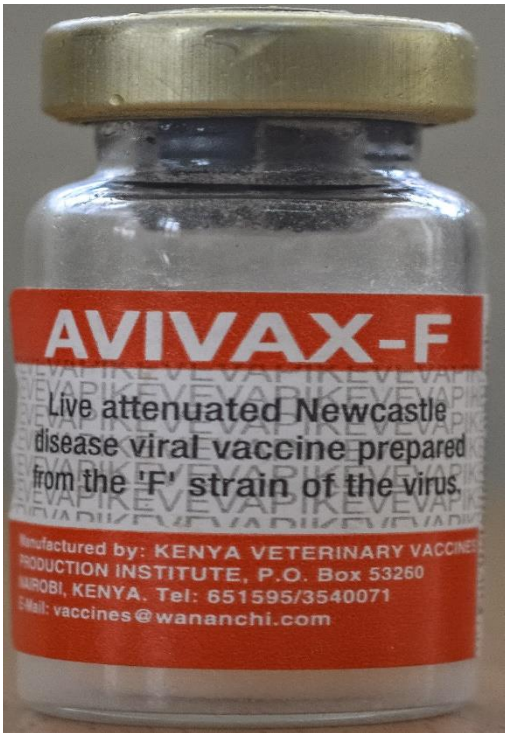

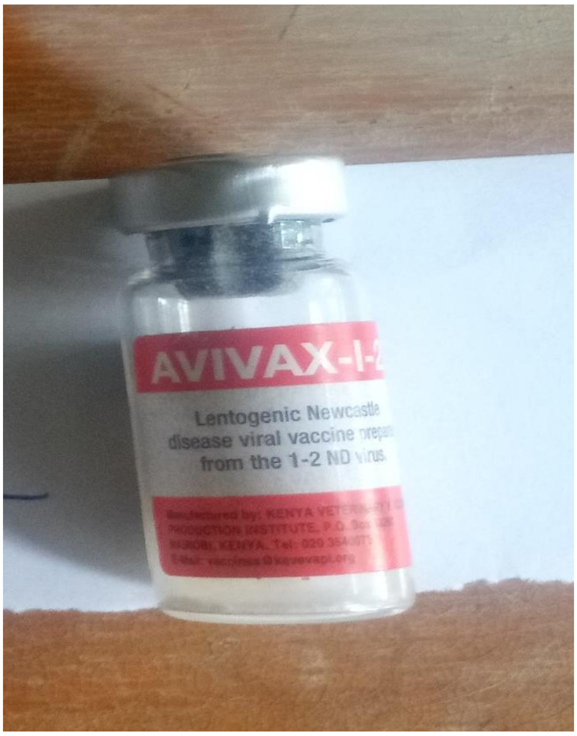

etc.

4.8 Common causes of Newcastle vaccine failure

The Newcastle vaccine may fail because of the following:

1. Giving Newcastle vaccines incorrectly (wrong route, poor reconstitution, wrong dosage)
2. Poor handling of live Newcastle vaccines (breakdown in cold chain)
3. Using tap water/chlorinated water to reconstitute Newcastle vaccines
4. In some cases, the field strain of an organism is very strong, and the vaccine strain is weak. In this situation, the flock may be effectively vaccinated, but the immunity is insufficient to protect against the disease completely
5. Failure to observe hygiene and sanitary conditions allows infectious disease agents build up, it is possible that the challenge dose of a particular infectious agent will be so great, or so soon, that a normally effective vaccination program will be overwhelmed
6. The immune status of the parent flock also can contribute to a vaccine failure. If the breeder flock provides progeny with high levels of maternal antibodies, vaccination during the first 2 weeks of life may result in the vaccine being neutralized
7. Certain infectious diseases that lower immunity may result in vaccine failure
8. Once reconstituted, vaccines should be used within 30-45 minutes, any that remain should be discarded.

4.9 Monitoring efficiency of Newcastle vaccination program

The following indicators can be used to monitor the effectiveness of vaccination programs adopted by farmers:

- Morbidity: absence or reduction in number of sick chickens is a sign of success of the program
- Mortality: absence or reduction in number of dying chickens is a sign of success of the program
- Increase in number of chickens.

Evaluation activity: Let the farmers discuss in groups the effectiveness or challenges of administering the Newcastle disease vaccine in either water, eye/nasal modes

4.10 Record keeping

All chicken farmers should learn to keep simple records (taken daily or weekly) on performance of their production. This will help in early identification of diseases, poor nutrition and other problems so that interventions can be initiated in good time. The following information should be taken by the farmers:

a. **Feed intake**: name of the farmer, age in weeks, light exposure (hours), chicken category based on age (chicks, grower, layer/hen, cock), feed intake (gm/day/head)
b. **Chicks and brooding:** chicks growth data, date of brooding, breed, age in days, number of chicks present, mortality data, average body weight (every 15 days), causes of death etc.
c. **Market:** number and chicken type sold (hen, cock, grower, chicken), eggs sold, where and to whom sold, eggs/chicken consumed and to whom, income from sale of eggs, gifts and home consumption of eggs and chickens by your family and friends should also be noted, all expenditures for feed or feed ingredients should be registered carefully, noting quantities, price and date of purchase. If you buy feed from feed sellers, note the name of the seller and the time of purchase
d. **Layers**: date of brooding/hatching, date of start of laying egg, breed, number of eggs, laying per day/head, number of eggs broken or out of use, number of hens (present, died, transferred), eggs laid, dead chickens, cause of death and chickens given as gifts
e. **Health record sheets:** date of vaccination, name of the vaccinator, type of vaccine, name of the disease, method of vaccination, etc.
f. **Treatment data**: date of treatment, age of the chicken (week), type of drug used, dose of the drug used, method of drug administration (water, feed), etc.

4.11 Other important chicken diseases

These chicken diseases are generally caused by:

**1. Viruses**	Have no treatment/cure. Antibiotics have no effect on viruses<br>Prevented by vaccination
**2. Bacteria**	Can be treated by antibiotics
**3. Protozoa**	Treated using antiprotozoal drugs
**4. Internal parasites**	Treated by giving chickens dewormers
**5. External parasites**	Treated using pesticides

Some of these diseases include the following:

1. Fowl pox

**Wet form**	**Dry form**
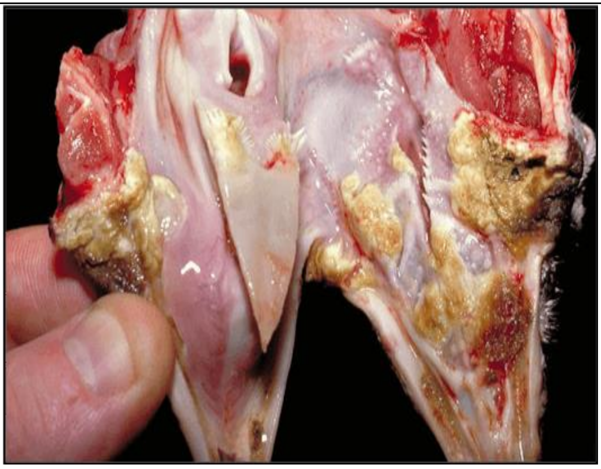	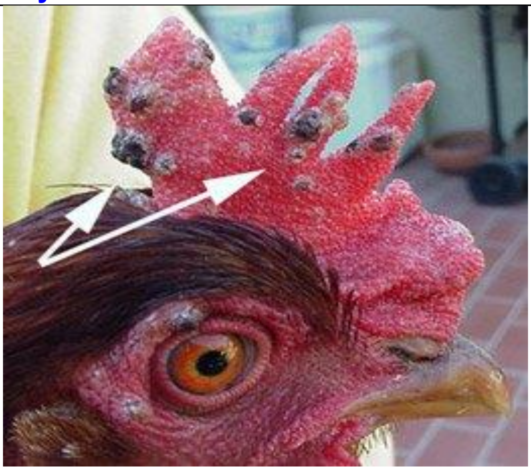
Swellings in the membranes of the mouth and airways	Dry swellings on the skin especially of the combs, face and wattles
**Treatment:** Application of kerosene/liquid paraffin on the lesions and use of antimicrobials administered in feed/water against secondary infection can hasten recovery	
**Prevention:** Vaccination by wing web stab at 4-6 weeks of age	

2. Marek's disease (MD)

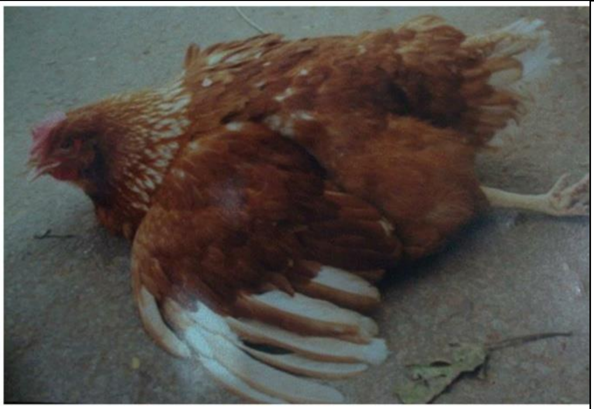	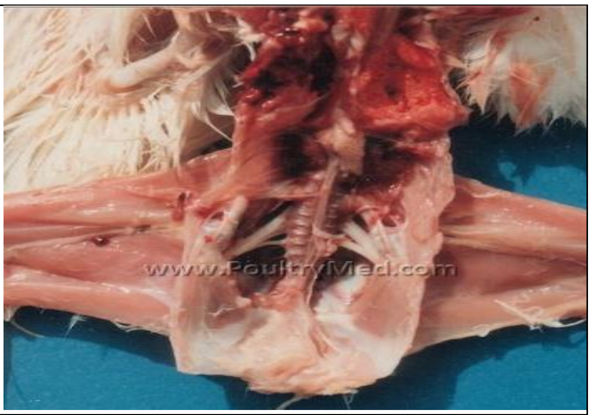
Loss of muscle function of the wings, legs are weak and extended	Swollen brachial plexus nerve
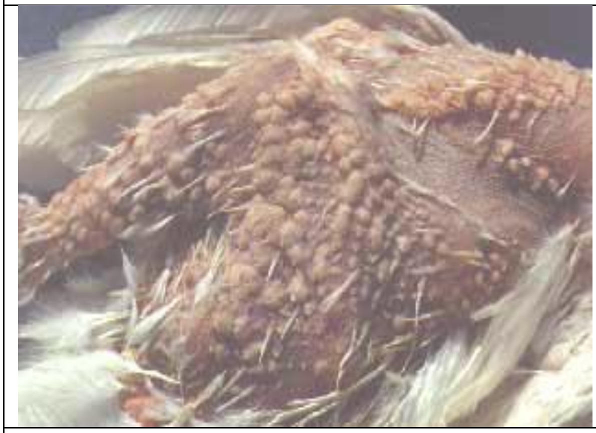	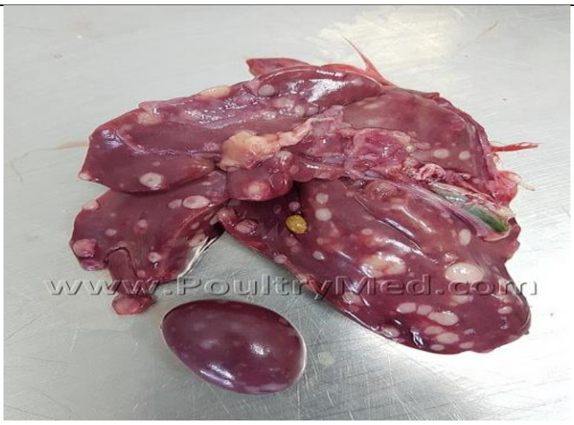
Abnormal growth of skin around the feather follicles	Enlarged liver and spleen with diffuse greyish swellings
**Prevention:** Vaccination on day 1 of hatching	

3. Gumboro disease (infectious bursal disease)

❖ Bleeding into skeletal muscles of the leg<br>❖ Enlarged bursa of Fabricius (lymphoid tissue)<br>❖ White watery diarrhoea<br>❖ **Prevention:** vaccination	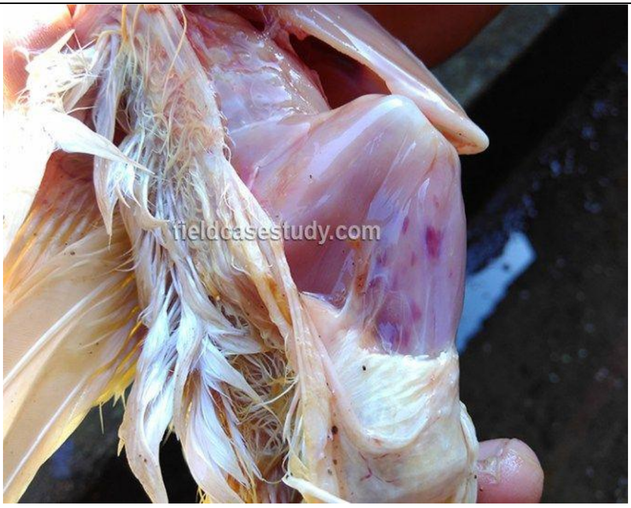

4. Infectious bronchitis (IB)

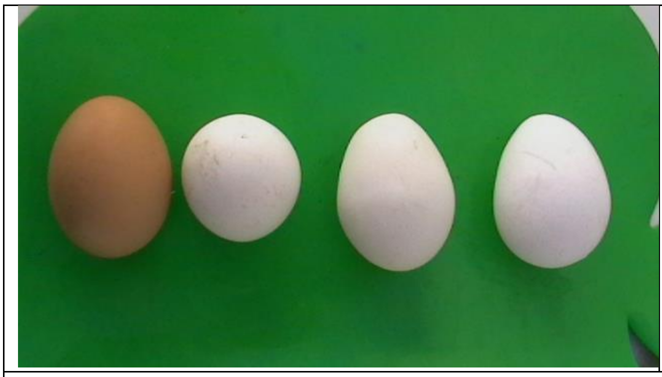

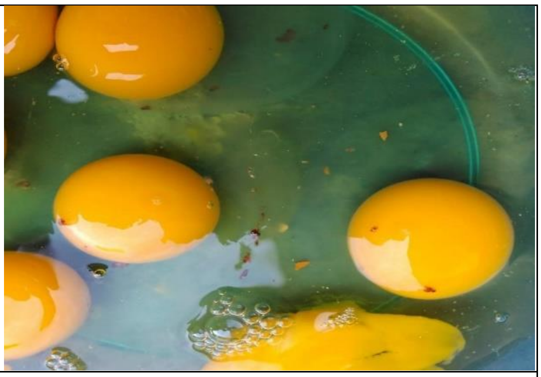

- ❖ Significant drop in egg production
- ❖ Increased number of poor-quality eggs - soft shells, misshapen, with watery content
- ❖ Difficulty breathing (open beak), irritation of airways
- ❖ **Treatment:** There is no treatment. Antimicrobials and vitamins given in water to prevent secondary infection and hasten recovery
- ❖ **Prevention:** Vaccination

5. Fowl cholera

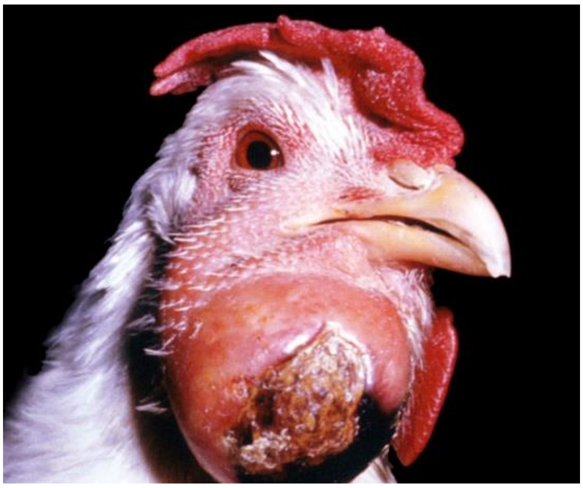	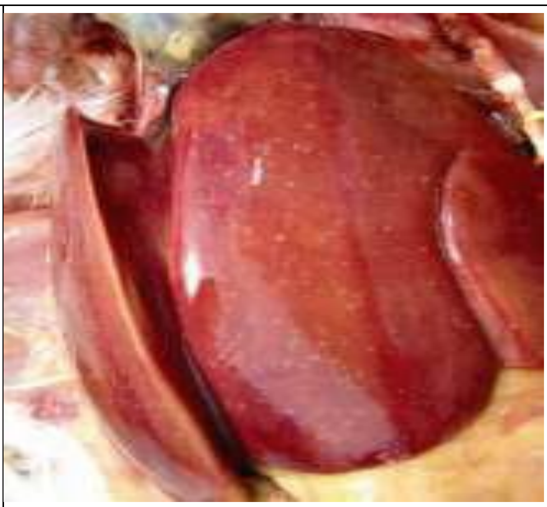
❖ Swollen wattle with yellow-brown pus<br>❖ Pus (whitish to yellow) accumulate in the joints	Pinpoint bleeding in the muscles of breast, heart
**Treatment**: antibacterial drugs effective against *Pasteurellamultocida* infection such as erythromycin, tylodoxycin, oxytetracycline	
**Prevention**: Vaccination	

6. Infectious coryza

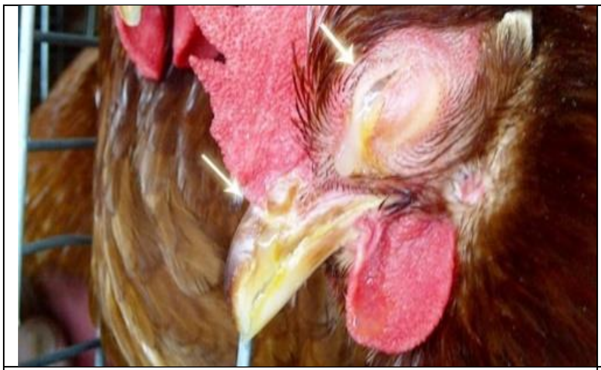	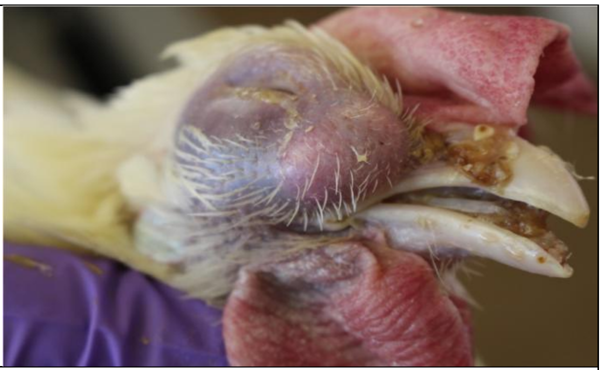
Discharges from mouth and nostril	❖ Swollen eyes and face<br>❖ Eyelids stick together by mucous
**Treatment**: Antibacterial such as erythromycin and tetracyclines in water to alleviate severity and course of the disease<br>**Prevention**: Separation of sick chickens to prevent spread.	

7. Coccidiosis

❖ Watery diarrhoea, sometimes with blood<br>❖ Present with small intestine distended with blood (twice its diameter)<br>❖ Bloody areas are clearly seen without opening the intestine<br>❖ Partially clotted blood in the intestine or mucoid contents	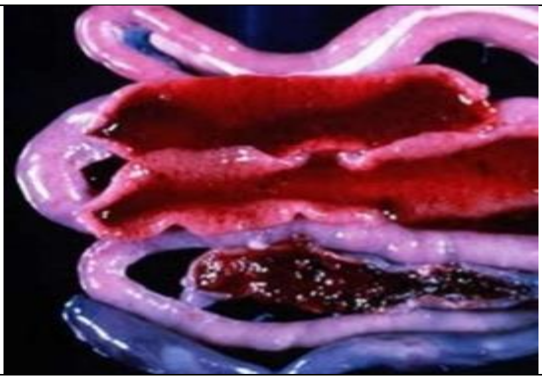

8. Worms (Minyoo)

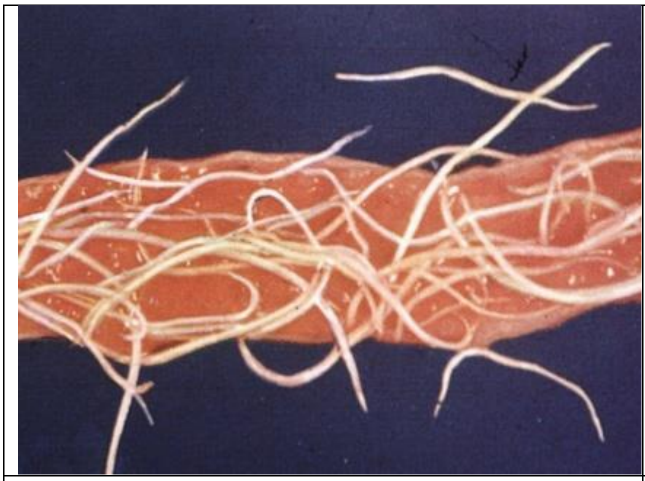	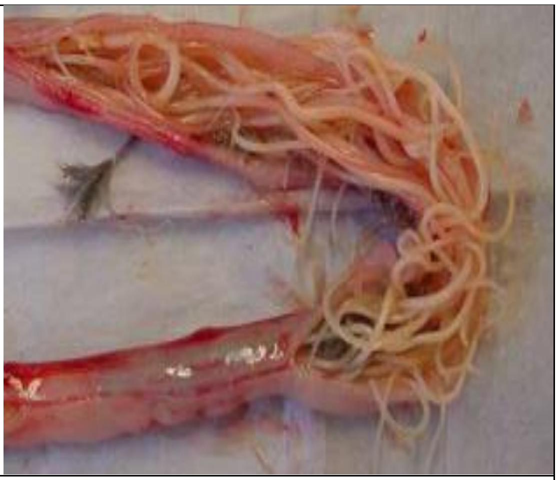
**Roundworms**	**Roundworms**

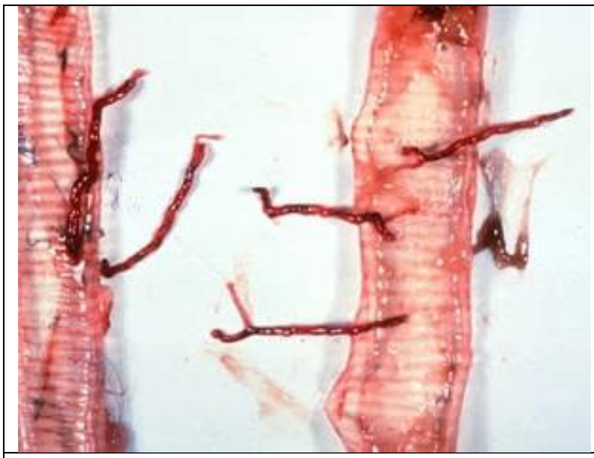	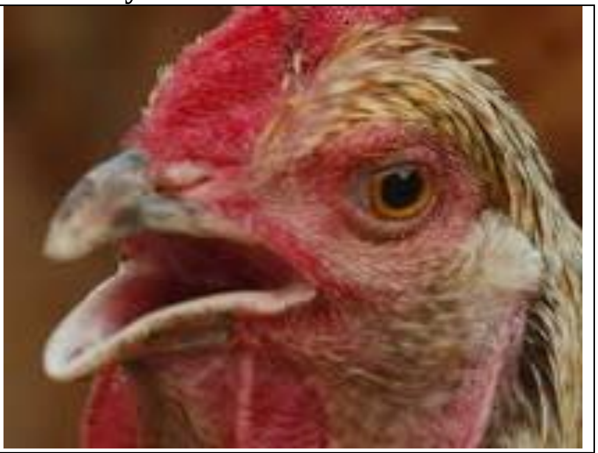
**Gape worms** (*Syngamus trachea*)	A hen gaping (breathing with open beak)
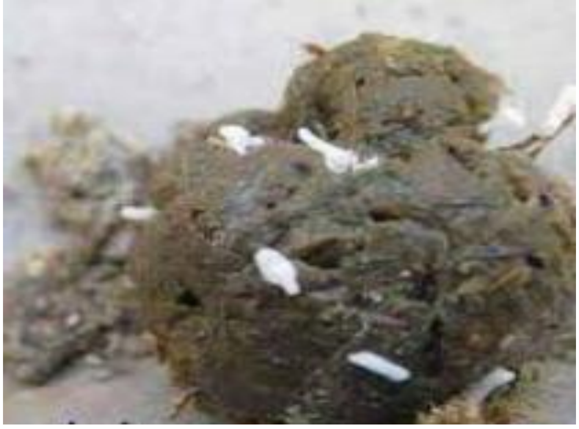	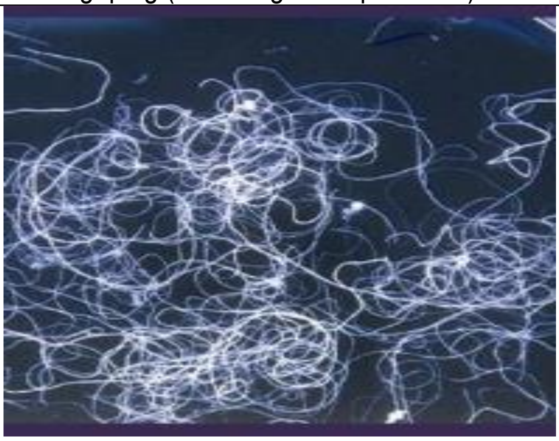
**Tapeworm segments**<br>Mostly found in chickens reared in free- range systems	**Hair worms (*Capillaria* species)**<br>❖ Mostly found in the crop<br>❖ Cause diarrhoea, anaemia, weight loss, loss of appetite and the birds can look depressed and dull

9. Scaly leg mite

❖ Mostly affects adult free-ranging chickens and turkeys<br>❖ They are extremely tiny round mites<br>❖ Burrow into the skin on the birds' legs and feet<br>❖ They dig tunnels, eat skin, lay eggs and leave droppings on the legs<br>❖ Result in crusty scales which are uneven and raised and thickened skin	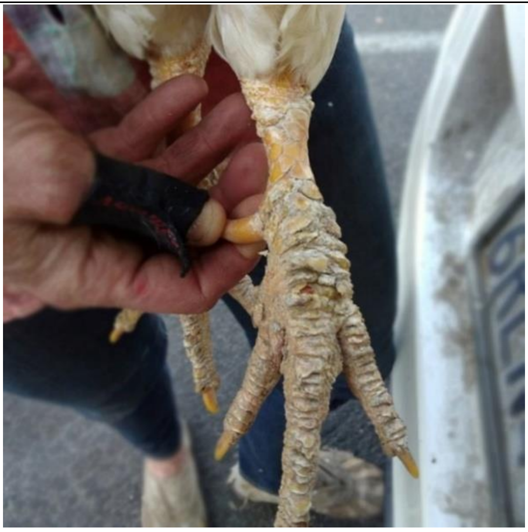

**Treatment**<br>1. Apply liquid paraffin or petroleum jelly (Vaseline) over the chicken's feet<br>2. Ivermectin: Given orally or topically in 3 doses at 0.2mg/kg body weight once every two weeks<br>3. Selamectin (topical avermectin)<br>4. Moxidectin: Pour-on or injectable forms are both effective and available in 0.5% and 1% preparation	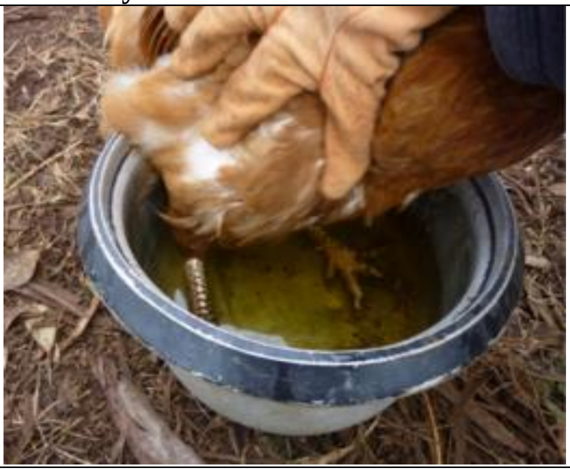

Other external parasites

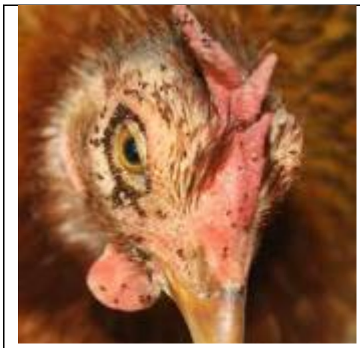	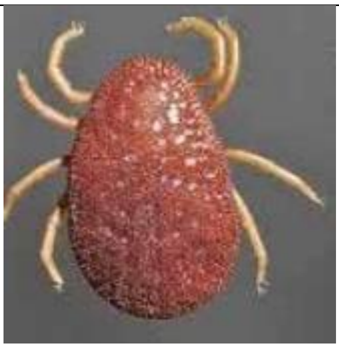	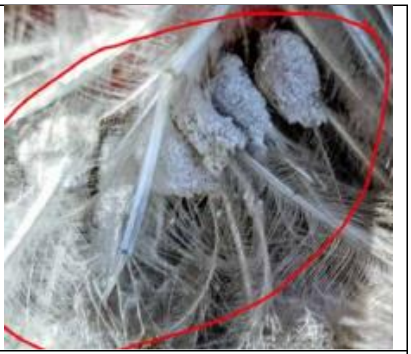
**Fleas**	**Ticks**	**Lice**
**Treatment:** Dusting with dudu dust (Sevin) and allowing the chickens to take ash bath		

Eating disorders

May be caused by vitamin and mineral deficiencies especially vitamin B and calcium.

**Curled toe paralysis**<br>❖ Caused by vitamin B (riboflavin) deficiency<br>❖ Poor growth and weakness<br>❖ Unable to walk as their toes are turned inwards<br>❖ Drooping of wings	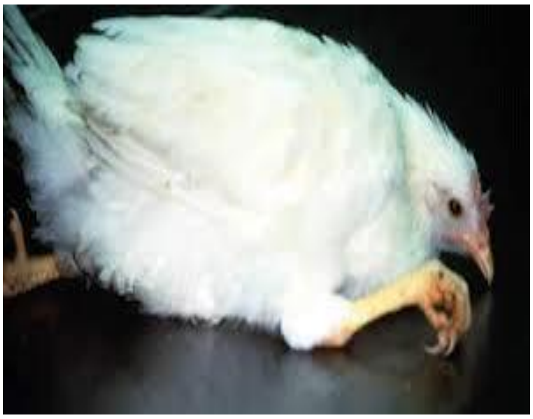

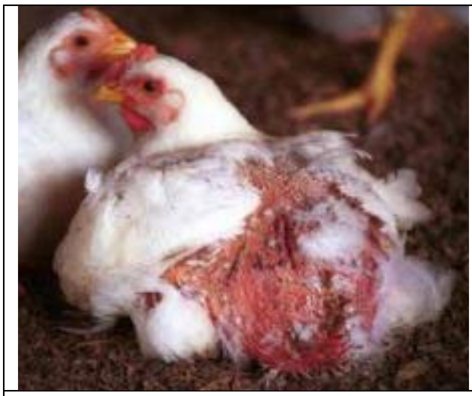	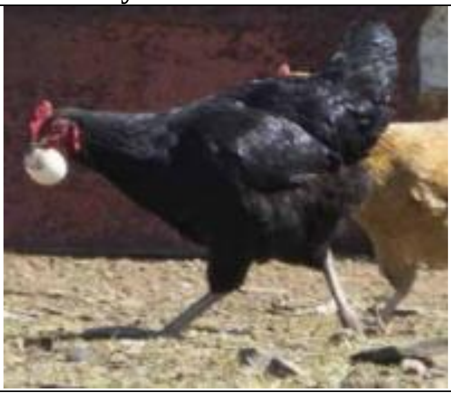
**Cannibalism -** pecking other chickens<br>❖ Linked to nutrient deficiencies<br>❖ Feed chickens a diet of sufficient protein<br>❖ Separate chickens being pecked to allow them heal	**Eating eggs of other hens or their own**<br>❖ Results from calcium deficiency<br>❖ Not easy to correct, cull such hens<br>❖ Give the chickens calcium supplements e.g boiled egg shells

MODULE 5: Goat husbandry practices

5.1 Introduction

This session introduces goat keeping in arid and semi-arid areas and the benefits that comes with this farming venture. It goes ahead to categorise the different kinds of goats kept in Kenya based on their different characteristics and their products. Furthermore, the session illuminates the different aspects of care for goats such as handling, feeding and managing stress. For improved health and production, the goat farmers are shown the importance of constructing a proper goat house. Finally, the farmers are shown the different diseases that affect goats and how they can keep them healthy

5.1.1 Session learning outcomes

By the end of this session, the learner will be able to:

I. Explain the importance of keeping goats in Kenya
II. Categorise the different breeds of goats kept in Kenya and their equivalent products.
III. List some of the causes of stress in goats.
IV. Outline how a farmer can keep his flock healthy
V. Describe the symptoms, prevention and treatment of Contagious caprine pleuropneumonia (CCPP).

5.1.2 Time required

This session should take one hour.

5.1.3 Materials/Planning needed

Slides, Charts, photographs

5.1.4 Training method(s)

Lecture method, group discussions, demonstrations

5.2 Why keep goats:

Goats are highly prolific animals that can perform well even in arid and semi-arid regions. They are important livestock especially in rural communities of Kenya because of the following reasons:

1. For milk: Produces nutritious milk high in calcium and phosphorus
2. Provision of meat: have tasty and soft meat
3. Source of income (from selling meat, milk, goats)
4. Require less space (6 goats can be kept in a space needed for 1 cow) and less feed compared to cows
5. Droppings used as manure for organic farming
6. Short gestation period (5 months)
7. High chances of twinning (sometimes giving triplets)
8. Require less initial capital investment and less labour to maintain
9. Can survive in places with water and feed shortages, rocky and even hot areas
10. They help to keep bushes under check, they stop too much shrub growth
11. Social roles: payment of dowry, traditional/religious rituals.

5.3 Goat breeds

There are three categories of goat currently kept in Kenya for meat, milk or both. These are:

I. **Indigenous breeds:**

- ❖ Are well adapted to harsh environment
- ❖ Are highly resistant to most diseases
- ❖ Feed on the locally available materials
- ❖ They are kept for meat production and other cultural purposes

Examples: Small East African goat, Galla goat (also called Somali and Borana goat).

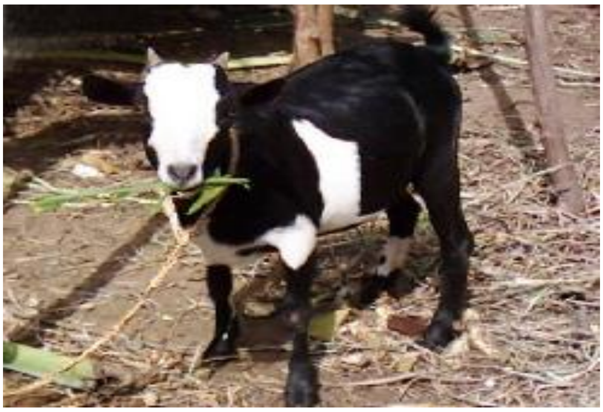

Figure 1. Small East African goat

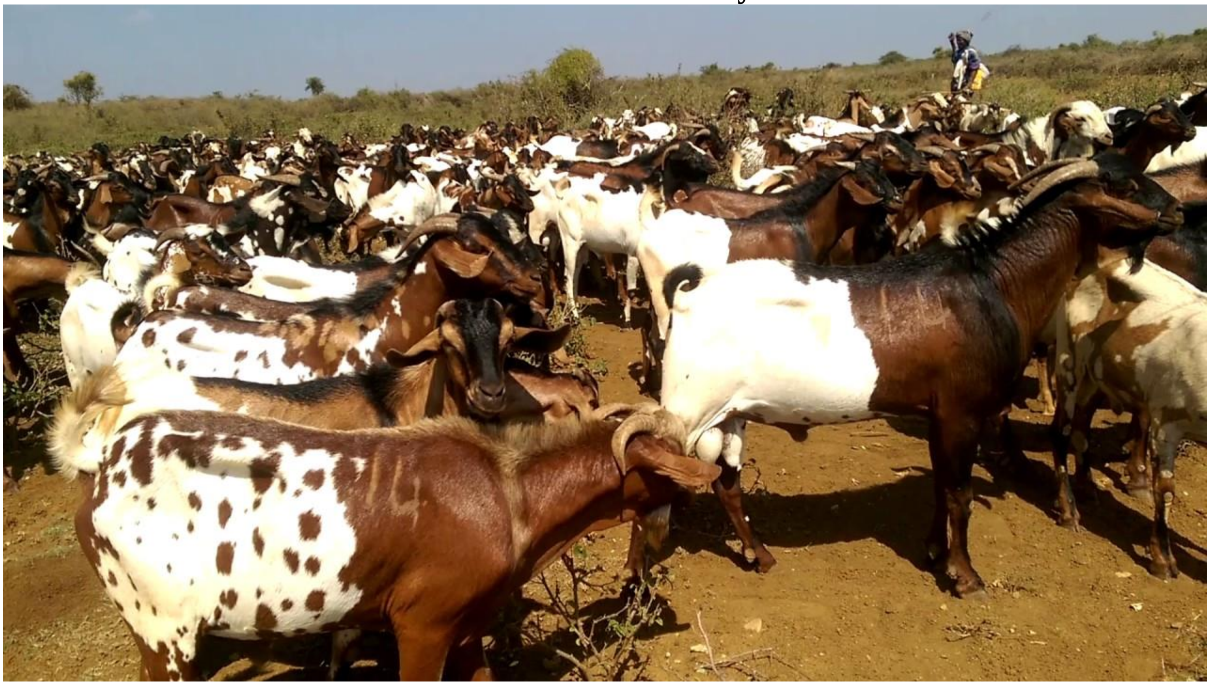

Figure 2. Somali goats

II. **Meat breeds:**

❖ Are kept for meat production<br>❖ They are more susceptible to diseases<br>**Examples** include Boer goats, Savanna goats and Kalahari red goats.	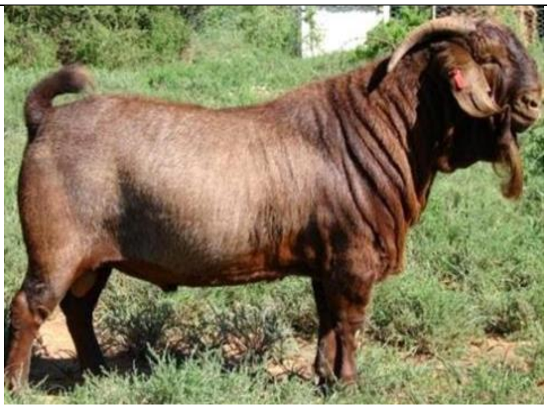<br>**Boer goats**

III. **Dairy breeds:**

- Are selected for milk production
- Examples are: Saanen, Toggenburg, German Alpines, and Anglo-Nubian.
- These breeds are more susceptible to diseases, parasites and extreme temperatures (hot)

**1. Saanen**<br>❖ Produces a lot of milk up to 7 litres per day<br>❖ White in colour<br>❖ Has high prolificacy-2 kids per birth<br>❖ Performs poorly in hot areas	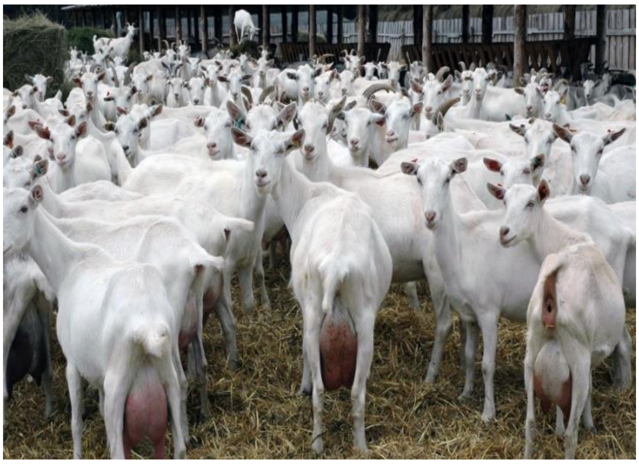<br>**Saanen dairy goats**

**2. Toggenburg**<br>❖ Brown (dark) in colour<br>❖ White areas on the face, legs and tail<br>❖ Produces up to 5 litres of milk per day<br>❖ Has a little bit higher butter fat content	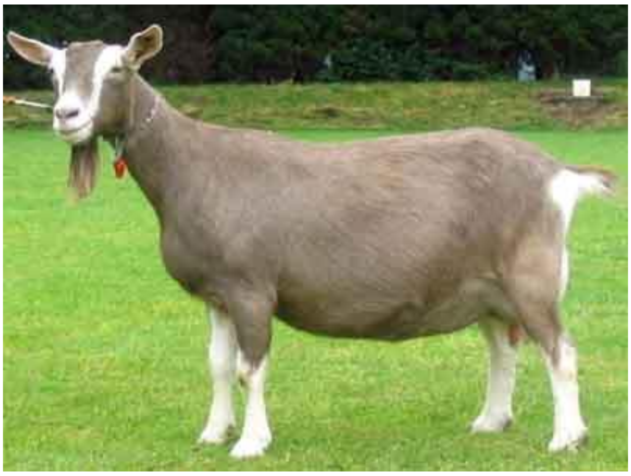<br>**Toggenburg dairy goat**

3. Anglo-Nubian

- ❖ Have long dropping ears
- ❖ Produces milk with a high butter fat content (milk good for making cheese)
- ❖ Produces up to 5 litres per day

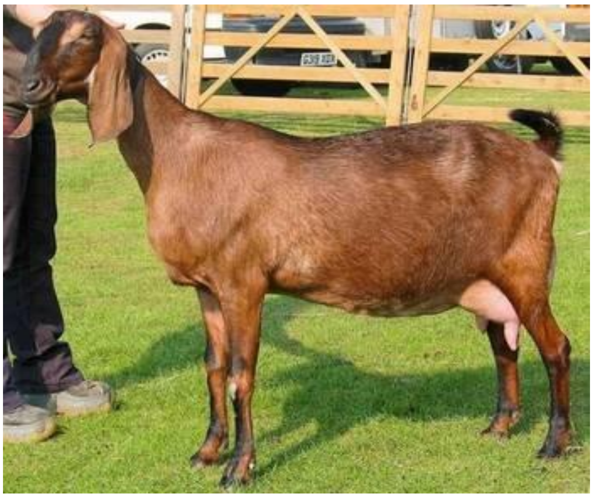

Anglo-Nubian dairy goat

4. German Alpine/British Alpines

- ❖ Are dark in colour with dark line on the back
- ❖ Produces up to 3 litres per day
- ❖ Are shorter

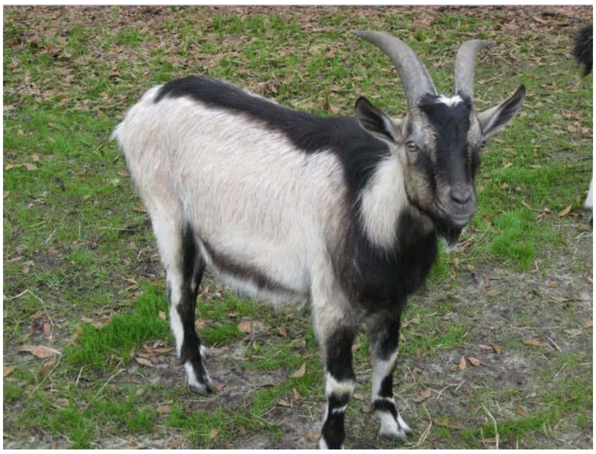

Alpine dairy goat

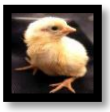

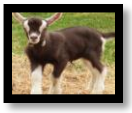

5.4 How to monitor performance of indigenous goats

The following facts can assist a farmer monitor the performance of his indigenous goats.

1. Gestation (pregnancy) period: about 5 months (150 days) (three births every 2 years)- Make sure that there is sufficient feed during the early stage (to prevent reabsorption of the foetus) and sufficient food during the *last 6-8 weeks* of pregnancy, when the foetus is growing fast,
2. Breeds throughout the year in tropical zones
3. Birth weight is around 2.5 kg
4. Weaning weight (weight when kids stop suckling): 12-15 kgs
5. Weight of mature females: 35-40 kgs
6. Weight of mature rams/males: 45-50 kg
7. Breeding age of young does: 9 months to 1 year
8. Male to female ratio: one (1) male to 25 females
9. Lifespan: 10-12 years
10. Castrate males at 3 months (using a burdizzo)
11. Goats walk on average 4-6 km per day. Distance increases with herd size and season.
12. They spend 75% browsing and 25% grazing, even with grass available.

5.5 How to handle goats

When handling goats, it is important to keep them calm and prevent injuries. Properly constructed pens and handling facilities allow for easy handling of the goats. Take note of the following natural behaviours of goats when handling them:

- Goats prefer to move towards light than darkness
- Are majorly browsers (not grazers)
- Goats prefer to stay with the flock, separation causes stress
- They like to follow dominant leader
- Tend to move in a circle in the pen around the handler
- Are easily distracted by noise
- Tend to fight a lot when confined and stressed
- Prefer to move in family groups
- Goats like feeding at knee height up to head height
- They like to feed above the ground often standing on their hind legs and resting their fore legs up on the bush or goat house wall
- Can easily jump over gates and escape
- Standing over the animal's shoulder will generally encourage it to move forward
- Keep the goat upright when trimming hooves
- Try to get the goats used to being handled so that they are less stressed
- When holding the goats by the horns, hold the base of the horns and not the tips
- Work calmly and quietly with your goats
- Goats must not be kept in longer than necessary as it reduces the number of hours available for feeding

5.6 Feeding and stress

A properly fed goat is generally a healthy animal. However, nutrition deficiencies often induce stress that ultimately lowers immunity and makes the animal more susceptible to diseases.

Goats should be given different types of feed for fast growth and fast weight gain. These include:

- **Fodder:** Napier grass, green leaves. The fodder should be clean, fresh, dry and chopped into small pieces of about 3cm. Feed only clean, fresh and dry fodder
- Add energy supplements, molasses, milling by-product e.g. pollard, bran, cereals etc.
- **Protein supplements:** such as calliandra leaves, cotton seed cake, leucaena leaves, desmodium, fish meal, dairy meal, sweet potato vines
- **Salt:** hang nutritious salt licks like Maclik Mineral Brick.
- **Water:** ensure goats gets sufficient supply of clean water (adult goat takes about 2 litres of water per day)
- Castrate the goats for fast weight gain
- Browsing/grazing should be opted for where there is space and supplements added (maize stalks, forages
- Provide fresh drinking water at all times
- Clean the feeding and watering troughs every day
- Give lots of different feeds such as grass, legumes, tree leaves, fresh kitchen remains,

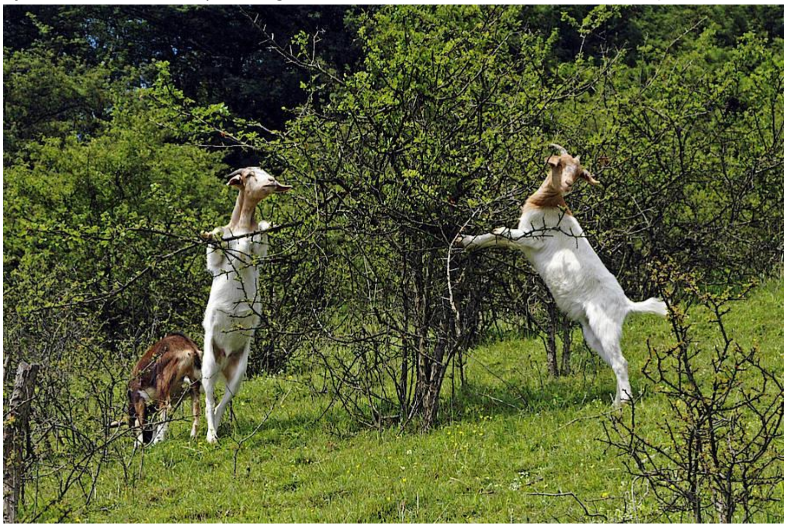

- Chopped mixed feeds can be given to ensure the goat eats everything
- Feed goats at least 3 times a day (same time every day)
- Get bored when fed the same plant everyday
- Can be wasteful only eating part of the plant

5.6.1 Causes of stress that should be taken into consideration

These include:

1. Hunger and thirst
2. Tiredness/exhaustion from walking long distances
3. Cold (exposure to wind, rain, wet floors)
4. Pregnancy: take good care of pregnant goats
5. Avoid sudden change in diet: any change should be gradual
6. Change in environment
7. Transportation

5.7 Housing structures

House goats at night to provide shelter from bad weather, prevent theft and predation.

A goat house should:

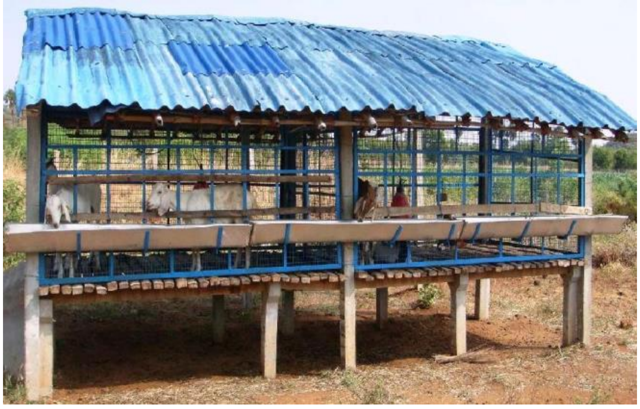

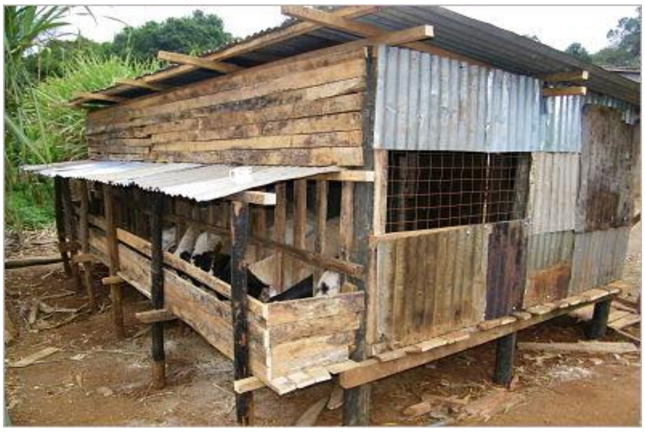

- Have adequate space (an adult goat requires about 0.5-0.75 m^2 of space)
- Be damp proof with non-leaking roof
- Free from sharp objects that can injure the goats.
- Free from pests and predators
- Well ventilated
- Free from direct wind
- Slated raised floor (1.5 ft or just below knee height from the ground) to allow free fall of droppings
- Be in a noise free environment
- Easy to clean feed troughs can be built with the house
- The house can be divided into a sleeping/resting area (spacious with soft bedding, good ventilation, well roofed) and a feeding area (with water trough, feed trough, slatted floor, feed racks, and a rain proof mineral block, well lighted and ventilated)
- A house of 1.8m by 1.8 m by 2.5m is adequate to comfortable hold 10 small goats

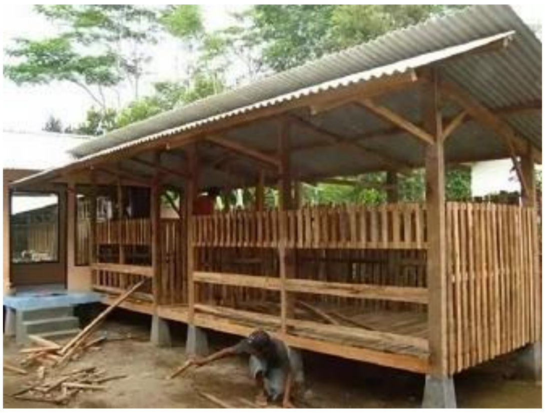

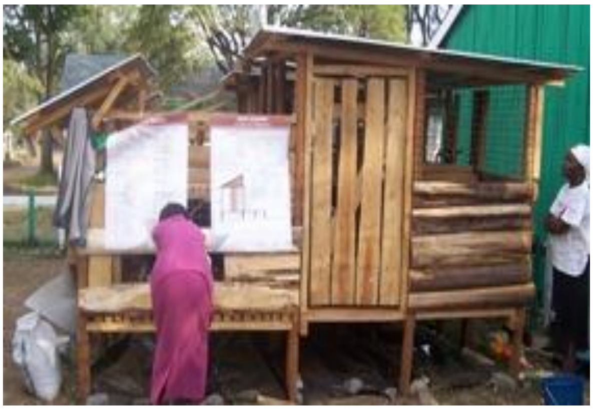

5.8 How to identify a healthy and sick goat

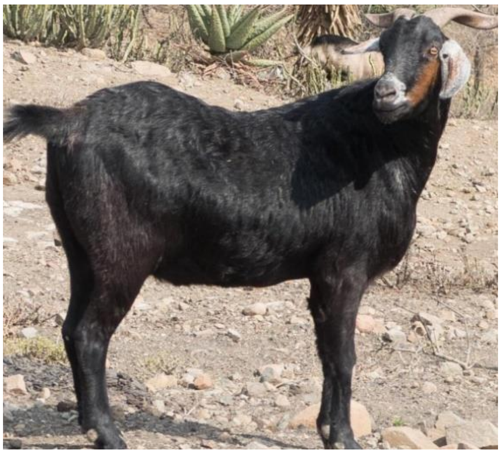

- Browse/eat feed as normally and chews cud
- Moves with the flock or herd of other animals
- Breathes normally, with ease and without panting
- Does not limp or bend its back while standing or walking
- Has a smooth and shiny hair coat
- Nose is moisten
- No discharges (excessive) from the eyes, nose,
- Shiny and clear eye balls
- Pink moisten mucous membranes (not white)
- Excretes normal faecal pellets (not loose or diarrhoea) and normal coloured urine
- Is not bloated (stomach not swollen)
- Rarely comes down with disease (has a good immunity)

5.9 Contagious caprine pleuropneumonia (CCPP)

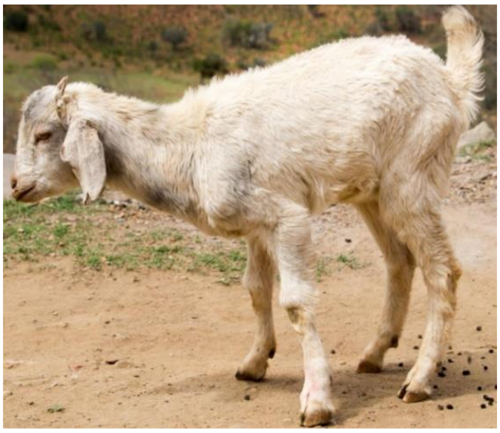

This is a disease that affects goats mainly but can also infect sheep. It spreads fast (can infect up to 100% of the goats) and kill about 60-100%.

Infected goats present with the following symptoms

- Stop feeding (lose appetite)
- Weakness
- Increased body temperature (41°C)
- Difficulty breathing
- Increased breadths per minute
- Coughing
- Discharges from the nostril
- Loss of weight

5.10 Treatment: contact a veterinarian to administer:

- Tylosin to sick goats for 3 consecutive days
- Tetracycline

5.11 Prevention

- Vaccination of all the goats every six (6) months
- Isolation of sick goats to minimise spread
- Quarantine impositions: when you goat is sick, notify the county veterinary officers

5.12 11.1 How to keep your flock healthy

- ❖ Separate/isolate sick animals so that they do not spread the infection to other animals
- ❖ Keep the enclosures/houses of the goats clean
- ❖ Properly identify your goats to avoid loss through theft (ear tags, tattoos, etc)
- ❖ Protect the goats from cold and wet conditions to prevent stress

**Deworming**<br>❖ Routinely deworm the goats every 2-3 months to control worms and flukes<br>❖ When deworming, be careful not to block the goat's nose, as this gets dewormer in the lungs and this can kill the animal<br>❖ Insert the tip of the syringe in the corner of the mouth as shown in the picture.<br>❖ Push the syringe deeper into the mouth to prevent wastage and helps safe dosing	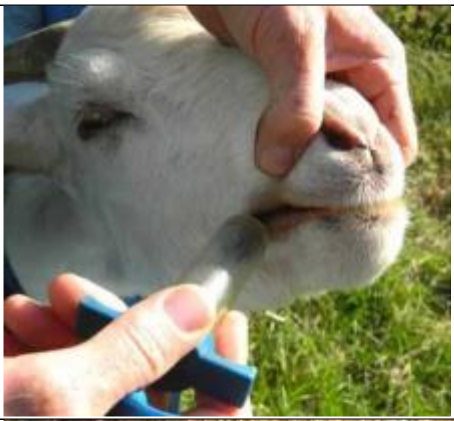
**Vaccination**<br>❖ Vaccinate goats against these common diseases:<br>1. CCPP vaccine given at 1 month of age, and every 6 months thereafter<br>2. PPR vaccine should be given at 2 weeks of age<br>3. Goat pox is given any time after 1 month of age<br>4. Tetanus toxoid	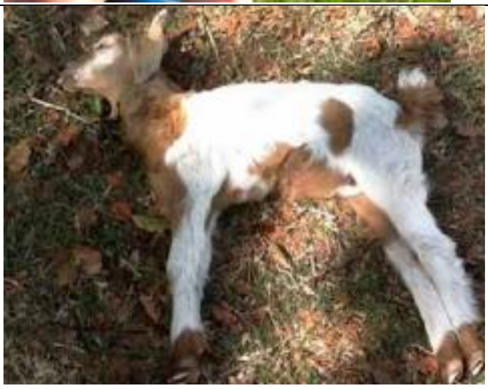
Ectoparasite control<br>❖ Dip/spray the goats every 2 weeks with acaricides to get rid of ticks, fleas, lice, mange and nasal bots<br>❖ Graze/browse the animals in areas without parasites (ticks)	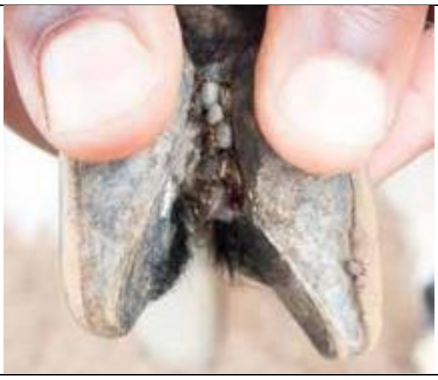

**Hoof care**<br>❖ Walk the goats through a foot bath of 5% copper sulphate regularly to prevent and control foot rot and foot abscess<br>❖ Regularly trim overgrown hooves and avoid bleeding them	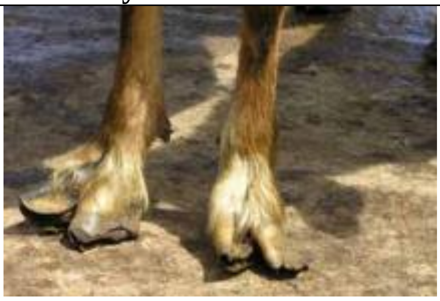

5.13 Other Important diseases of indigenous goats

**Peste des petits ruminant (PPR)**<br>The disease presents with:<br>❖ Fever<br>❖ Sores in the mouth<br>❖ Diarrhoea<br>❖ Breathing difficulty<br>❖ Sometimes death<br>❖ Has no treatment, symptoms managed by antibiotics	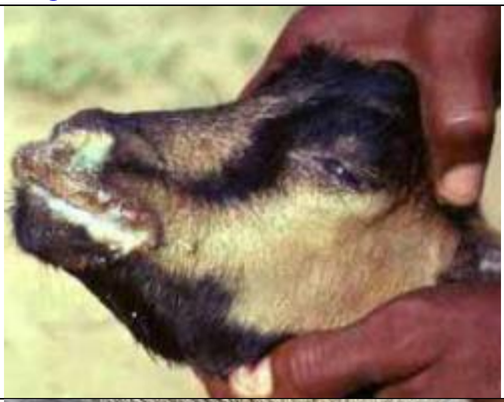
**Pneumonia**<br>Presents with:<br>❖ Fever<br>❖ Lack of appetite<br>❖ Rapid breathing<br>❖ Coughing,<br>❖ Loss of condition<br>❖ Discharge from the nose.	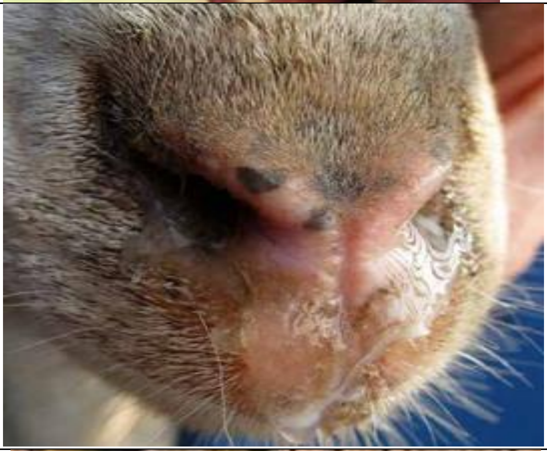
**Tapeworm (turning disease)**<br>❖ Goats with intermediate stages of tapeworm turn in circles<br>❖ Loses weight as it no longer eats and eventually dies.<br>❖ It can spread these tapeworms to humans if the meat is not cooked properly	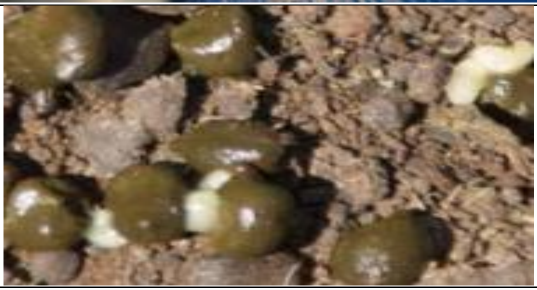

**Foot and mouth disease**<br>❖ Lesions (sores) in the mouth and on the feet,<br>❖ Salivation<br>❖ Lameness	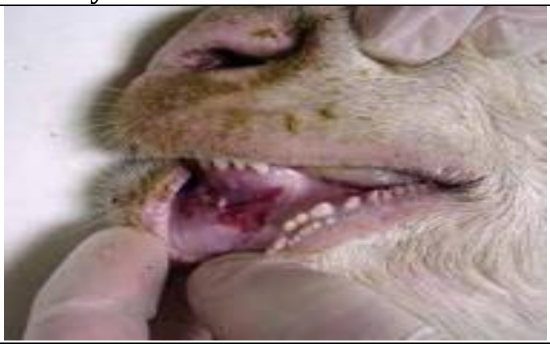

**Diarrhoea**<br>❖ Diarrhoea (may be bloody or contain mucus and be brown, yellow or greenish in colour)<br>❖ May be caused by bacterial infection, coccidiosis, worms<br>❖ Become thin/lose weight<br>❖ A rough hair coat	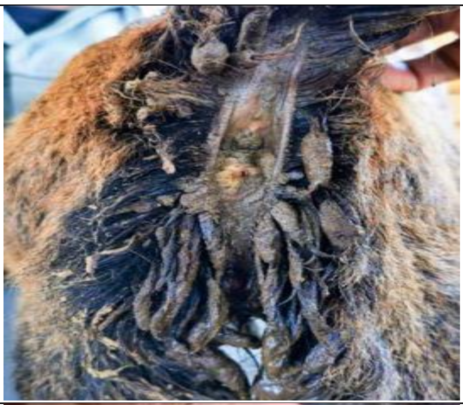
**Pink eye**<br>❖ The eye produces a lot of tears, becomes red and swollen<br>❖ Eventually the cornea grows cloudy and the animal becomes blind<br>❖ Separate sick animals and avoid stress and hunger	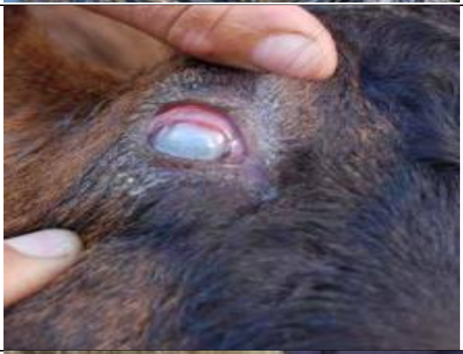
**Orf**<br>❖ Wart-like sores on the animal's lips and nose and around the mouth of especially young lambs and kids and on the teats of their mothers<br>❖ Separate sick goats to prevent the spread of the disease<br>❖ Hard scabs can be softened with Vaseline or glycerine to make it easier for the animals to eat<br>❖ Do not handle sick goats without gloves, disease can affect the hands of humans	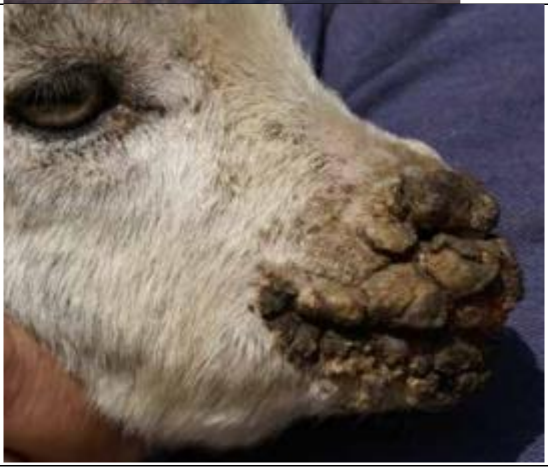

**Abortions**<br>Loss of foetus before term caused by:<br>❖ Infections causing fever e.g. Brucellosis, Chlamydiosis, heartwater<br>❖ Poor nutrition at late stage of pregnancy<br>❖ Mineral deficiency<br>❖ Stress	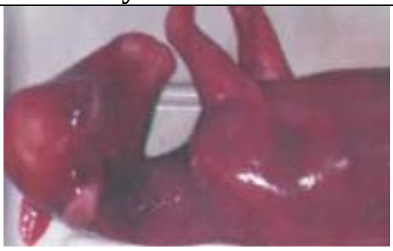
**Bloat**<br>❖ The animal's stomach swells from eating a lot of maize gains, green Lucerne or clover<br>❖ It becomes uncomfortable and may lie down and cannot breathe and will die<br>❖ Withdraw drinking water<br>❖ Make the goat drink cooking oil (50 ml) or bloat guard<br>❖ Contact a vet	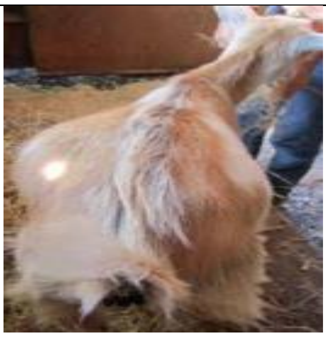
Heart water	Mastitis
Black quarter	
Anthrax	

5.14 11.4 Record keeping

Goat farmers need to keep the following records:

1. Number of goats currently owned
2. Expected dates when does will give birth
3. Number of kids born
4. Number of goats that die and cause of death
5. Treatments given
6. The mother of each goat
7. New introductions
8. Ages of all the goats
9. Number of goats sold, when they were sold and prices.

Evaluation Activity:

1. As Makueni farmers how do you handle your goats?
2. What are some of the qualities of a good goat house?

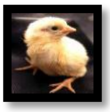 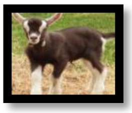

Appendix 1: Ration formulation from locally available materials

The amounts of energy, protein, minerals and vitamins in the ration will be depend on age, purpose of rearing, nutritive value, daily intake and cost. Local feed ingredients used for local chickens

- ❖ Fully confined and semi-confined chickens should be fed different types of commercial diets.
- ❖ The diet of chicken is divided into three distinct categories with decreasing amount of protein as starter/chick, grower and layer diet.
- ❖ The ration formulated for chicks should contain more protein nutrient until age of 8 weeks (0-8 weeks) as shown in the next table:

**Ration formulation formula for starter, growers and layers**			
**Ingredient**	**Starter/chick (%)**	**Grower (%)**	**Layer (%)**
Maize	30	25	35
Wheat	20	25	
Wheat bran	10	15	
Rice bran	10	10	35
Sunflower cake	10	5	
Cotton seed cake	5	11	
Fish meal	2	2	15
Beans	10	5	
Limestone	0.5	0.5	2
Salt	0.5	0.5	0.5
Mineral premix	1	1	1
Soya cake			10
Ground dried legume leaves			3.5

**A simple ration for supplementing kienyeji chicks from day 1 to 8 weeks**	
**Ingredient**	**Quantity**
Crushed maize/sorghum	1 kg tin
Wheat/sorghum or millet bran	1 kg tin
Sunflower/sesame/groundnut cake	2 match boxes
Fish meal/salt mix	1 match box
Sesbania/leucaena leaves	2 match boxes
Termites or maggots if available can be added during first 8 weeks	

**Amount of feed kienyeji chickens at different ages**	
**Age (in weeks)**	**Intake/bird/day (grams dry weight)**
1 week	12-15
2 weeks	15-21
3 weeks	21-35
4-6 weeks	35-50
7-8 weeks	55-60
16-27 weeks	68-80
28 weeks	100
Allow a maximum of 30 - 40 g/bird/day from week 4 - 6 and gradually reducing the supplementary feeding. As the birds grow, they will gradually get a smaller portion of what they need, until they only get between 1/3 and half of their needs as adults. To ensure sustained egg production, offer feeds when the chicken need it rather than less quantities during the harvest season and more during the lean season	

Appendix 2: Vaccine regimes for hybrid chickens but also applicable to kienyeji chickens

(Sigma feeds recommendation)

**Age**	**Vaccine against**	**Mode of application**
**1st week**	Marek and Newcastle	Subcutaneous injection (neck)
**2nd week**	Gumboro	In drinking water
**3rd week**	Newcastle + IB	Drinking water or eye/nostril droplets
**4th week**	Deworming, IBD	Drinking water
**5th week**	Newcastle+ IB	Drinking water
**6th week onward**	Fowl pox	Wing web stab
**6th-8th week**	Fowl typhoid	injection
**9th week**	Deworming (every 2-4 weeks)	Drinking water
**12-14th week**	Fowl typhoid	Injection
**16-18th week**	Newcastle (where NCD is prevalent)	Optional
