# Supplementary material for: Effectiveness of a community-centered Newcastle disease vaccine delivery model under paid and free vaccination frameworks in southeastern Kenya
Source: PLoS One. 2024 Aug 1;19(8):e0308088. doi: 10.1371/journal.pone.0308088 (PMC11293705; doi:10.1371/journal.pone.0308088)

The University of Nairobi

The Cooperative University of Kenya

Kenya Agricultural & Livestock Research Organization

PRACTICAL GUIDE TO TRAINING OF COMMUNITY CHICKEN VACCINATORS IN MAKUENI COUNTY

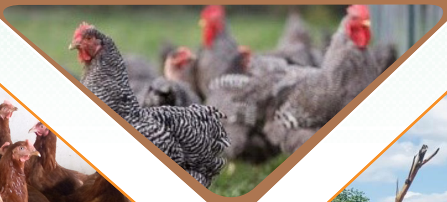

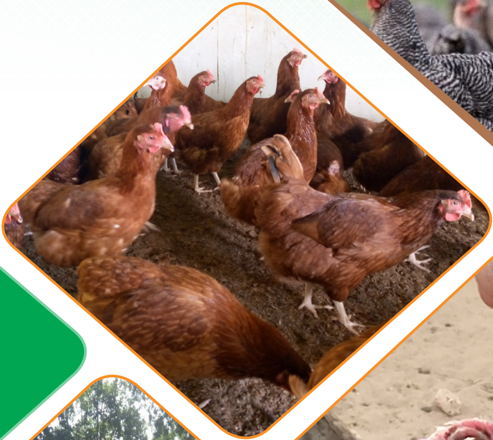

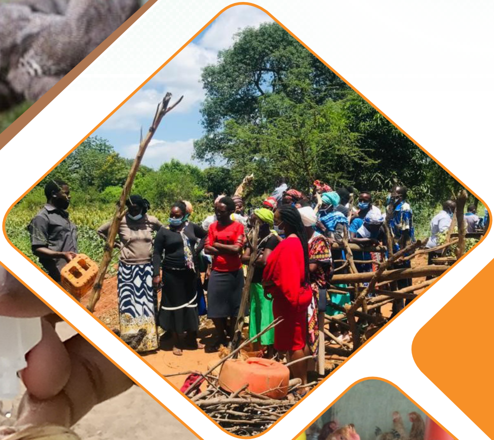

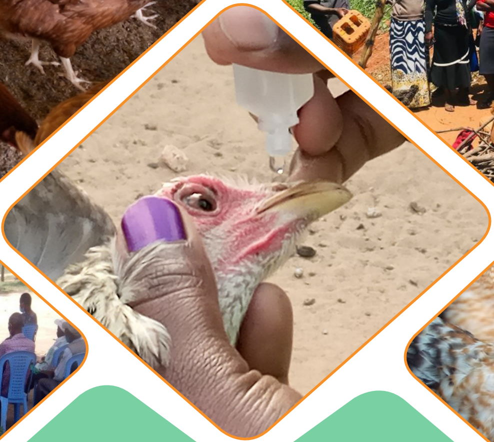

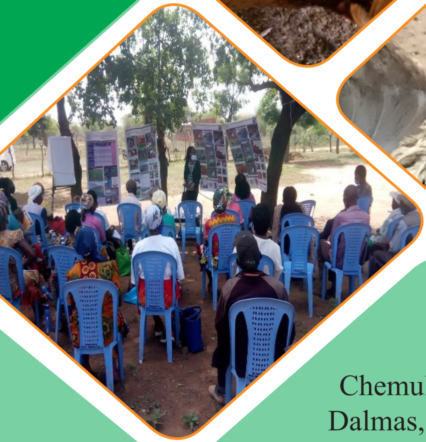

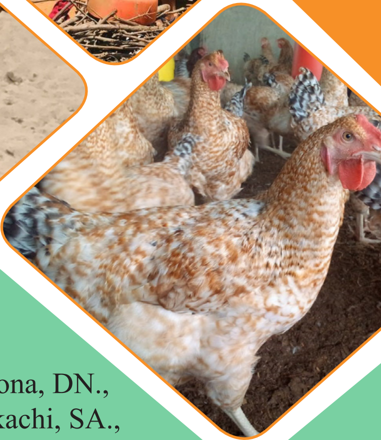

Chemuliti, JK., Ogolla, KO., Anyona, DN., Dalmas, OO., Nyamongo, IK,, Bukachi, SA.,

The University of Nairobi

The Cooperative University of Kenya

Kenya Agricultural & Livestock Research Organization

PRACTICAL GUIDE TO TRAINING OF COMMUNITY CHICKEN VACCINATORS IN MAKUENI COUNTY

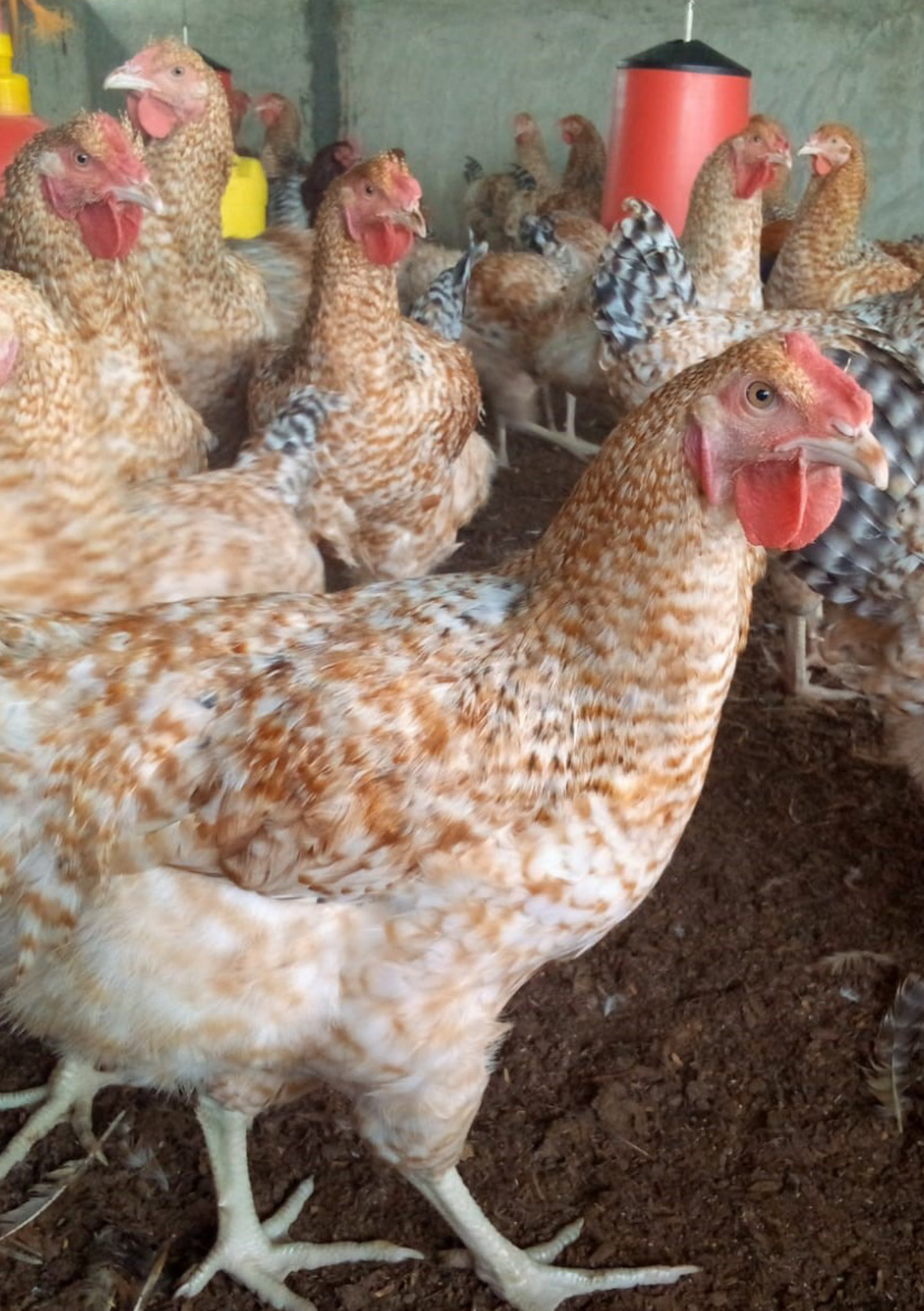

Table of contents

Foreword......vii
Acknowledge......viii
1. Introduction......2
1.2 Objectives......2
1.3 Target audience......2
1.4 Training philosophy......2
1.5 Layout/content of the manual......2
SESSION 1......4
2 Definition and qualities of a good community chicken vaccinator......5
2.1 Introduction......5
2.1.1 Session learning outcomes......5
2.1.2 Time required......5
2.1.3 Materials/preparation needed......5
2.1.4 Training methods......5
2.2 Who is a community chicken vaccinator?......6
2.3 Characteristics of a good community vaccinator......6
2.4 Working with farmers......7
2.5 Assessment Questions......8
SESSION 2......9
3 Introduction to New castle disease......10
3.1 Introduction......10
3.1.1 Session learning outcomes......10
3.1.2 Time required......10
3.1.3 Materials/preparation needed......10
3.1.4 Training methods......10
3.2 What is Newcastle disease and what causes it?......11
3.3 Where does Newcastle disease come from?......12
3.4 How does the disease spread?......13
3.5 Treatment of Newcastle disease......14
3.6 Prevention of Newcastle disease......14
3.7 Assessment Questions......15
SESSION 3......16
4 Vaccines and vaccination......17

4.1 Introduction..17
4.1.1 Session learning outcomes..17
4.1.2 Time required..17
4.1.3 Materials/preparation needed..17
4.1.4 Training methods...17
4.2 What are vaccines?...18
4.3 Importance of vaccinating chicken..................................19
4.4 How do vaccines work?...19
4.5 How vaccines are packed...21
4.6 Number of doses per vial of vaccine................................22
4.7 Vaccine handling and storage..22
4.8 Transporting vaccines..22
4.9 Assessment activity/questions...23
SESSION 4..24
5 Administration Newcastle disease vaccine................................25
5.1 Introduction..25
5.1.1 Session learning outcomes..25
5.1.2 Time required..25
5.1.3 Materials/preparation needed..25
5.1.4 Training methods...25
5.2 Reconstitution of Newcastle disease vaccine....................26
5.3 Eye drop or nasal drop vaccines......................................27
5.3.1 How to use a syringe and needle?.................................27
5.3.2 Eye dropper...28
5.3.3 Directions for carrying out nasal/eye drop vaccination.28
5.4 How not to handle chickens...29
5.5 Vaccinating chicken..30
5.6 How to take care of the plastic droppers to ensure long ...... life...31
5.7 Water vaccination (in drinking water)..............................31
5.7.1 Direction for use of vaccines in drinking water .... administration of vaccine...32
5.8 Assessment questions...33
SESSION 5..34
6 General disease control and prevention methods......................35
6.1 Introduction..35

6.1.1 Session learning outcomes..35
6.1.2 Time required...35
6.1.3 Materials/preparation needed...35
6.1.4 Training methods..35
6.2 Control and prevention of diseases in chicken..................35
6.3 Reasons why vaccination may fail..................................37
6.4 Assessment questions..37
SESSION 6...38
7 Participating in chicken vaccination campaigns and keeping ...... records..39
7.1 Introduction..39
7.1.1 Time required...39
7.1.2 Materials/preparation needed...39
7.1.3 Training methods..39
7.2 Participating in a chicken vaccination campaign..............39
7.2.1 What is a chicken vaccination campaign?.........................39
7.2.2 Participating in a chicken vaccination campaign.............40
7.3 Record keeping for community vaccinators......................41
7.4 Vaccine regimes for local chickens but also applicable to ... indigenous chickens..41
7.5 Importance of targeting both women and men farmers for . the vaccination...42
7.6 Assessment questions..42

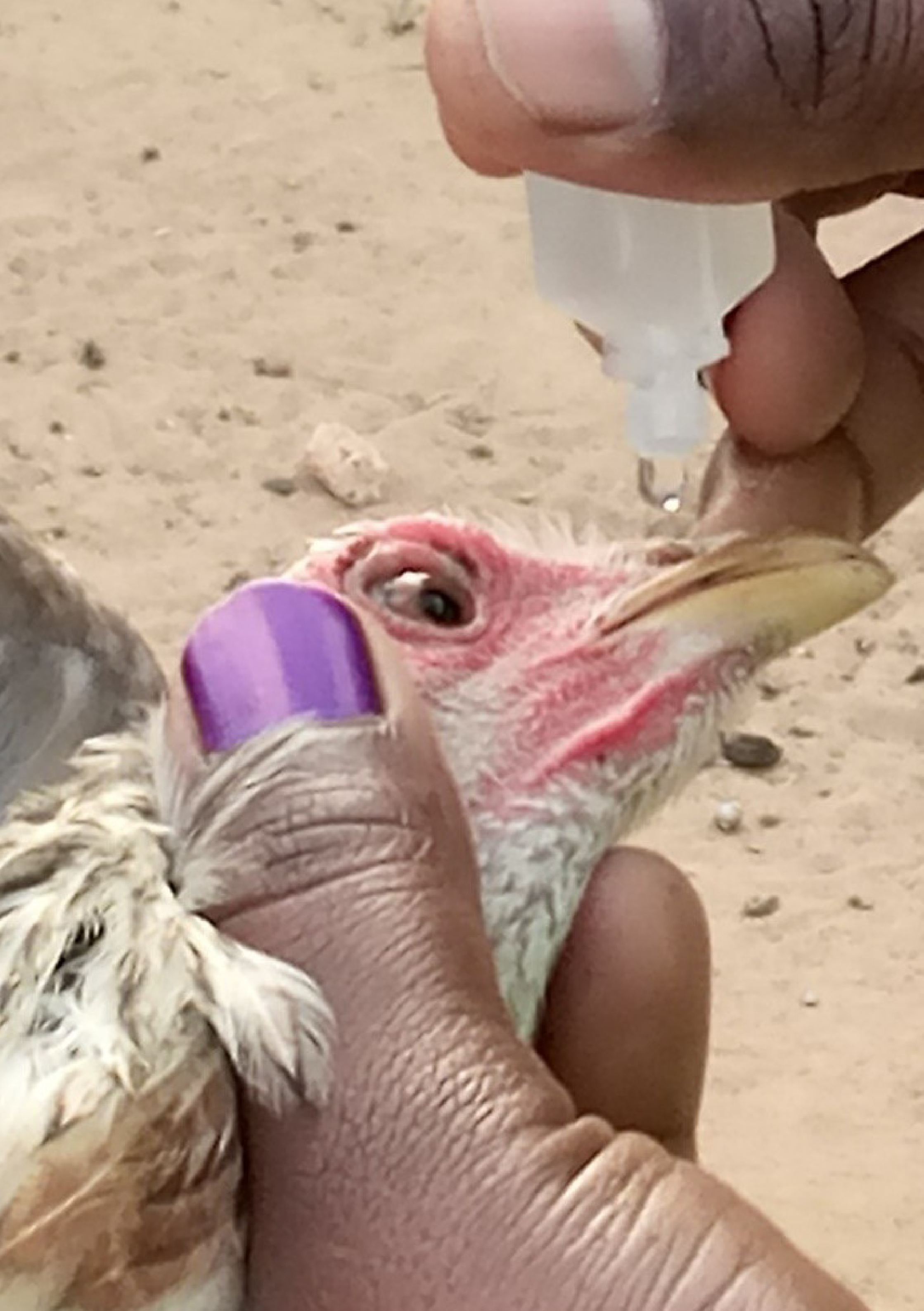

Foreword

Indigenous chicken production is an important livelihood activity for many households in Makueni county. However, many smallholder farmers continue to encounter a number of problems, one being the heavy disease burden. Of the notable chicken diseases, Newcastle disease has been identified as among the most constraining. With funding from International Development Research Centre (IDRC), the Biotechnology Research Institute of the Kenya Agricultural and Livestock Research Organization (KALRO-BioRI), The University of Nairobi (UON) and the Cooperative University of Kenya (CUK) carried out a four-year study whose overall goal was to empower smallholder farmers especially women in indigenous chicken production through improved access and use of Newcastle disease (ND) and contagious caprine pleuropneumonia vaccines (CCPP). In part, the project identified the absence of poultry vaccinators and the large-dosed Newcastle disease vaccine vials as the main reasons for low vaccination rates, significantly affecting productivity. This training manual has, therefore, been developed as a tool to equip frontline disease control field practitioners (veterinary personnel, private service providers) and community vaccinators with the requisite skills to effectively redress this gap in the control of Newcastle disease in the county.

Acknowledgement

We are grateful for financial support from the International Development Research Centre (IDRC) grant number 109063 which enabled us to develop this manual.

We thank the Directorate of Veterinary Services, Department of Agriculture, Irrigation, Livestock and Fisheries in the county government of Makueni for facilitation in conducting fieldwork.

Our sincere thanks also go to the smallholder farmers in Makueni county for providing valuable information that went into the development of this manual.

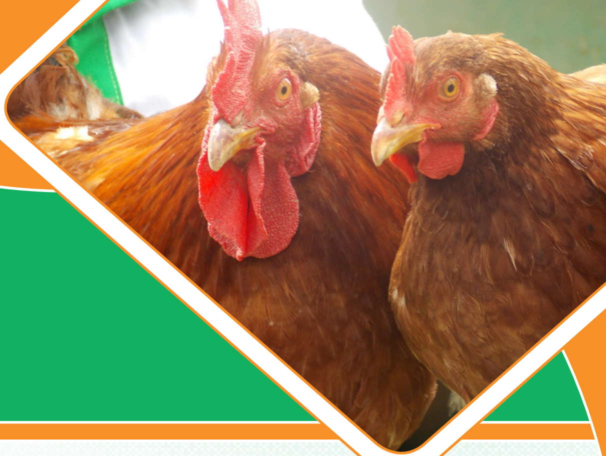

1. Introduction

Newcastle disease is the most significant impediment to profitable smallholder chicken production in Kenya. The disease causes high mortality of up to 80 to 100%. Control of Newcastle disease (ND) in village chickens can significantly and positively contribute to smallholder farmers' livelihoods and women's empowerment in rural areas. Newcastle disease vaccine is available but access and utilization is still low among smallholder indigenous chicken producers in many low-resource settings in rural Kenya. Small flock sizes, large-dose vaccine vials, high vaccine acquisition costs, lack of vaccine knowledge, and limited numbers of animal health service providers are some of the obstacles to widespread use of the ND vaccine among smallholder chicken producers. Recognizing the importance of smallholder chicken rearing and its potential role in delivering livelihood benefits to smallholder farmers, especially women, many agricultural programs and projects are implementing interventions to improve productivity and incomes. Such interventions include the provision of Newcastle disease vaccination services through trained community chicken vaccinators. However, there is no clear guidance, and a lack of standardization of the training of such community vaccinators which may affect the quality and effectiveness of their service. This manual provides evidence-based information and guidance on training community chicken vaccinators. It is an output of a four-year ND vaccine intervention study of smallholder indigenous chicken systems in Makueni County where women and men community chicken vaccinators were recruited, trained, and deployed

to offer Newcastle disease vaccination services to farmers. The quality of service and performance of the vaccinators was monitored and evaluated over a 12-month period, hence, the manual builds on the findings and lessons learned in the study.

1.2 Objectives

This manual is intended for use in the training of community chicken vaccinators in order to increase their capacity and skills in providing programmatic vaccination services to chickens against Newcastle disease.

1.3 Target audience

This manual is prepared for use by veterinarians and veterinary paraprofessionals in the training of community chicken vaccinators to offer Newcastle disease vaccination services in areas where such professionals are unavailable. It provides the facilitator with brief notes to guide their effective delivery of various sessions.

1.4 Training philosophy

The philosophy of this training manual is participatory and inclusivity, and includes learning-by-doing activities with a focus on adult learning methods that require participation through small and large group discussions, brainstorming, role-play, and practices in a classroom setting.

1.5 Layout/content of the manual

The manual is divided into six sessions that contain training content and a guide on how to conduct the sessions. Each session contains the following;

1. Learning outcomes for the session
2. Time required for the session
3. Materials/preparation needed for the session
4. Training methods
5. Session content

SESSION 1

2. Definition and qualities of a good community chicken vaccinator.

2.1 Introduction

The session sets the stage for the entire training by defining and explaining to the trainees the qualities of a good community vaccinator.

2.1.1 Session learning outcomes

By the end of this session, the trainees will be able to:

i) Define a community chicken vaccinator

ii) Discuss the characteristics and qualities of a good community vaccinator

iii) Describe how a community vaccinator can work with farmers effectively.

2.1.2 Time required

This session should take approximately 2 hours.

2.1.3 Materials/preparation needed

Slides, charts, photographs, writing materials

2.1.4 Training methods

Lecture method, group discussions, demonstrations

Facilitator guide

Before the beginning of the first session, the facilitator welcomes trainees and leads them in self-introduction clearly stating their profile (name and home area) and briefly stating the broad objective of the training. Trainees are then invited to share their experiences in chicken rearing and animal health-related challenges including diseases. The trainees are then invited to state their training expectations. The facilitator takes note of the specific training needs and customizes the delivery to meet the needs. At the end of each session, the facilitator will ask the trainees questions to assess if they have understood the session and re-emphasize the key points if necessary.

2.2 Who is a community chicken vaccinator?

This a person, woman or a man in their diversity, without professional training in animal health who has willingly volunteered to receive short training on Newcastle disease vaccination in chickens and offer project-level vaccination services to fellow farmers in their area/ village under the supervision and guidance of a qualified animal health professional. The vaccinator is highly regarded in their community and acceptable among their peers. The vaccinator has been trained on Newcastle disease vaccination and basic chicken husbandry practices. S/he appreciates that her/his role is limited to Newcastle disease vaccination in village poultry. S/he acknowledges that a community vaccinator is not a veterinary or animal health expert and acknowledges that she does not know everything and where necessary and possible consults with and refers farmers to the relevant extension office. S/he provides information on her activities to the relevant regulatory bodies when called upon. The vaccinator restricts their services to vaccination of poultry only and not any other types of livestock.

2.3 Characteristics of a good community vaccinator:

A good community chicken vaccinator has the following qualities:

1. Good understanding of local conditions, people's situation, and feelings.
2. Perseverance – one who understands that new ideas like regular vaccination of poultry will take time before the community fully embraces it.
3. Professional – remains professional yet careful to avoid oversharing or building too strong a connection with people, as this could be unprofessional and unsafe.
4. Organised – able to plan her/his activities well to ensure a balance between her/his role and responsibility as a community vaccinator and her/his personal and familial responsibilities.

5. Good communicator – able to communicate with many different people. Able to adapt his/her communication style to fit each person and situation. This is something you will learn and develop with practice.

2.4 Working with farmers

Community vaccinators should foster a good working relationship with farmers. This implies the following:

- There should be a good working relationship between the vaccinator and the farmer.
- The vaccinator should be able to give the farmer sufficient information to help them understand the importance of vaccinating poultry.
- If the farmers are to pay for the service, they must be convinced that it is beneficial for their chickens. It is the farmers who decide whether their chickens are vaccinated or not. However, community vaccinators should be able to give them enough information to help them make an informed decision.
- Since farmers are busy, vaccinators should plan their visits to fit times convenient to farmers (when farmers say they will be available).
- Chickens in rural setting are mostly owned by the women. Vaccinators should device ways of talking to the women without causing problems with the husbands. This may be achieved by first organizing a general meeting with community members and ensure the community supports and understands the importance of working with women chicken owners.
- On the first visit to the farmer, ensure that all family members are present.

- Always be punctual (on time) and avoid keeping farmers waiting. In case you are late due to unavoidable circumstances, apologise to the farmers who were waiting
- Always be polite.
- Inquire from the farmer if all of his/her chickens are in good health. Sick birds should not be vaccinated.
- Ask the farmer to assist you in holding the chickens during vaccination and show them how to hold the bird on the side for ease of vaccine administration (eye drop).
- Ensure the farmer understands the vaccine will only protect the chickens against the disease it is given for.
- Inform the farmer that chickens in poor body condition and those infested with parasites may not respond well to vaccination.
- Remind the farmer that Newcastle vaccination should be given every 4 months
- Chickens may be eaten immediately after vaccination should they want to
- Ask the farmer if he/she has a question
- Record the farmer's name, number of chickens raised and number vaccinated

2.5 Assessment Questions

1. Explain any three characteristics of a good community chicken vaccinator.
2. How can peer educators ensure that they foster a good working relationship with farmers?
3. Explain some of the things you are not supposed to do when handling chickens.

SESSION 2

3. Introduction to Newcastle disease

3.1 Introduction

In this session, we introduce the trainees to Newcastle disease. Drawing from beliefs and experiences of local farmers we explain the causes, symptoms and spread of the disease. We also enlighten farmers on how they can help prevent and treat Newcastle disease.

3.1.1 Session learning outcomes

By the end of this session, the learner will be able to:

i) Explain the causes of Newcastle disease

ii) Discuss the symptoms of the Newcastle disease

iii) Describe how Newcastle disease is spread

iv) Explain the prevention of Newcastle disease.

3.1.2 Time required

This session should take one hour.

3.1.3 Materials/preparation needed

Slides, flipchart, photographs

3.1.4 Training methods

Lecture method, demonstrations, group discussion.

3.2 What is Newcastle disease and what causes it?

Facilitator's guide

Ask trainees to name and characterise chicken diseases in their locality. Help them appreciate the difference between distinct chicken diseases and signs of disease/infection. Show them pictures of Newcastle disease-infected chicken and ask what they think the chicken could be suffering from. Use the list below to reinforce the key characteristics of Newcastle disease:

- Drooping wings
- Greenish diarrhoea
- Twisted necks
- Many deaths

Ask the trainees to describe and discuss the reasons why people and animals get sick. We will be better able to help farmers understand the importance of vaccinating their birds against Newcastle disease if we first understand what farmers believe about why their birds get sick.

Do not discredit local ideas and beliefs. Traditional beliefs have been built up over time and generally give an explanation as to why disease appears as it does.

Even current scientific theories change with the passage of time as new discoveries are made and certain details are better understood.

Newcastle disease is a viral disease that kills more village chickens than any other disease. It is caused by germs that are too small to be seen with the naked eye without special equipment.

The germ that causes Newcastle disease is called the Newcastle disease virus. When the virus enters the body of a bird, the bird does not get sick immediately. It takes some days, usually 3 to 5 days (but sometimes 2 to 15 days) for the bird to show signs of illness.

During these first few days when the bird does not look sick, the number of viruses inside its body increases rapidly and some germs can leave the bird when it breathes or drinks or excretes. If other birds come in contact with these germs, then they too will get sick (Figure 1).

The virus causing ND varies in strength[1]. Those with high strength cause many deaths while those with low strength cause few to no deaths.

For example, if a farmer has 10 chickens, then:

Description of Newcastle disease virus	No. of deaths caused
Virus with low strength	1 out of 10 chickens will die
Virus with moderate strength	5 out of 10 chickens will die
Virus with high strength	10 out of 10 chickens will die

3.3 Where does Newcastle disease come from?

The germs that causes Newcastle disease can come into a homestead from the following sources:

- Neighbours: Your neighbour's sick birds can easily pass the disease to chicken in your homestead.
- Visitors and workers who come into your homestead.
- Birds that you buy from the market or from other people that may appear healthy may bring the disease into your homestead.
- Equipment: sharing of contaminated equipment between farms and failure to clean utensils used in chicken husbandry.

[1] Strength is the disease producing power of a germ. A virus with low strength produces a mild form of disease whereas a virus with high strength produces severe form of disease

- Rats, moles, dogs, cats, wild birds, insects or feed that you buy may carry the disease into your homestead.

3.4 How does the disease spread?

There are many ways through which the disease spreads from one bird to another and from one homestead to another (Figure 2). These include:

i) Breathing contaminated air

ii) Drinking contaminated water

iii) Eating contaminated feed

iv) Contact with sick chickens (new purchase, gifts)

v) Contact with contaminated surfaces/products: meat, intestines, egg, feathers

vi) Contact with people (can be carried by shoes, clothes etc)

vii) Contact with parts of infected chickens (egg shell, feathers etc)

viii) Contaminated chicken house

ix) Contaminated cars, hoes, cages and baskets

x) Carrier ducks, turkeys and some wild birds

xi) Carnivorous animals like dogs and cats can help in the spread of the virus when they drag dead chickens

xii) Sharing of equipment of diseased chickens with healthy chickens

How the disease spreads

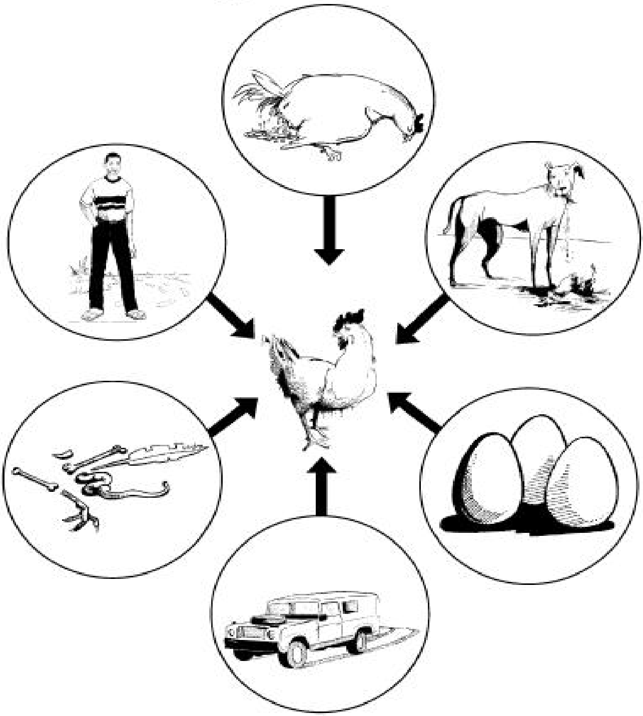

Figure 2. How Newcastle disease spreads

3.5 Treatment of Newcastle disease

Newcastle disease has no cure; chickens that are already sick cannot be treated

3.6 Prevention of Newcastle disease

The only effective means of preventing Newcastle disease is by vaccinating the chickens

3.7 Assessment Questions

1. What are some of the symptoms of Newcastle Disease?
2. What is the main cause of Newcastle Disease?
3. How can you as a farmer control the spread of Newcastle Disease?

SESSION 3

4. Vaccines and vaccination

4.1 Introduction

In this session, we will learn what vaccines are and the importance of vaccinating chickens. We will go ahead to explain how vaccines work. Due to the crucial role vaccines perform in chicken rearing, we will further explain the proper ways of handling and transporting vaccines for use by farmers. We will also emphasise why farmers should take note of the important information in the vaccine packages.

4.1.1 Session learning outcomes

By the end of this session, the learner will be able to:

i) Distinguish between a vaccine and vaccination

ii) Explain the importance of vaccinating chicken

iii) Describe how vaccines work in chicken

iv) Identify important information in the packaging of vaccines

v) Describe the proper ways of handling vaccines.

4.1.2 Time required

This session should take one and half hours.

4.1.3 Materials/preparation needed

Slides, charts, real objects (dropper or vial of a vaccine)

4.1.4 Training methods

Lecture method, demonstrations, practical

Facilitator's guide

The facilitator should ask the trainees to share their knowledge and experiences about vaccines and vaccination of poultry. Ask trainees if they vaccinate their chicken, who does it for them, where they get the vaccines, if they think that vaccines are effective. Show farmers a sample of a vaccine vial and help them read information provided on the vaccine label. Show then the materials – syringes, needles, droppers. Demonstrate and practice with trainees how to reconstitute vaccines

4.2 What are vaccines?

- These are medicines that are given to animals and humans to protect them from getting a disease before the disease strikes.
- They are used to prevent diseases from affecting the animals.
- Vaccines **DO NOT** treat or cure the disease and therefore should not be given to animals that are sick.
- Vaccines are different from other medicines which are used to treat diseases.
- A vaccine only protects an animal against a specific disease the vaccine is made for.
- For vaccines to work well, they must be stored, mixed, and applied appropriately in the right quantities.
- Vaccines work better in animals that are healthy, clean and well fed.
- Vaccine may not be able to protect an animal well if it is not well fed or lives in a dirty place.

4.3 Importance of vaccinating chicken

- Vaccinating chickens after every three months prevents most chicken from getting, Newcastle disease.
- It prevents the spread of the diseases to your neighbours.
- Vaccines prevent spread of diseases from the hen to chicks through the eggs.

4.4 How do vaccines work?

If Newcastle disease germs get into a chicken, it<br><br>will get sick after about a day or two. The chicken may die or if its lucky and strong, it will get well.<br><br>Its' body then develops a defence called "immunity". Immunity is like soldiers who protect people from an enemy	**Figure 1(a). Germs get into the body of chicken making chicken sick.**<br>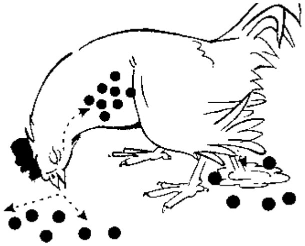
This immunity (soldiers) fights against the virus and the chicken gets better. This immunity does not happen immediately. It takes about a week after the sickness starts for the body to create this immunity.	**Figure 1(b). Soldiers fight germs in the body of chicken**<br>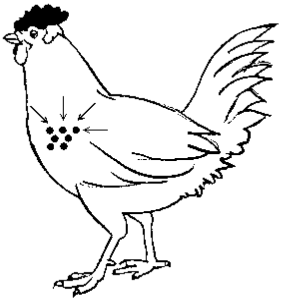

The immunity remains in the body of the chicken after all the germs are destroyed.	**Figure 1(c). Soldiers remains in the body of chicken** 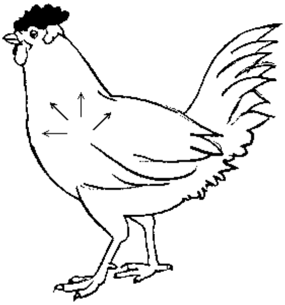
If this same chicken is later attacked by Newcastle disease, the body's immunity is ready and destroys the germs before it can multiply. The chicken is protected from the disease. It does not become sick.	**Figure 1(d). The soldiers in the body chicken are ready to fight germs** 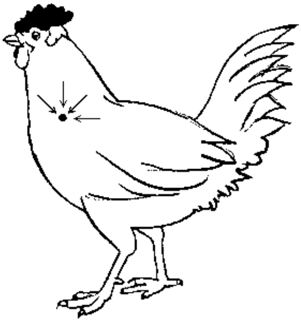
The Newcastle disease vaccine works in a similar way as in the example above. The vaccine makes the body of the chicken to develop immunity (soldiers) without making the chicken sick. The vaccine is given before the chicken is attacked by germs	**Figure 1(e). Vaccines also produce soldiiers in the body of chicken** 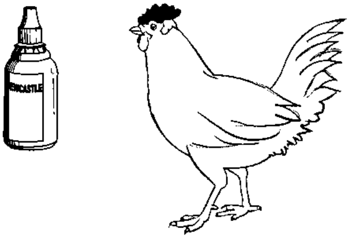

Immunity (soldiers) takes about a week to develop. Vaccine makes the chicken a strong and therefore cannot be attacked by the germs	**Figure 1(f). Vaccines make bird strong and cannot be attached by germs** 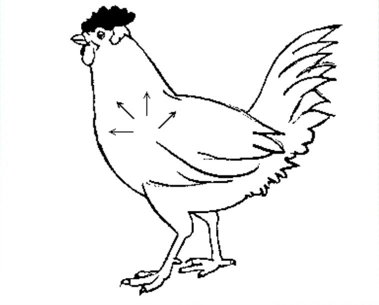

4.5 How vaccines are packed

Vaccines are packed in small plastic or glass bottles called vials. They come in either liquid or pellet form. The vaccine vial and label are very important. The label contains important information that will help you know the following things about the vaccine;

i. Expiry date: do not buy expired vaccines

ii. Manufacturer's name

iii. Name of the disease the vaccine will prevent

iv. Type/strain of vaccines (show farmers different types of strains)

v. Doses contained in the vial

vi. How the vaccine should be stored/preserved?

vii. Batch number

viii. How the vaccine should be prepared/reconstituted and route of administration

ix. Livestock species the vaccine should be used on

4.6 Number of doses per vial of vaccine

- Chicken vaccines are usually sold in vials of different doses.
- In Kenya, NCD vaccine is sold in vials of: 25 doses, 100 doses, 200 doses, 1000 doses
- The cost of vaccine arises from the cost of the vial, metal cap, rubber stopper, and label, while the vaccine itself is relatively cheap. Therefore, the cost of a dose is cheaper in a higher dose vial than in a lower dose vial.

4.7 Vaccine handling and storage

- Vaccines should be stored in a refrigerator at temperatures of between 4°C and 8°C.
- Vaccines should not be kept in a freezer.
- They should not be kept under direct sunlight and heat.
- Keep vaccines away from children (children should not play with vaccines)
- However, there are general principles of handling all types of vaccines.

4.8 Transporting vaccines

Vaccines can be transported from point of sale/source to point of use using the following methods:

- Cool boxes with ice packs
- Thermos flasks with ice packs for short distances
- Containers with ice carefully wrapped with damp cloth and protected from direct sunlight

4.9 Assessment activity/questions

1. Distribute one vial of the vaccine to each participant. Ask each participant to check that they can locate all the information presented during the session on the label.

2. What are some of the recommended guidelines for handling or transporting vaccines?

3. Why is it very important to vaccinate your chickens?

SESSION 4

5.1 Administration Newcastle disease vaccine

5.1 Introduction

This session explores the methods that community vaccinators can use to administer Newcastle disease vaccine to chickens. Trainees are taken through what is required in each of the methods of administration. The session also focuses on how to use and maintain the different materials as plastic droppers, syringes and needles used in the process of vaccination.

5.1.1 Session learning outcomes

By the end of this session, the learner should be able to:

i) Distinguish between the different methods for vaccinating chicken against Newcastle disease.

ii) Outline the directions for eye/nasal administration of a vaccine.

iii) Describe how to use a syringe/needle for vaccination.

iv) Explain how to maintain plastic droppers to ensure long life.

v) Describe the process of administering a vaccine through water.

5.1.2 Time required

This session should take one and half hours.

5.1.3 Materials/preparation needed

Slides, flipchart, photographs, real objects (dropper/syringe).

5.1.4 Training methods

Lecture method, demonstrations, experiential learning, group discussions.

Facilitator's guide

This session is best delivered through practicals. The facilitator should organise a practical session to show trainees materials used in vaccine reconstitution and how they work; how vaccines are reconstituted and administrated to chickens. Show trainees' different brands of vaccines in the market. Emphasize that different vaccines may have different instructions for use. Provide sufficient time to trainees to learn these new skills.

5.2 Reconstitution of Newcastle disease vaccine

Newcastle disease vaccine comes in two forms:

1. Pellet form (freeze-dried form)
2. Liquid form

Vaccines that come in pellet form are mixed with clean water to make a solution that is administered to chickens. Vaccines should be mixed/ reconstituted according to the manufacturer's recommendations. The vaccine solution should be mixed well. Freeze-dried vaccines sometimes come to diluents, water for mixing the vaccine.

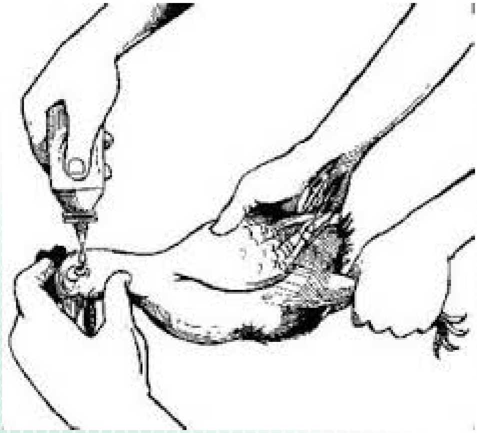

The Newcastle disease vaccine can be administered in two main ways:

1. As eye or nasal drops
2. In drinking water

5.3 Eye drop or nasal drop vaccines

- In order to achieve a successful vaccination through the eye/nasal drop, the following are required: hypodermic needles, syringes, or droppers.
- Eye drop vaccination produces stronger immunity compared to other routes of vaccination.
- Chicken may only be vaccinated three times in a year.

Advantages:

Each chicken gets vaccinated, acquires stronger immunity; relatively less costly as chickens are vaccinated three times per year.

Disadvantage

It is labour intensive and tiring when the flock size is large

5.3.1 How to use a syringe and needle?

- A syringe is used to measure the amount of water or diluent used to mix with the vaccine.
- The markings (divisions) on the syringe are equal to a set volume e.g. the markings in a 10 ml syringe each represent 1 ml.
- Needles have plastic ends for attaching the needle to the syringe and withdrawing diluent or vaccine into the syringe.

5.3.2 Eye dropper

- Eye droppers produce drops of equal size when the droppers are held with the tip end pointing straight down and squeezed gently.
- Eye droppers are made of flexible plastics, with a removable tip.
- A good eye dropper should:
    i. Hold a suitable volume of vaccine
    ii. Preserve the integrity of the vaccine
    iii. Deliver drops of uniform size
- Calibrate the droppers by transferring the mixed vaccine solution from the dropper into a syringe while counting the drops (practice)

N/B: use of human eye droppers is not recommended in chickens as they produce large drops compared to the size of chicken eye which leads to splashing of the drops and wastage of vaccines.

5.3.3 Directions for carrying out nasal/eye drop vaccination

- Before use, ensure the vaccine is indicated for eye or nasal drop administration.
- Vaccines indicated for other routes of administration must never be used as eye drop or nasal drop.
- Open the vaccine vial by removing the aluminium seal.
- Open the bottle with the diluent. **Before reconstituting the vaccine, ensure that the diluent is cooled to temperatures of between 4°C and 8°C**.
- Firmly attach the needle to the syringe and take off the cap from the needle.

- Using the needle fixed on a syringe, withdraw some diluent and Inject into the vaccine vial in the middle of the rubber stopper and mix gently (if vacuum is still intact, it will pull some of the liquid into the vial without any pressure).
- Withdraw the reconstituted vaccine and transfer into the diluent vial and mix well (by gently inverting the bottle). **Do not shake vigorously to form bubbles.**
- The vaccine is now ready to use.

5.3.4 Handling chickens for vaccination

- You can hold the chickens by yourself or have somebody else hold then for you as you vaccinate. Vaccination would be faster if someone held the chicken for you.
- If restraining the chicken by yourself, hold the chicken upright or horizontal.
- Hold the legs together with one hand while keeping the wings down with the other hand (or hold the chicken against your body while keeping one wing down).
- If working with somebody, let the person hold the chicken for you as you vaccinate
- Let the person hold the chicken on its side (by holding the legs and wings). The vaccinator then holds the head while applying the eye/nasal drops.
- By holding the head, you will be able to control the application of the drop into the chicken's eye.

5.4 How not to handle chickens

Avoid the following when handling chickens:

- ❖ Do not hold the chicken upside down by their feet
- ❖ Do not hold the chickens by the base of their wings
- ❖ Do not hold the chickens by their head or neck

5.5 Vaccinating chicken

- ❖ Using a syringe or a dropper, apply one drop of the vaccine on the eye or nostril of chicken. Note that the number of drops will vary depending on the manufacturer's instructions. Vaccinators are encouraged to carefully read and follow the manufacturer's instructions.
- ❖ Vaccine will only be effective if the drop placed on the eye or nostril is absorbed. To achieve this, wait for about 10 seconds after administering the drop before releasing the chicken.
- ❖ If the drop is not completely absorbed, apply another drop on the eye or nostril.
- ❖ Reconstituted vaccines must remain cool as much as possible. To minimize the contents of the vial from getting warm on the hands of the vaccinator, divide the reconstituted vaccine into two or three empty vials and alternate their use while keeping the others in a cool box with ice packs.
- ❖ Vaccination should be done in a cool area under a shade away from direct sunlight.

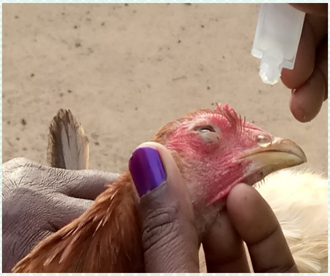

Placing nasal droplet

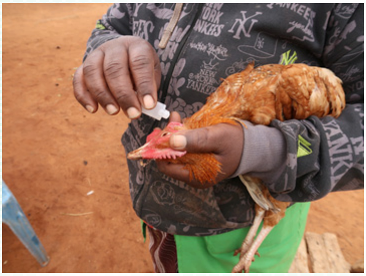

Placing eye droplet

5.6 How to take care of the plastic droppers to ensure long life

- Do not let the vaccine to dry out in the dropper.
- Clean the dropper with clean non-treated water. Do not use treated tap water, or water with soap or disinfectant (will kill the virus).
- Do not clean the tip of the dropper with anything that can damage it.
- Do not force anything through the tip of the dropper that can enlarge its opening.
- Allow the eye dropper to dry well, wrap with a piece of cloth and store away from direct sunlight, heat, rats or mice.

Table 1. Comparison of different routes of giving thermostable Newcastle disease vaccine

Route of giving vaccine	No. of applications per year	Degree of protection
Eye/nasal drop	3	High (80%)
Drinking water	5	Moderate (60%)
Food	8-12	Low (50%)

5.7 Water vaccination (in drinking water)

- Some vaccines are given in the drinking water of the chickens.
- The amount of water consumed per chicken is important in calculating the amount of water to mix the vaccine with.
- This is estimated based on the breed of chicken, age and ambient (area) temperature.

5.7.1 Direction for use of vaccines in drinking water administration of vaccine

- ❖ Do not open or mix the vaccine until it is ready for use
- ❖ Remove all medication, sanitizers and disinfectants from the drinking water 48-72 hours prior to vaccination and up to 24 hours after vaccination (48 hours for antimicrobials).
- ❖ Provide sufficient waterers/drinkers so that all the chickens can drink at once. Thoroughly clean and rinse all the waterers before use.
- ❖ Withdraw water from the chickens for 1 to 2 hours prior to vaccination to stimulate thirst (30-60 minutes in hot climates; 60-90 minutes in cool climates).
- ❖ Estimate the average daily water intake of the chicken then calculate 30% of the volume of water to prepare the vaccine.
- ❖ Place the drinking water in a clean container
- ❖ Open the vaccine vial by removing the aluminum seal and rubber stopper.
- ❖ Take some water from the clean container and fill the vaccine vial two-thirds full and mix gently
- ❖ Mix the reconstituted vaccine with the drinking water in the container and stir gently
- ❖ Rinse the vaccine vial three times to remove all vaccines.
- ❖ Distribute the vaccine stock among the waterers placed where there is no direct sunlight.
- ❖ Check to ensure all the birds are drinking water, if not, redistribute the drinkers as necessary.
- ❖ The chickens should drink all the vaccine stock within 1-2 hours (never in less than 1). When the vaccine is consumed too fast, other chickens will not have an opportunity to drink.

- ❖ Do not provide any other drinking water until all of the vaccine stock has been depleted.
- ❖ Addition of skimmed milk powder (at the rate of 500g/200L) to the water 20-30 minutes before adding the vaccine is recommended to stabilize the water.
- ❖ Use skimmed milk according to the manufacturer's instructions.
- ❖ The number of vaccine vials used in drinking water will depend on the number of chicken to be vaccinated. It is important to establish the number chicken first to avoid under dosing.

5.8 Assessment questions

1. What are some of the requirements of eye/nasal administration of a vaccine?
2. Highlight some of the directions the farmer should pay attention to when carrying out eye/nasal or drinking water administration of the Newcastle disease vaccine.
3. What are some of the advantages of eye/nasal drop vaccines?

SESSION 5

6. General disease control and prevention methods

6.1 Introduction

This session provides general information on the control and prevention of poultry diseases. It explains some of the strategies that trainees can employ to minimise the risk of infections and improve the general health and productivity of their poultry and improve general efficiency in vaccine administration.

6.1.1 Session learning outcomes

By the end of the session, the learner will be able to:

i) Describe the general disease control and prevention methods in chickens

ii) Explain strategies a chicken farmer should use in ensuring quality control of vaccination

iii) Discuss different reasons for vaccination failure.

6.1.2 Time required

This session should take one hour.

6.1.3 Materials/preparation needed

Slides, flipcharts, photographs, demonstrations.

6.1.4 Training methods

Lecture method, group discussions, experiential learning.

6.2 Control and prevention of diseases in chicken

Prevention is better than cure. The following biosecurity measures if adopted, can help reduce the spread of ND in your locality:

- ❖ Avoid buying chickens from the market during a ND outbreak.
- ❖ Disinfect litter material before use and keep it dry at all times.

- ❖ Where possible, separate chicks from adult chickens because adults are likely carriers of several diseases that can easily infect chicks.
- ❖ Separate sick birds from healthy ones as soon as you spot them and call a veterinarian or an animal health assistant to examine your chicken and advice you (ensure sick chickens are isolated and given food and clean drinking water).
- ❖ Do not sell chickens during outbreaks. Slaughter them instead and have them as food before they die.
- ❖ Dispose dead chickens by burning or burying deeply where they cannot be retrieved by dogs.
- ❖ Never feed chickens that die from diseases to dogs as they can spread infections to healthy chickens.
- ❖ Do not introduce chickens into your flock from sources where you cannot verify their health status. If not sure, separate (isolate/quarantine) new chickens for at least 1 month before introducing them into you flock.
- ❖ As much as possible, limit contact between visitors and your chickens.
- ❖ Provide your chickens with clean drinking water at all times.
- ❖ Provide adequate good quality feed to your chicken to enable them build immunity against diseases.
- ❖ Clean your chicken house, feeders and drinkers regularly.
- ❖ Keep away rats, pests and predators from your chicken house
- ❖ Maintain a routine vaccination program suitable for your production objective. Consult a veterinarian for advice.
- ❖ Allow adequate natural light into the chicken house.

6.3 Reasons why vaccination may fail

The following factors can cause vaccines not to offer expected protection:

- Inappropriate route of vaccine administration
- Failure to follow the manufacturer's recommendations on vaccine handling procedures
- Vaccinating sick birds

6.4 Assessment questions

1. What are some of the guidelines for the control and prevention of diseases in chickens?
2. What are some of the causes of vaccine failure in chicken?

SESSION 6

7. Participating in chicken vaccination campaigns and keeping records

7.1 Introduction

This session focuses on the importance of record keeping and preparations for participation in chicken vaccination campaigns.

Session learning outcomes

By the end of this lesson, the learner will be able to:

i) Describe importance of keeping vaccination records

ii) Understand how well to prepare for chicken vaccination campaign.

iii) Understand the importance of targeting both women and men for the vaccination exercise

7.1.1 Time required

This session should take one and half hours

7.1.2 Materials/preparation needed

Slides, flipchart, photographs

7.1.3 Training methods

Lecture method, group discussion, demonstrations

7.2 Participating in a chicken vaccination campaign

7.2.1 What is a chicken vaccination campaign?

This is a vaccine delivery strategy that seeks to vaccinate large numbers of chicken in a given locality within a short time so as to attain herd immunity levels - a proportion of immune chicken in a given population/locality/area above which a disease may no longer spread vigorously. The campaigns are in most cases sponsored by government,

development agents, projects or programs. For smallholder chicken vaccination campaign, this is a door to door service that is offered early in the morning before the birds are let out or late in the evening when they come back into their pens for overnight stay.

7.2.2 Participating in a chicken vaccination campaign

Preparation is key to successful roll out of a chicken vaccination campaign

- ❖ Community vaccinators should prepare well to participate in chicken vaccination.
- ❖ Re-schedule your daily activities to ensure that they do not interfere with the vaccination exercise.
- ❖ Arrive at the vaccine collection site before the start of the activity each day.
- ❖ Before leaving the vaccine collection site, check your vaccine tool kit to ensure it has all materials and vaccine that you need for the day.
- ❖ Where vaccination is done on a house to house basis, let the farmers know in advance the date and time when their chicken will be vaccinated. Encourage them to confine the birds for vaccination.
- ❖ Most farmers have multi-age chicken and different species raised in the same household. Ensure that you vaccinate all birds including chicks (more than 2-weeks old). Failure to vaccinate them greatly reduces vaccination coverage and threaten the objectives of the vaccination campaign.
- ❖ Thoroughly wash your hands with soap and water at the first instance before the start of the vaccination and in between the households to minimise the risk of disease transmission.
- ❖ Before vaccinating, observe the chickens and ask the farmers if they have noticed any sick birds. If you see any sick birds,

consult with the team leader or supervisor for advice and direction. **Do not vaccinate sick birds.**

- ❖ Capture all the records legibly in the prescribed format at the end of the vaccination before moving to the next household.
- ❖ At the end of each vaccination day, safely dispose all the vaccination waste according to the prescribed guidelines.

7.3 Record keeping for community vaccinators

Community vaccinators should accurately capture all relevant information on all their vaccination activities and present them to their supervisors. Generally, these records will include

- ❖ Date of vaccination
- ❖ Farmer's name, gender and telephone number
- ❖ Name of locality
- ❖ Total number of chickens owned by farming household
- ❖ Number of chickens vaccinated
- ❖ Any other information that may be required

7.4 Vaccine regimes for local chickens but also applicable to indigenous chickens

Vaccination schedule for local chicken		
**Day**	**Vaccine**	**Method**
1	Marek's	I/M injection
10-14	Gumboro intermediate	D/W
15-18	NCD+IB Live	Eye drop
24-28	Gumboro intermediate plus	D/W

28-32	NCD+IB Live	Eye drop/drinking water
Week 6-8	NCD killed or	I/M
	NCD+IB live	D/W
	Fowl typhoid	I/M
Week 8-10	Fowl pox	Wing web stab
Week 12-14	Fowl typhoid	I/M
Week 16-18	NCD+IB Live	D/W or spray
	Fowl cholera	Subcutaneous injection
Note: Local chickens should be vaccinated at least three (3) times per year against Newcastle disease (every four months)		

7.5 Importance of targeting both women and men farmers for the vaccination

1. Men and women have different roles and responsibilities when it comes to livestock rearing hence it is important to ensure that both gender including youth are trained.
2. Women are sometimes the ones left home while men migrate to the urban areas hence if a training targets the household heads who are mostly men, such women may be missed out.
3. The times when women and men are available may be different hence it is important to understand the general program of men and women in the community to enable schedule the trainings during times that are appropriate for both gender.

7.6 Assessment questions

1. What are some of the details that a community vaccinator needs to capture every time he/she is carrying out vaccination?
2. What are some of the ways in which we can carry out a chicken vaccination campaign?
3. What would you consider if you are planning to carry out mass Newcastle disease vaccination in Makueni county?

4. What are some of the reasons why it is important to target women and men specifically for the trainings?

The University of Nairobi
P.O.Box 30197, GPO, Nairobi,
Kenya
Tel: (+254-20) 491 0000

The Cooperative University of Kenya
P.O. BOX 24814-00502, Karen, Nairobi
– Kenya,
Tel: (020)-2430127, 0724 311606

Kenya Agricultural & Livestock Research Organization
P.O Box 57811-00200, Nairobi, KENYA
Tel. No(s): +254-722206986/ +254-733333223
Web: www.kalro.org

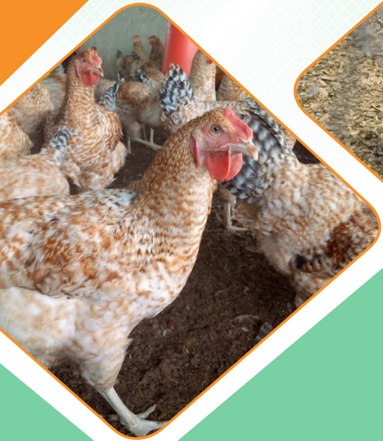

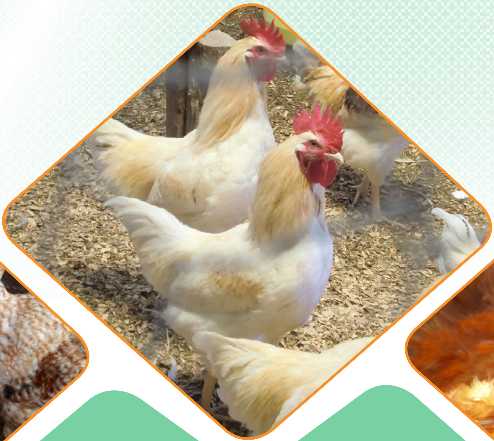

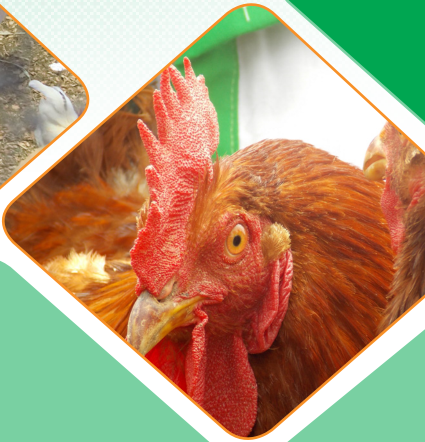

Supplement: S2 Appendix — (PDF) [file pone.0308088.s002.pdf]
