# Supplementary material for: Effectiveness of a community-centered Newcastle disease vaccine delivery model under paid and free vaccination frameworks in southeastern Kenya
Source: PLoS One. 2024 Aug 1;19(8):e0308088. doi: 10.1371/journal.pone.0308088 (PMC11293705; doi:10.1371/journal.pone.0308088)
Supplement: S3 Appendix — (PDF) [file pone.0308088.s003.pdf]

Vaccine storage and handling practices

Proper vaccine storage and handling are important factors in preventing and eradicating many common vaccine preventable diseases. Yet, each year, storage and handling errors result in revaccination of many patients and significant financial loss due to wasted vaccines. Failure to store and handle vaccines properly can reduce vaccine potency, resulting in inadequate immune responses in patients and poor protection against disease.

Vaccine cold chain

Vaccines must be stored properly from the time they are manufactured until they are administered. Potency is reduced every time a vaccine is exposed to an improper condition. This includes overexposure to heat, freezing cold, or light at any step in the cold chain. Once lost, potency cannot be restored.

Staff training

Vaccine storage and handling practices are only as effective as the staff that implements them. Staff that is well trained in general storage and handling principles and organization-specific storage and handling standard operating procedures (SOPs) is critical to ensuring vaccine supply potency and patient safety.

1. Primary vaccine coordinator

This person will be responsible for ensuring all vaccines are stored and handled correctly. Coordinator responsibilities should include:

a) Ordering vaccines
b) Overseeing proper receipt and storage of vaccine deliveries
c) Documenting vaccine inventory information
d) Organizing vaccines within storage units
e) Setting up temperature monitoring devices
f) Checking and recording minimum/maximum temperatures at start of each workday
g) Reviewing and analyzing temperature data at least weekly for any shifts in temperature trends
h) Rotating stock at least weekly so vaccines with the earliest expiration dates are used first
i) Removing expired vaccine from storage units
j) Responding to temperature excursions (out-of-range temperatures)
k) Maintaining all documentation, such as inventory and temperature logs
l) Monitoring operation of vaccine storage equipment and systems
m) Overseeing emergency preparations
n) Ensuring appropriate handling of vaccines during a disaster or power outage

Vaccine Storage and Temperature Monitoring Equipment

1. Storage Unit Placement

Good air circulation around the outside of the storage unit is important. Place a storage unit in a well-ventilated room, leaving space between the unit, ceiling, and any wall. Nothing should block the cover of the motor compartment. The unit should be firm and level, with the bottom of the unit above the floor. Make sure the unit door opens and closes smoothly and fits squarely against the body of the unit. If not secured properly, unit doors pose a particular risk to maintaining appropriate internal temperatures of vaccine storage units. Studies find most units work best when placed in an area with standard indoor room temperatures, usually between 20° C and 25° C (68° F and 77° F). Check the manufacturer-supplied owner’s manual for additional guidance on placement and spacing.

2. Stabilizing Temperatures in New and Repaired Units

It may take two to seven days to stabilize the temperature in a newly installed or repaired refrigerator and two to three days for a freezer. Before using a unit for vaccine storage, check and record the minimum and maximum temperatures each workday for two to seven days. If temperatures cannot be recorded digitally, check and record temperatures a minimum of two times each workday. Once you have two consecutive days of temperatures recorded within the recommended range, your unit is stable and ready for use.

3. Temperature Ranges

Refrigerators should maintain temperatures between 2° C and 8° C (36° F and 46° F). Freezers should maintain temperatures between -50° C and -15° C (-58° F and +5° F). Refrigerator or freezer thermostats should be set at the factory set or midpoint temperature, which will decrease the likelihood of temperature excursions. Consult the owner’s manual for instructions on how to operate the thermostat. Thermostats are marked in various ways and, in general, show levels of coldness rather than temperatures. The only way to know the temperature where vaccines are stored is to measure and monitor it with a temperature monitoring device.

4. Temperature Monitoring Device (TMD)

Every vaccine storage unit must have a TMD. An accurate temperature history that reflects actual vaccine temperatures is critical for protecting your vaccines. Investing in a reliable device is less expensive than replacing vaccines wasted due to the loss of potency that comes from storage at out-of-range temperatures. Have at least one backup TMD in case a primary device breaks or malfunctions.

5. Monitoring Vaccine Temperature and Vaccine Equipment

Monitoring vaccine storage equipment and temperatures are daily responsibilities to ensure the viability of your vaccine stock and the safety of your chickens. Implementing routine monitoring activities can help you identify temperature excursions quickly and take immediate action to correct them, preventing loss of vaccines and the potential need for revaccination of chickens.

6. Power Supply

Even with appropriate equipment and temperature monitoring practices in place, power disruption can result in destruction of the entire vaccine stock. The following precautions should always be taken to protect the storage unit's power supply.

a) Plug in only one storage unit per electrical outlet to avoid creating a fire hazard or triggering a safety switch that turns the power off
b) Use a safety-lock plug or an outlet cover to prevent the unit from being unplugged
c) Post "**DO NOT UNPLUG**" warning signs at outlets and on storage units to alert staff, custodians, electricians, and other workers not to unplug units
d) Label fuses and circuit breakers to alert people not to turn off power to a storage unit
e) Use caution when using power outlets that can be tripped or switched off

7. Organizing and Storing Vaccine

Correctly organizing and placing vaccines in a storage unit helps prevent conditions that could reduce vaccine potency or cause vaccine failure.

a) Store vaccines in their original packaging with lids closed until ready for administration. Vials should always be stored in their original packaging.
b) Check and record storage unit minimum and maximum temperatures at the start of each workday. If your TMD does not read min/max temperatures, then check and record the current temperature a minimum of 2 times per workday (at the start and end of the workday).
c) Store each type of vaccine or diluent in its original packaging and in a separate container.
d) Position vaccines and diluents two to three inches from the unit walls, ceiling, floor, and door. If using a household grade unit, avoid storing vaccines and diluents in any part of the unit that may not provide stable temperatures or sufficient air flow, such as directly under cooling vents, in deli, fruit, or vegetable drawers, or on refrigerator door shelves.

The instability of temperatures and air flow in these areas may expose vaccines to inappropriate storage temperatures.

e) Label shelves and containers to clearly identify where each type of vaccine and diluent is stored

f) Store vaccines and diluents with similar packaging or names on different shelves.

g) Whenever possible, store diluent with the corresponding refrigerated vaccine. Never store diluent in a freezer

h) Avoid placing or storing any items other than vaccines, diluents, and water bottles inside storage units

i) If other medications and biological products must be stored in the same unit as vaccines, they must be clearly marked and stored in separate containers or bins from vaccines

j) Potentially contaminated items (e.g., blood, urine, stool) should be properly contained and stored below vaccines due to risk of contamination from drips or leaks

k) The freezer of a household-grade unit may be used for non-vaccine, medical storage, so long as the use does not compromise the temperature range within the refrigerator compartment where vaccine is stored

l) Arrange vaccines and diluents in rows and allow space between them to promote air circulation

m) Place vaccines and diluents with the earliest expiration dates in front of those with later expiration dates.

Record the following:

- Minimum/maximum temperature
- Date
- Time
- Name of person who checked and recorded the temperature
- Any actions taken if a temperature excursion occurred
- If a reading is missed, leave a blank entry in the log.

8. Temperature Excursions

Temperature excursions or inappropriate storage conditions for any vaccine require immediate action. Any temperature reading outside the recommended ranges in the manufacturers' package inserts is considered a temperature excursion. In general, manufacturers analyze information about the magnitude of the temperature excursion and the total amount of time that temperatures were out of range, as well as information about the vaccine in question, to determine whether a vaccine is likely to still be viable.

In case of temperature excursion:

- ❖Any staff who hears an alarm or notices a temperature excursion on the DDL should notify the primary or alternate vaccine coordinator immediately or report the problem to their supervisor
- ❖Notify staff by labeling exposed vaccines "DO NOT USE" and placing them in a separate container apart from other vaccines (do not discard these vaccines)
- ❖Place water bottles on the top shelf and floor and in the door racks. Putting water bottles in the unit can help maintain stable temperatures caused by frequently opening and closing unit doors or a power failure.

9. Other things to consider

1. Freezer compartment to be checked to ensure its working properly and ice packs are available
2. The doors of the refrigerators should be closed at all times unless when removing vaccines or checking temperature
3. Vaccines should be stored under refrigeration conditions 5°C (+2°c to +8°c), **DO NOT** store vaccines in the freezer
4. In case of power outage, switch on the back-up generator within XX hours
5. Do not store the vaccines closer to the door, walls or on the door compartments of the refrigerator
6. Only remove vaccine vials that are about to be issued to farmers or community vaccinators
7. Encourage the community vaccinators to come with thermos flasks but farmers can be issued with vaccines wrapped in foils and newspapers
8. Vaccines should be issued between 8 am and 5 pm
9. Vaccine sales to community vaccinators/farmers shall be restricted to 2 vials per community vaccinator/farmer per day
