# Supplementary material for: Effectiveness of a community-centered Newcastle disease vaccine delivery model under paid and free vaccination frameworks in southeastern Kenya
Source: PLoS One. 2024 Aug 1;19(8):e0308088. doi: 10.1371/journal.pone.0308088 (PMC11293705; doi:10.1371/journal.pone.0308088)
Supplement: S4 Appendix — (PDF) [file pone.0308088.s004.pdf]

Data capture sheet for community vaccinators

Name of chicken vaccinator……………………………………		Mobile phone No.				Date:		Time started: ……………………………….<br><br>Time ended: ………………………………..			
Name of household head	Phone contact of household head	Belongs to intervention group<br>1. Yes<br>2. No	No. of chicken kept	No. of chickens vaccinated	Reason for not vaccinating if any<br>1.Not at home<br>2. No prepared<br>3. Declined<br>4. Others (specify)	Route of application used<br>1. Eye<br>2. Nasal<br>3. Water	Amount charged for vaccination	Health status of flock<br>1.All are healthy<br>2.Some are sick	Other poultry species kept<br>1.Duck<br>2.Geese<br>3.Turkey<br>4.Dove<br>5.Others	No. of goats kept	No. of sheep kept
Any other comment:											
