# Supplementary material for: Effectiveness of a community-centered Newcastle disease vaccine delivery model under paid and free vaccination frameworks in southeastern Kenya
Source: PLoS One. 2024 Aug 1;19(8):e0308088. doi: 10.1371/journal.pone.0308088 (PMC11293705; doi:10.1371/journal.pone.0308088)
Supplement: S5 Appendix — (PDF) [file pone.0308088.s005.pdf]

GENDER INCLUSIVE VACCINE ECOSYSTEM – ENHANCING DISTRIBUTION AND DELIVERY SYSTEM FOR NEWCASTLE DISEASE (NCD) AND CONTAGIOUS CAPRINE PLEUROPNEUMONIA (CCPP) VACCINE AMONG SMALL HOLDER FARMERS IN MAKUENI

Form for assessing performance of community vaccinators

Ward……………………………………… **Village: ……………………………………**

Name of Community Vaccinator……………………………………………………………….

1.	Community vaccinators are transporting vaccines properly (not exposed to sunlight and heat)		
2.	Vaccines not exposed to sunlight during reconstitution and/or administration		
3.	Community vaccinators are reconstituting vaccines properly		
4.	Community vaccinators are administering vaccines properly in the eye or nostril		
5.	Community vaccinators take accurate and legible records		
6.	Community vaccinators treat the farmers with respect when they visit their homesteads		
7.	Community vaccinators are picking vaccines in good time (6.30 -7.30 am)		
8.	Community vaccinators are finishing the job early before the ice melts (before 12 pm)		
9.	Target of 100 chickens per day per CV is being met		
10.	Community vaccinators are providing poultry husbandry information and advice to farmers		
