# Supplementary material for: Effectiveness of a community-centered Newcastle disease vaccine delivery model under paid and free vaccination frameworks in southeastern Kenya
Source: PLoS One. 2024 Aug 1;19(8):e0308088. doi: 10.1371/journal.pone.0308088 (PMC11293705; doi:10.1371/journal.pone.0308088)
Supplement: S6 Appendix — (PDF) [file pone.0308088.s006.pdf]

Community Vaccinator Profiles

1. Name of community vaccinator: ………………………..

2. Sex: [1] Female [2] Male

3. Marital status of community vaccinator;

   [1] Single [2] Married [3] Separated [4] Divorced [4] Widowed

4. What is the age of community vaccinator? ……………………………………………….

5. Type of Household: [1] Female-headed household [2] Male-headed household

6. Household size? …………….........…………………………………………………

7. How many children are 5 years and below? ....

8. What is your highest level of Education?

**No.**	**Level of education**	**Years spent**
1.	No formal education	
2.	Primary	
.03.	Secondary	
4.	College	
5.	University	
6.	Vocational training	

9. What is your source of income?

   [1] Farming [2] Salaried employment [3] Informal employment [4] Business

   [5] Others specify

10. What is your Income range per month (ksh)?

   [1] <3,000 [2] 3,000-6,000 [3] 6,001-9,000 [4] 9,001-12, 000 [5] >12000

11. Does your household own the following modes of transport? (Tick all that apply)

[1] Bicycle [2] Motorcycle

11a. IF YES, are you able to use it (them) whenever you want?

[a] Yes [b] No

12. Did you use any of the following modes of transport during the vaccination exercise?

No.	Means of transport	Tick all that apply
1.	Household bicycle	
2.	Borrowed bicycle	
3.	Household motorcycle	
4.	Borrowed motorcycle	
5.	Others specify	

12a. IF used a household motorcycle for transport, ask and tick appropriately if the community vaccinator:

No.	Means of transport	Tick all that apply
1.	Incentivized the husband	
2.	Fuelled the motorcycle and was taken around by the husband	
3.	Fuelled the motorcycle and drove herself	
4.	Self-driving CV had to return the motorcycle after specified hours	
5.	Others specify	
