# Supplementary material for: Effectiveness of a community-centered Newcastle disease vaccine delivery model under paid and free vaccination frameworks in southeastern Kenya
Source: PLoS One. 2024 Aug 1;19(8):e0308088. doi: 10.1371/journal.pone.0308088 (PMC11293705; doi:10.1371/journal.pone.0308088)
Supplement: S7 Appendix — (PDF) [file pone.0308088.s007.pdf]

Assessment forms for smallholder chicken farmers in Makueni County

1. Name of the respondent …………………………………………………………………………………..……….
2. Mobile contact ……………...………………………………. Name of interviewer:……………………………………...
3. Sub-county [1] Makueni [2] Kibwezi West [3] Kibwezi East
4. Ward [1] Kitise [2] Kathonzweni [3] Masongaleni [4] Kambu [5] Makindu [6] Kikumbulyu North
5. Village ……………………..…………………………..

6. Gender of the respondent: **[1]** Male **[2]** Female

7. Age of the respondent; . ……………………………………………………………………………………..

8. Occupation of respondent:
   [1] Farmer
   [2] Formal employment
   [3] Informal employment
   [4] Business
   [5] Remittances
   [6] Others specify

9. How did you hear about this vaccination campaign?

   [1] Through the village elder
   [2] School announcement
   [3] Chief Barazas
   [4] Nyumba Kumi officials
   [5] Posters
   [6] Banners
   [7] Public address announcement
   [8] Church announcement
   [9] Others specify ……………………………………………………………………………………………

10. In your assessment, do you think the information you received was sufficient in helping you to make informed decisions on whether or not to have your chickens vaccinated?
    [1] Yes **[2]** No
11. If No, what other communication methods would you advise the project to use? ………………………………
    ……
    ………..

12. What expectations did you have when you heard about our vaccination campaign?
    [1] My chickens will be vaccinated against Newcastle disease
    [2] my chickens will be vaccinated against all chicken diseases
    [2] I will be given advise on best chicken husbandry practices
    [3] My ducks will be vaccinated against Newcastle disease
    [4] Other animals other than chicken will be vaccinated
    [5] Others specify ……………………………………………………………………………………………

13. To what extent have your expectations been met? (Proportional piling pictorial)
    [1] Not at all
    [2] To a small extent
    [3] To a medium extent
    [4] To a large extent
    [5] All have been met

14. In your own assessment, rate the performance of the community vaccinators in the following areas (tick appropriately)

**Description**	**Yes**	**No**
[1] CV was respectful and professional		
[2] CV communicated vaccination objectives clearly		
[3] CV was well dressed and in work dustcoat		
[4] CV introduced herself/himself properly		
[5] CV answered farmer's questions well		
[6] CV provided advise on other aspects of poultry production		
[7] CV handled the chickens properly		
[8] CV accurately administered the vaccine in the eye/nose		
[9] CV reported at the time he/she had promised		
[10] CV charged/did not charge any money for service rendered		
[11] Others specify ……………………………………………..		

15. Would you allow this community vaccinator to vaccinate your chickens another time?
    [2] Yes [3] May be [4] No

- Explain why ……………………………………………………..……………………………

16. In your opinion, do you think Newcastle disease is an important disease that causes losses to chicken farmers? **[1]** Yes **[2]** No [3] Don't know

17. In your opinion, do you think preventing Newcastle disease through routine vaccination is important?
    [1] Yes **[2]** Maybe [3] No [3] Don't know

18. Will you continue vaccinating your chickens to prevent Newcastle disease even after the project ends?
    [1] Yes **[2]** Maybe [3] No [3] Don't know
