# Supplementary material for: Effectiveness of a community-centered Newcastle disease vaccine delivery model under paid and free vaccination frameworks in southeastern Kenya
Source: PLoS One. 2024 Aug 1;19(8):e0308088. doi: 10.1371/journal.pone.0308088 (PMC11293705; doi:10.1371/journal.pone.0308088)

CONSORT diagram showing the drop out over time of participants through each stage of the intervention

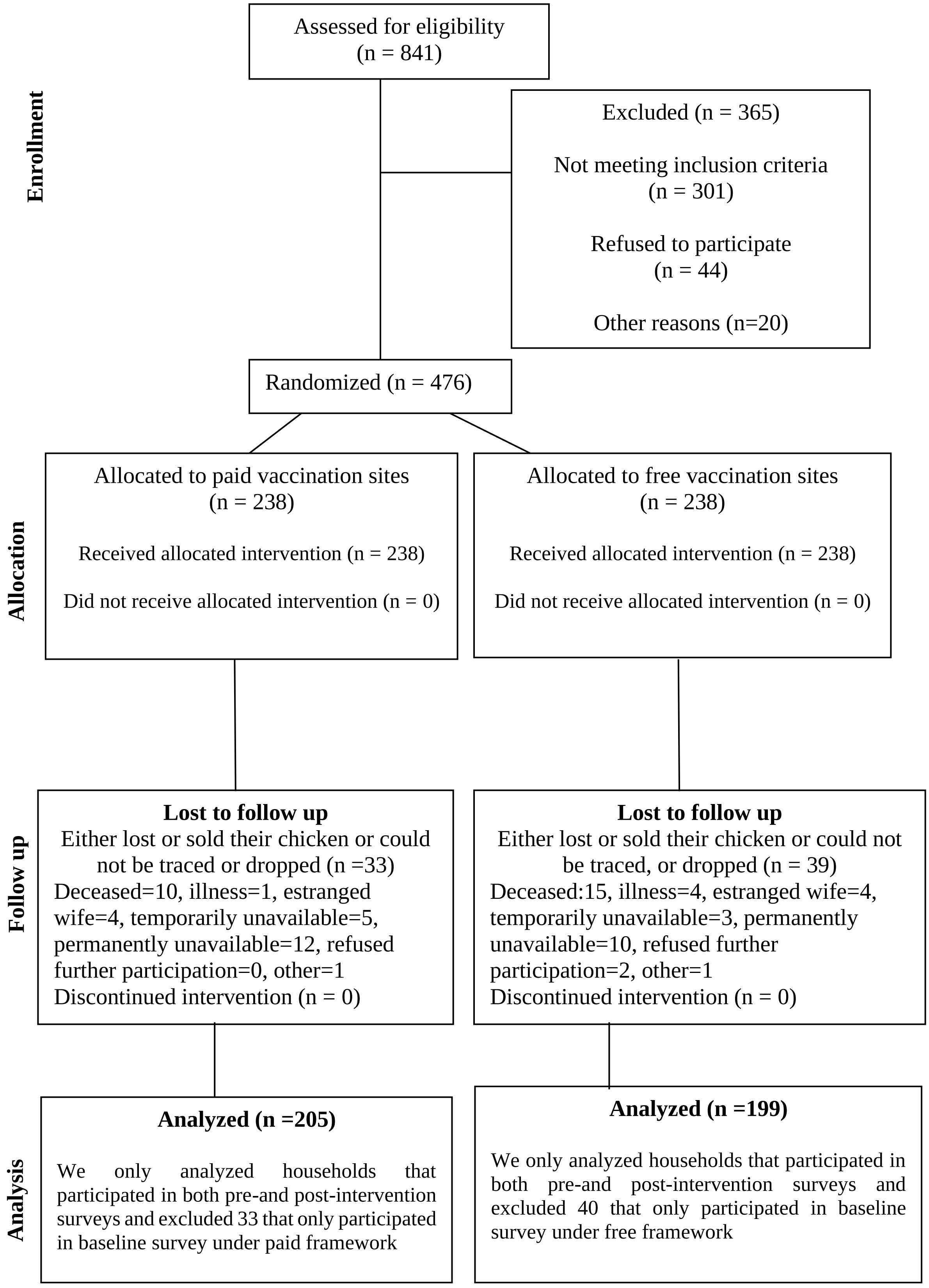

Supplement: S8 Appendix — (PDF) [file pone.0308088.s008.pdf]
