# Supplementary material for: Effectiveness of a community-centered Newcastle disease vaccine delivery model under paid and free vaccination frameworks in southeastern Kenya
Source: PLoS One. 2024 Aug 1;19(8):e0308088. doi: 10.1371/journal.pone.0308088 (PMC11293705; doi:10.1371/journal.pone.0308088)
Supplement: S9 Appendix — (PDF) [file pone.0308088.s009.pdf]

QUESTIONNAIRE FOR ASSESSMENT OF MODEL EFFECTIVENESS

MODULE A. HOUSEHOLD IDENTIFICATION (REQUIRED)

Household Identification	Code		Interview details	Code
**A01**. Household Identification:			**A06**. Start time of interview (hh:mm)	:
**A02**. Village:			**A07**. End time of interview (hh:mm)	:
**A03**. Sub-county:			**A08**. Name/code of enumerator:	
**A04**. District:			**A09**. Date of visit (dd/mm/yyyy):	/ /
**A05**. Cell phone number:				

MODULE B. HOUSEHOLD LISTING AND DEMOGRAPHICS (REQUIRED). *Enumerator: Ask these questions about all household members.*

DRAFT

First, we would like to ask you about each member of your household. Please list the names of everyone considered to be a member of this household, starting with the primary respondent.

ID CODE	Name of household member?<br><br>[start with primary respondent, continue with the secondary respondent, and other members in descending order of age]	What is [NAME's] sex?<br><br>1 = Male<br>2 = Female	What is [NAME's] relationship to the primary respondent?<br><br>**CODE 1**	When was [NAME's] born?<br><br>(Year of birth (YYYY)
	**B01**	**B02**	**B03**	**B04**
1				
2				
3				
4				
5				
6				
7				
8				
9				
10				
11				
12				
13				
14				
15				

Code 1 (B03) Relationship to primary respondent

Primary respondent...............1	Brother/sister………………. 7	Mother/father-in-law……..…………………………………………12
Spouse……………………..........2	Nephew/niece……………………8	Cousin of primary respondent's spouse ……..……………………..13
Son/daughter………..…………3	Nephew/niece of spouse……….9	Other relative…………………..14
Son/daughter-in-law…………...4	Cousin of primary respondent....10	Maid……………………………………………………………...15
Grandson/granddaughter ……5	Brother/sister-in-law……….......11	Other relationship (specify)...16
Mother/Father...…………………..6		

MODULE G1. INDIVIDUAL IDENTIFICATION

**G1.01. HOUSEHOLD IDENTIFICATION:**	☐☐☐☐☐☐	**G1.04 TYPE OF HOUSEHOLD**	MALE AND FEMALE ADULT .... 1<br>FEMALE ADULT ONLY 2
**G1.02. NAME OF RESPONDENT CURRENTLY BEING INTERVIEWED (ID CODE FROM ROSTER IN SECTION B HOUSEHOLD ROSTER):**	☐☐	**G1.05. OUTCOME OF INTERVIEW:**<br><br>**CIRCLE ONE**	COMPLETED .... 1<br>HOUSEHOLD MEMBER TOO ILL TO RESPOND/COGNITIVELY IMPAIRED .... 2<br>RESPONDENT NOT AT HOME/TEMPORARILY UNAVAILABLE .... 3<br>RESPONDENT NOT AT HOME/EXTENDED ABSENCE .... 4<br>REFUSED .... 5<br>COULD NOT LOCATE 6
**SURNAME, OTHER NAME:** ______________________________			
**G1.03. SEX OF RESPONDENT:**<br><br>**G1.03a. WHO ARE YOU INTERVIEWING?**	MALE .... 1<br>FEMALE .... 2<br><br>HUSBAND/MAIN MALE ADULT.........1→ **G1.05**<br>INDEX WOMAN 2	**G1.06. ABILITY TO BE INTERVIEWED ALONE:**<br><br>**CIRCLE ONE**	ALONE .... 1<br>WITH ADULT FEMALES PRESENT .... 2<br>WITH ADULT MALES PRESENT .... 3<br>WITH ADULTS OF BOTH SEX PRESENT .... 4<br>WITH CHILDREN PRESENT .... 5<br>WITH ADULTS OF BOTH SEX AND CHILDREN PRESENT 6

DRAFT

Now I’d like to ask you some questions about your participation in certain types of work activities and on making decisions on various aspects of household life. The questions cover a range of activities, with special emphasis on livestock raising.

QUESTION		RESPONSE
G2.08. Which of the following species and breed type of livestock are raised in your household	CIRCLE ALL APPLICABLE	LARGE RUMINANT (DAIRY – LOCAL BREEDS) …1<br>LARGE RUMINANT (DAIRY - IMPROVED BREEDS) …2<br>LARGE RUMINANT (BEEF OR DUAL PURPOSE – LOCAL BREEDS) …3<br>LARGE RUMINANT (BEEF OR DUAL PURPOSE - IMPROVED BREEDS)…4<br>SMALL RUMINANT (SHEEP, GOAT LOCAL)….5<br>SMALL RUMINANT (SHEEP, GOAT IMPROVED BREEDS)…6<br>POULTRY (LOCAL)….7<br>POULTRY (IMPROVED BREEDS)….8<br>PIGS (LOCAL)…9<br>PIGS (IMPROVED BREEDS)…..10<br>CAMELS….11<br>OTHERS SPECIFY….
G2.09 Please select the one most important species and breed type, for your household livelihood	CIRCLE ONE	LARGE RUMINANT (DAIRY LOCAL) …1<br>LARGE RUMINANT (DAIRY IMPROVED BREEDS) …2<br>LARGE RUMINANT (BEEF OR MIXED LOCAL) …3<br>LARGE RUMINANT (BEEF OR MIXED IMPROVED BREEDS)…4<br>SMALL RUMINANT (SHEEP, GOAT LOCAL)….5<br>SMALL RUMINANT (SHEEP, GOAT IMPROVED BREEDS)…6<br>POULTRY (LOCAL)….7<br>POULTRY (IMPROVED BREEDS)….8<br>PIGS (LOCAL)…9<br>PIGS (IMPROVED BREEDS)…..10<br>CAMELS….11<br>OTHERS SPECIFY….
G2.09a: Reason why it is most important to your household’s livelihood:		
G2.10 Please select the most important species and breed type for your own livelihood	CIRCLE ONE<br><br>**(CAN BE THE SAME AS FOR G3.09)**	LARGE RUMINANT (DAIRY LOCAL) …1<br>LARGE RUMINANT (DAIRY IMPROVED BREEDS) …2<br>LARGE RUMINANT (BEEF OR MIXED LOCAL) …3<br>LARGE RUMINANT (BEEF OR MIXED IMPROVED BREEDS)…4<br>SMALL RUMINANT (SHEEP, GOAT LOCAL)….5<br>SMALL RUMINANT (SHEEP, GOAT IMPROVED BREEDS)…6<br>POULTRY (LOCAL)….7<br>POULTRY (IMPROVED BREEDS)….8<br>PIGS (LOCAL)…9<br>PIGS (IMPROVED BREEDS)…..10<br>CAMELS….11<br>OTHERS SPECIFY….

DRAFT

HOUSEHOLD ID						
RESPONDENT ID						

MODULE G8(A): AUTONOMY IN DECISION-MAKING

		Now I am going to read you some stories about different farmers and their situations regarding different agricultural activities. This question format is different from the rest so take your time in answering. For each I will then ask you how much you are like or not like each of these people. We would like to know if you are completely different from them, similar to them, or somewhere in between. There are no right or wrong answers to these questions.<br><br>**READ ALOUD EACH STORY, SUBSEQUENT QUESTIONs, AND RESPONSE CODES. NAMES SHOULD BE ADOPTED TO LOCAL CONTEXT AND BE MALE/FEMALE DEPENDING ON THE SEX OF THE RESPONDENT. THE ORDER OF TOPICS A-D SHOULD BE RANDOMIZED, AND WITHIN EACH TOPIC, THE ORDER OF STORIES 1-4 SHOULD BE RANDOMIZED.**	Are you like this person?<br><br>**CIRCLE ONE**	Are you completely the same or somewhat the same?<br><br>**CIRCLE ONE**
**STORY**			**G8.01**	**G8.02**
Livestock raising	**B1**	*"[PERSON'S NAME] cannot raise any livestock other than what she has. These are all that do well here."*	YES...1<br>NO.....0 → ***G8.03***	COMPLETELY THE SAME....1 → ***B2***<br>SOMEWHAT THE SAME.......2 → ***B2***
	**B2**	*"[PERSON'S NAME] raises the types of livestock she does because her spouse, or family, or another person or group in her community tell her she must use these breeds. She does what they tell her to do."*	YES...1<br>NO.....0 → ***G8.03***	COMPLETELY THE SAME....1 → ***B3***<br>SOMEWHAT THE SAME.......2 → ***B3***
	**B4**	*"[PERSON'S NAME] chooses the types of livestock that she personally wants to raise and thinks are good for herself and her family. She values raising these types. If she changed her mind, she could act differently."*	YES...1<br>NO.....0 → ***G8.03***	COMPLETELY THE SAME....1 → ***C1***<br>SOMEWHAT THE SAME.......2 → ***C1***
**Livestock Vaccine source:**<br><br>IF GOAT:<br>2. CCPP (Kenya)<br><br>IF CHICKEN:<br>1. NCD	**B5**	*"[PERSON'S NAME] chooses if she wants to personally give [VACCINE] for her [ANIMAL] that she thinks are good for her livestock. She values giving these vaccines. If she changed her mind, she could act differently."*	YES...1<br>NO.....2 → ***G8.03***	COMPLETELY THE SAME....1 → **B1**<br>SOMEWHAT THE SAME.......2 → **B1**
	**B6**	*"[PERSON'S NAME] vaccinates both her own personal animals as well as her neighbors [ANIMAL] against [VACCINES]. She is considered a local expert and is a person that the community asks for help with vaccinating livestock."*	YES...1<br>NO.....2 → ***G8.03***	COMPLETELY THE SAME....1 → **B1**<br>SOMEWHAT THE SAME.......2 → **B1**

DRAFT

	**B7**	*"[PERSON'S NAME] feels that she can access [VACCINE] for her [ANIMAL] easily. She does not consider access to this vaccine as a problem for her livestock.*	YES...1<br>NO.....2 → ***G8.03***	COMPLETELY THE SAME….1 → ***B1***<br>SOMEWHAT THE SAME…....2 → ***B1***

**READ ALOUD EACH STORY, SUBSEQUENT QUESTIONs, AND RESPONSE CODES. NAMES SHOULD BE ADOPTED TO LOCAL CONTEXT AND BE MALE/FEMALE DEPENDING ON THE SEX OF THE RESPONDENT.**			Are you like this person?<br><br>**CIRCLE ONE**	Are you completely the same or somewhat the same?<br><br>**CIRCLE ONE**
**STORY**			**G8.01**	**G8.02**
How to use income generated from agricultural and non-agricultural activities	**D1**	*"There is no alternative to how [PERSON'S NAME] uses her income. How she uses her income is determined by necessity."*	YES...1<br>NO.....0 → ***G8.03***	COMPLETELY THE SAME….1 → ***D2***<br>SOMEWHAT THE SAME…....2 → ***D2***
	**D2**	*"[PERSON'S NAME] uses her income how her spouse, or another person or group in her community tell her she must use it there. She does what they tell her to do."*	YES...1<br>NO.....0 → ***G8.03***	COMPLETELY THE SAME….1 → ***D3***<br>SOMEWHAT THE SAME…....2 → ***D3***
	**D3**	*"[PERSON'S NAME] uses her income in the way that her family or community expect. She wants them to approve of her."*	YES...1<br>NO.....0 → ***G8.03***	COMPLETELY THE SAME….1 → ***D4***<br>SOMEWHAT THE SAME…....2 → ***D4***
	**D4**	*"[PERSON'S NAME] chooses to use her income how she personally wants to, and thinks is best for herself and her family. She values using her income in this way. If she changed her mind, she could act differently."*	YES...1<br>NO.....0 → ***G8.03***	COMPLETELY THE SAME...1→***G8.04***<br>SOMEWHAT THE SAME….2 →***G8.04***

DRAFT

VACCINE WELI MODULE

(Based on Theory of Change and M&E's)

1. Access to Vaccines
2. Barriers to Vaccine Purchase (demand and regulatory)

IF PARTICIPANT OWNS CHICKEN, ASK:

1. NCD

**Question:** **[AUTO-POPULATE]**	**Answer Options:**	**Type:**
1. Have your [ANIMAL]s been vaccinated for [DISEASE] in the past 12 months?	YES ....1<br>NO ....0 → Skip to Question 5	1
2. Who participates in vaccinating [ANIMAL] for [DISEASE]? Check all that apply:	1. Family roster listed<br>2. Neighbor or family friend<br>3. Veterinarian<br>4. Government worker<br>5. Paid assistant<br>6. Other: ______	1
3. Who participates in physically purchasing vaccine against [DISEASE] for [ANIMAL]? Check all that apply.	1. Family roster listed<br>2. Neighbor or family friend<br>3. Veterinarian<br>4. Government worker<br>5. Paid assistant<br>6. Other: _________	1
4. Who pays for vaccine against [DISEASE] for [ANIMAL]?	1. Family roster listed<br>2. Government<br>3. NGO	1
5. Do you [NAME] know where to purchase vaccines against [DISEASE] for [ANIMAL]?	YES ....1<br>NO 0	2
6. Do you [NAME] have access to vaccine suppliers against [DISEASE] for [ANIMAL]?	YES ....1 → skip to Question 8<br>NO 0	1

7. Would you [NAME] like to have access to vaccine suppliers against [DISEASE] for [ANIMAL]?	YES ....1<br>NO 0	
8. Which mode of transport is most feasible if you [NAME] were to travel to a vaccine supplier for vaccine purchase? Check all that apply.	1. Car<br>2. Bus<br>3. Motorbike<br>4. Bicycle<br>5. Walk	1
9. Can you [NAME] afford the vaccine against [DISEASE] for [ANIMAL]?	YES ....1<br>NO ....0<br>DON'T KNOW 88	1
10. Do you [NAME] have access to a cold chain for vaccine storage?	YES ....1<br>NO 0	1
11. How many of your [ANIMALS] died from [DISEASE] last year? (FILL IN NUMBER)	[__________]	1
12. Do you [NAME] think that vaccination can prevent [DISEASE] in your [ANIMAL]s?	YES ....1<br>NO ....0<br>DON'T KNOW 88	1
13. When is the best time to administer vaccines against [DISEASE] for [ANIMAL]? (Check all that apply, do not read answers to participant, code based on response)	1. When animal is healthy<br>2. When animal is sick<br>3. When a member of the herd is sick<br>4. When neighbor's animal is sick<br>5. When animal in village is sick<br>6. Other: ___________	2
14. How knowledgeable are you about [ANIMAL] health?	1. Not at all (1)<br>2. Small extent (2)<br>3. Medium extent (3)<br>4. High extent (4)	2
15. Do you [NAME] have access to training seminars about [ANIMAL] health?	YES ....1 NO ....0 →skip to question 17	
16. Have you attended a training session about [ANIMAL] in the past 12 months?	YES ....1<br>NO 0	

DRAFT

17. Do you have access to information on vaccinating [ANIMAL] for [DISEASE]?	1. Not at all (1)<br>2. Small extent (2)<br>3. Medium extent (3)<br>4. High extent (4)	2
18. Can farmers such as yourself vaccinate [ANIMAL] against [DISEASE]?	YES ....1<br>NO ....0<br>DON'T KNOW 88	2
19. Are there regulations about who is allowed to vaccinate [ANIMAL] against [DISEASE]?	YES ....1<br>NO ....0<br>DON'T KNOW 88	2
20. Does the government play a role in vaccinating [ANIMAL] against [DISEASE]?	YES ....1<br>NO ....0<br>DON'T KNOW 88	2

END OF QUESTIONAIRE

DRAFT
